# How does stochasticity in learning impact the accumulation of knowledge and the evolution of learning?

**DOI:** 10.1017/ehs.2026.10044

**Published:** 2026-04-06

**Authors:** Ludovic Maisonneuve, Laurent Lehmann

**Affiliations:** Department of Ecology and Evolution, University of Lausanne, Lausanne, Switzerland

**Keywords:** cumulative culture, gene-culture coevolution, learning, social learning, stochasticity

## Abstract

Learning is crucial for humans and other animals to acquire knowledge, enhancing survival and reproduction. In particular, individual and social learning allow populations to accumulate knowledge across generations. Here, we examine how stochasticity in the production and social acquisition of knowledge influences the evolution of learning schedules and cumulative knowledge. Using a mathematical model where learning is stochastic, we show that learning stochasticity enhances cumulative knowledge by generating variability in knowledge levels. This allows selection to enhance population knowledge: individuals who acquire more knowledge by chance are more likely to survive and reproduce, and therefore to transmit their knowledge to the next generation. As knowledge accumulates, social learning exemplars tend to possess more of it, favouring greater time investment in social learning. Because social learning provides access to substantially more knowledge when learning is stochastic, selection also favours the evolution of greater investment into learning, at the expense of a fecundity cost. Moreover, when knowledge enhances fecundity but not survival, learning stochasticity favours learning from parents rather than other adults, because learning stochasticity increases uncertainty about exemplar knowledge, making parenthood a cue for possessing fecundity-enhancing knowledge. Finally, when learning occurs predominantly from parents, learning stochasticity itself is favoured by selection.

## Graphical abstract

**Figure:**
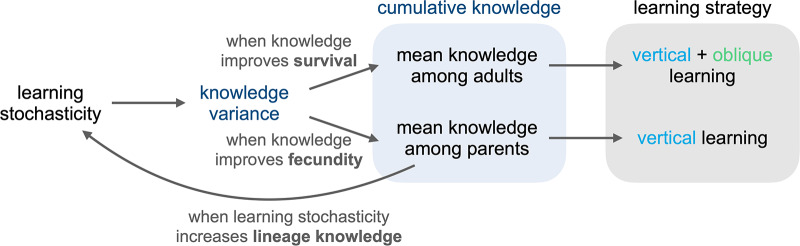

## Social media summary

How does knowledge accumulate across generations, and what shapes how individuals learn and from whom they learn? Using a mathematical model, we show that stochasticity in learning generates variation in knowledge, strengthening cultural selection, increasing cumulative knowledge, and shaping the evolution of learning strategies and transmission pathways.

## 1. Introduction

Individuals use knowledge to perform behaviours that enhance survival and reproduction. This knowledge can be acquired either through personal experience (individual learning), such as trial-and-error learning (e.g., Dugatkin 2008, Ghirlanda and Lind 2017), or from other individuals (social learning), for example, through imitation (e.g., Zentall 2006, Dugatkin 2008, Bates and Byrne 2010). Social learning can occur through a wide variety of pathways (Cavalli-Sforza and Feldman, 1981, Boyd and Richerson, 1985, Laland, 2004, van Schaik, 2016, Kendal et al., 2018, Camacho-Alpízar and Guillette, 2023), including, for instance, vertical transmission (from parent to offspring) and oblique transmission (from unrelated older individuals). The way individuals allocate resources to different learning behaviours across their lifetime affects the population dynamics of knowledge and can support its gradual accumulation and refinement across generations. This process, known as cumulative knowledge or cumulative culture, is widely regarded as a key factor in the ecological success of humans (Henrich, 2015, van Schaik, 2016), and growing evidence suggests that it may also contribute to adaptive behaviours in non-human animals (Hunt and Gray, 2003, Sasaki and Biro, 2017, Jesmer et al., 2018, Gunasekaram et al., 2024).

Learners’ individual and social learning strategies influence changes in knowledge within a population by shaping *cultural deviation* and *cultural selection*, the two key mechanisms underlying population change in any cultural trait and understood here as a socially transmissible trait (see Henrich and Boyd, 2002, El Mouden et al., 2014, Aguilar and Akçay, 2018, Nettle, 2020, Mesoudi, 2021). First, cultural deviation occurs when the learning process, on average, leads to differences between the knowledge of learners and that of their exemplars. This mechanism can either promote knowledge accumulation, for example, when learners produce new or refine existing knowledge by individual learning after social learning (e.g., Enquist et al., 2008, Aoki et al., 2012a, Nakahashi, 2013, Kempe et al., 2014, Wakano and Miura, 2014, André and Baumard, 2020, Denton et al., 2023), or constrain it, as when learners tend to acquire a lower level of knowledge than their exemplars (e.g., Henrich and Boyd, 2002, Henrich, 2004). Second, cultural selection arises when some individuals serve as exemplars more frequently, amplifying the transmission of their specific knowledge or cultural trait and generating variation in the transmission success of different knowledge or cultural variants (Cavalli-Sforza and Feldman, 1981, Boyd and Richerson, 1985, Micheletti, 2020). Cultural selection may arise from either (i) non-random exemplar choice or (ii) differences in survival and reproduction among individuals with different knowledge or cultural traits (though some authors use the term cultural selection specifically for mechanism (i); Cavalli-Sforza and Feldman, 1981, Mesoudi, 2011). Cultural selection is generally expected to support cumulative knowledge, as it tends to favour cultural variants that are more well-adapted to environmental conditions, for example, when individuals with more adaptive cultural variants tend to be chosen as learning exemplars (e.g., Henrich, 2004, Powell et al., 2009, Kobayashi and Aoki, 2012), or when such variants enhance survival and fecundity, allowing them to spread by increasing opportunities for oblique (e.g., Nakahashi, 2010) and vertical transmission (e.g., Tureček et al., 2019), respectively. Mathematical models have shown that the strength of cultural selection, and thus its potential to drive cumulative knowledge, increases with population variance in cultural traits (Cavalli-Sforza and Feldman, 1981, Boyd and Richerson, 1985).

In turn, knowledge dynamics shape what individuals can acquire through social learning, thereby influencing selection pressures on the allocation of resources across different types of learning behaviour. This feedback between knowledge accumulation and learning strategies gives rise to complex coevolutionary dynamics, also shaped by trade-offs between learning and other functions essential for survival and reproduction (for empirical evidence on such trade-offs, see Mery and Kawecki, 2004, Burger et al., 2008, Snell-Rood et al., 2011, Jaumann et al., 2013, Kotrschal et al., 2013, Christiansen et al., 2016, Evans et al., 2017, Padamsey and Rochefort, 2023). A large body of theoretical work has examined these dynamics and how ecological factors influence the allocation of resources to different forms of learning and the emergence of cumulative knowledge (Nakahashi, 2010, 2013, Aoki et al., 2012b, Lehmann et al., 2013, Wakano and Miura, 2014, Kobayashi et al., 2015, 2016, Mullon and Lehmann, 2017, Ohtsuki et al., 2017, Maisonneuve et al., 2025). Nevertheless, the effect of cultural selection is generally neglected in these models (with few exceptions such as Kobayashi et al., 2016), often because these models assume a deterministic knowledge acquisition process at the individual level, which removes variation that can be selected upon.

However, cultural selection is likely to influence the coevolution of cumulative knowledge and learning strategies, as learning is inherently stochastic; for example, the success of trial-and-error learning often depends on chance discoveries of adaptive cultural variants, leading to individual variation in knowledge. While not their primary focus, Kobayashi et al. (2016) showed that stochasticity in individual learning promotes investment in social learning by amplifying the effect of cultural selection (caused by knowledge-based choice of social exemplars), thereby increasing overall population knowledge and increasing what can be acquired socially. However, in contrast to the assumptions of Kobayashi et al. (2016), individuals in natural populations may not always be able to reliably assess the knowledge of others and to use it to guide their social learning decisions (Argyle and McHenry, 1971, Lutz and Keil, 2002, Wood et al., 2012, Jiménez and Mesoudi, 2019, Hirel et al., 2025). This underscores the need to investigate how stochasticity in learning affects the coevolution of cumulative knowledge and learning strategies without knowledge-based choice of social exemplars.

Furthermore, there is a lack of predictions about how stochasticity in learning affects the evolution of the choice among transmission pathways (e.g., vertical vs. oblique), or the trade-offs between learning and other functions essential for survival and reproduction. Stochasticity in learning could affect these features because it increases uncertainty about the knowledge held by potential exemplars. Previous models have shown that, under such uncertainty, selection favours learning knowledge that affects fecundity from parents rather than from other adults (McElreath and Strimling, 2008). This suggests that stochasticity in learning may play an important role in shaping the allocation between vertical and oblique transmission.

In this study, we examine how stochasticity in learning influences knowledge accumulation by developing an evolutionary model in which learning is described as a stochastic process. The model tracks the evolution of the overall resource allocation to learning and fecundity, as well as the allocation of time across different types of learning (vertical, oblique, and individual learning). This allows us to examine how stochasticity influences the evolution of learning strategies, specifically, the pathways individuals use to acquire information through social learning and the trade-off between investment in learning and reproduction. By allowing knowledge to accumulate over generations, our framework also captures the coevolutionary feedback between learning strategies and knowledge accumulation.

## 2. Model

### 2.1. Life-cycle

**Figure 1.**
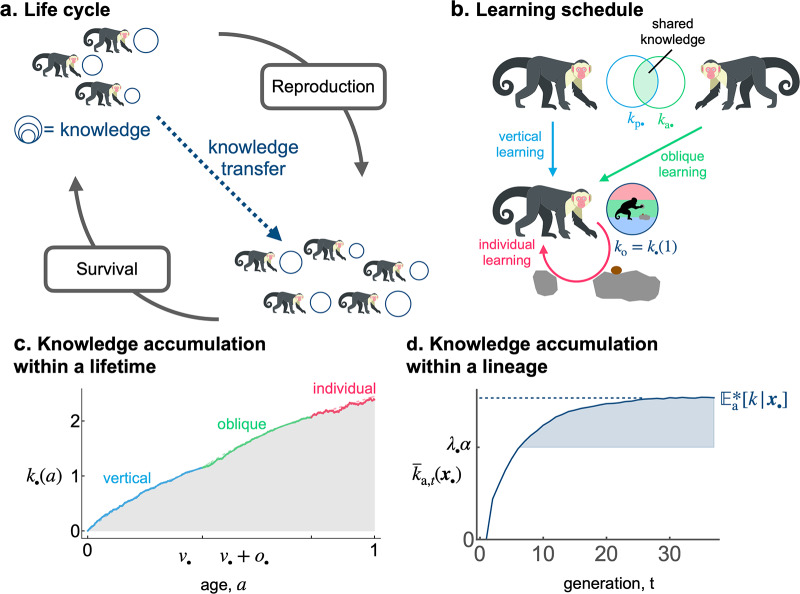
Model overview. (a) Illustration of the life cycle. (b) Illustration of the learning process. A focal individual can obtain knowledge (e.g., the skill set to crack nuts open, denoted by 
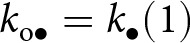
 and represented here as a round set) by learning from three sources: (i) vertically from its parent (with knowledge 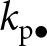; blue arrow); (ii) obliquely from a randomly selected adult (with knowledge 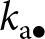, the knowledge of the parent 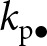 and the oblique exemplar 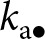 can also overlap and thus be redundant; green arrow); and (iii) individually, when it produces its own knowledge (in pink). See the main text in Section 2.2.2 for more details. (c) A realisation of knowledge accumulation with a lifetime: individual knowledge 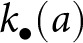 of a focal offspring against its age 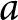 (realisation of the stochastic process defined by eq. (2) with traits 
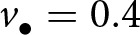
, 
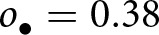
 and 
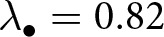
 for the offspring; and parameters 
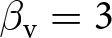
, 
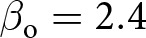
, 
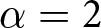
, 
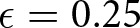
, 
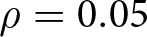
, 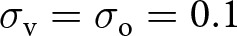, 
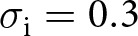
, 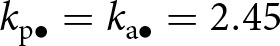). The dashed line shows knowledge accumulation in the absence of stochasticity in learning, that is, when 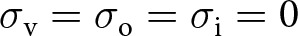. (d) Knowledge accumulation within a lineage: mean’s adult knowledge 
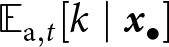
 within an 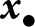-lineage at each generation 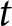 (obtained from an individual-based simulation using the same parameters as in panel c, with trait mutation turned off and starting with a population of one ancestral individual with no knowledge, we set 
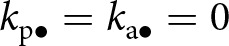
 for the ancestral individual, with 
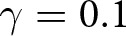
, 
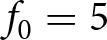
, 
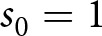
, 
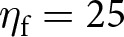
, 
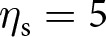
, 
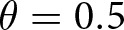
); see Appendix D for more detail on individual-based simulations). The shaded area corresponds to cumulative knowledge (where individuals, on average, possess more knowledge than they could acquire through individual learning alone, i.e., where 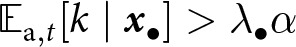). The dashed line shows the expected knowledge of a random adult of an 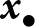-lineage at equilibrium 
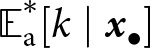
 predicted by our analysis (see Section 2.3).

We consider a large, asexual population where individuals acquire information that enhances fecundity and survival. This includes information such as the location of food sources, the edibility of different foods, or instructions on how to build and use a tool. Each individual possesses a quantity of adaptive information, referred to as knowledge, which is treated as a quantitative variable in our analysis. In each generation, the population goes through the following life-cycle events (see Fig. 1a). (1) Adults produce offspring according to a Poisson process with a mean depending on their knowledge. (2) Offspring acquire knowledge socially from adults and through individual learning through a stochastic learning process. (3) Parents die. Offspring go through a density-dependent survival stage, where their knowledge affects survival. Those surviving become the adults of the next generation, and the cycle starts again. As a result of reproduction and survival, the number of individuals is not fixed and may vary across generations.

During stage (2) of the life cycle, offspring perform sequentially three different types of learning: first, they learn from their parent (vertical learning), then from a randomly selected adult (oblique learning), and finally by themselves (individual learning; see Fig. 1b). All individuals learn during a fixed time, which we normalise to 1. Accordingly, the time allocated to vertical, oblique, and individual learning must sum to one. Two evolving traits shape how individuals allocate their time across the three different types of learning: the amount of time 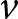 spent learning vertically (Table 1 for a list of symbols); the amount of time 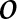 spent learning obliquely; so that 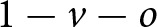 is spent learning individually.

**Table 1.** Key symbols and their definitions

Model parameters	
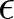	Rate of knowledge obsolescence across generations
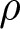	Average overlap in knowledge between two adults
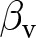 , 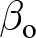	Vertical and oblique transmission rate per fraction of investment allocated to learning
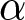	Individual learning rate per fraction of investment allocated to learning
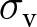 , 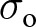 , 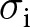	Intensity of stochastic effects in vertical, oblique, and individual learning
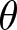	Fecundity cost exponent
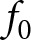 , 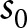	Baseline fecundity and baseline survival modulator
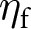 , 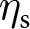	Conversion factor that translates knowledge into fecundity and survival benefits
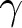	Strength of density-dependent competition

Moreover, we assume that an evolving trait 
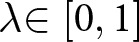
 controls the overall trade-off between allocating resources to learning and fecundity. Higher values of 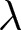 enhance the efficiency of all learning types but at the cost of reduced fecundity. For instance, 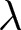 may reflect developmental costs or the metabolic expenditure associated with learning ability.

Next, we describe in detail how traits affect the three life cycle stages (Section 2.2) and then outline the approach used to analyse the cultural and the evolutionary dynamics (Section 2.3).

### 2.2. Traits effects and knowledge throughout the life-cycle

#### 2.2.1. Adult reproduction

We start by considering a focal adult from an arbitrary generation, characterised by traits 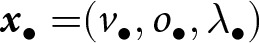 and amount of knowledge 
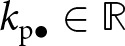
. This knowledge is the realised outcome of a stochastic learning process experienced during the individual’s offspring stage (detailed in the next section). The expected number of offspring of the focal adult, referred to as fecundity, is given by
(1)
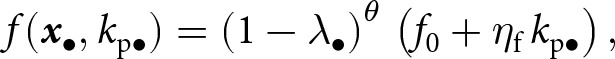



where 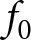 is the baseline fecundity without investment into learning, and 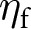 is a conversion factor translating knowledge into fecundity. For example, in a context where knowledge enhances offspring care, 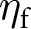 would be high. An individual’s fecundity is reduced by its investment 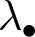 in learning, reflecting costs associated with acquiring learning abilities, as well as the metabolic expenses involved in the learning process itself. The parameter 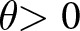 controls the strength and shape of this fecundity penalty: higher values of 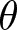 amplify the fecundity cost of learning, while lower values make this cost more gradual. This implementation of the learning–fecundity trade-off differs from earlier models, in which the trade-off between learning and reproduction is typically expressed through the allocation of time among learning and other functions (Lehmann et al., 2010, 2013, Wakano and Miura, 2014, Mullon and Lehmann, 2017).

#### 2.2.2. Offspring learning

Next, we consider an offspring of the focal adult. This focal offspring inherits the parent’s traits 
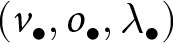
, barring mutation. Let us denote by 
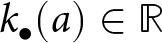
 the knowledge this offspring bears at age 
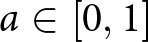
 (where 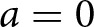 is birth). Building on the models of Kobayashi et al. (2016) and Maisonneuve et al. (2025) (where differences with our model are outlined in Appendix A.1), we model learning as a continuous-time stochastic process, in which individuals acquire knowledge at a deterministic rate on average, but this accumulation is subject to random fluctuations. Specifically, the knowledge 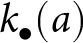 of the focal offspring at each age 
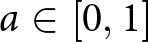
 is a realisation of the following stochastic differential equation
(2)
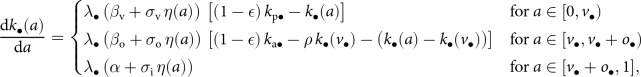
where the initial condition is 
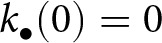
. The term 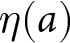 is the standard white noise term from stochastic calculus that introduces rapid and highly irregular fluctuations in the learning process at each age 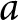, with zero mean and no temporal correlation (Gardiner, 1985, chapter 4.1). Each realisation of the random white noise term 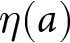 over 
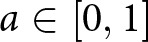
 determines a corresponding realisation of knowledge acquisition 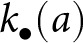 over 
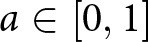
 (e.g., Fig. 1c).

Equation (2) says that the focal offspring first learns from its parent. Vertical learning occurs over a duration of length 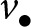, during which the focal offspring acquires knowledge instantaneously at a rate proportional to a stochastic vertical transmission rate, 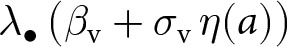, which increases with the net investment in learning 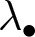. The term 
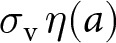
 describes the impact of stochastic events during vertical learning at age 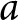, where the parameter 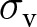 controls the magnitude of the impact of learning stochasticity. When 
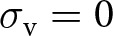
, learning occurs with no stochastic fluctuations. The knowledge acquisition rate is assumed to be proportional to the difference 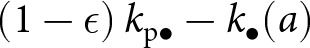, between the knowledge currently available 
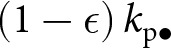
 from the parent and the focal offspring’s knowledge 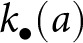, where 
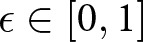
 is the proportion of knowledge that becomes obsolete between two generations. Indeed, as the amount of available knowledge decreases, the focal offspring is less likely, on average, to encounter new information during interactions with its parent, thereby slowing the learning process.

Secondly, the focal offspring learns from a random adult, whose knowledge is denoted by 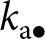, for a duration of length 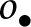. Similar to the parent’s knowledge, 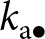 results from a realisation of the stochastic learning process undergone by the oblique exemplar in the previous generation (see details below). During oblique learning, the instantaneous knowledge acquisition rate is proportional to the product of a stochastic oblique transmission rate, 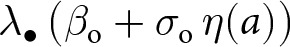, and of the amount of knowledge held by the oblique exemplar that the focal offspring has yet acquired, which is assumed to be given by 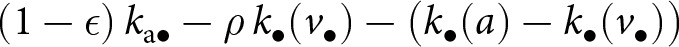. This expression accounts for both the overlap between the parent’s and the exemplar’s knowledge, and the knowledge the offspring has already acquired obliquely. This expression assumes that, on average, the knowledge held by two adults in the population overlaps by a proportion 
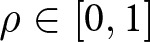
. Consequently, the knowledge of the parent and of the oblique exemplar also overlaps by the same proportion 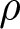. At the end of the vertical learning phase (i.e., at age 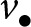), the focal offspring has acquired from its parent a quantity of knowledge 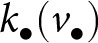, of which a quantity 
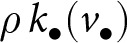
 is also known by the oblique exemplar. The total amount of knowledge that the focal offspring can potentially acquire from the oblique exemplar is therefore 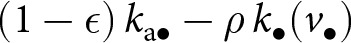. At age 
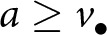
, the focal offspring has already acquired from the oblique exemplar a quantity of knowledge 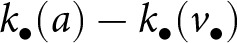, which is precisely the amount gained beyond what was acquired from the parent. Hence, the remaining knowledge available from the oblique exemplar at age 
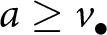
 is 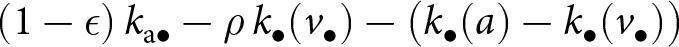. Note that the average overlap in knowledge between two adults, 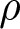, may reflect environmental features. For instance, environmental heterogeneity can lead individuals to produce different knowledge that addresses different ecological challenges, thereby reducing overlap.

Thirdly, the focal offspring learns individually at an instantaneous stochastic rate proportional to 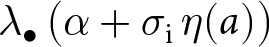 for the remaining 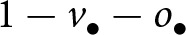 time. The parameter 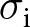 captures the amplitude of stochastic variation in individual learning, reflecting, for example, fluctuations in attention or intrinsic randomness in the mechanistic processes underlying learning, such as trial-and-error. During each learning phase, the rate of knowledge acquisition is proportional to the offspring’s net investment in learning 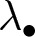. As a result, when there is no such investment (i.e., 
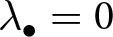
), the focal offspring does not acquire any knowledge at any age (i.e., 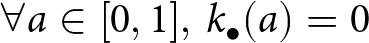). Due to stochasticity, the learning rate may occasionally become negative during each learning phase, implying that the offspring may lose knowledge. This could reflect situations where miscommunication or exploration leads to confusion, or replacement of previously held information by incorrect alternatives.

Note that both the knowledge of the parent 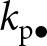 and that of the oblique exemplar 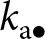 of the focal offspring result from realisations of the stochastic learning process experienced by those individuals in the previous generation. Specifically, their knowledge is obtained as an instantiation of 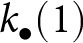, where 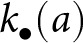 denotes a realisation at age 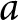 of the stochastic differential equation defined in eq. (2), with the values of 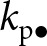 and 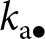 on the right-hand side corresponding to the knowledge values of the parent’s own parent and oblique exemplar, and, in the case of the focal’s oblique exemplar, to those of its own learning exemplars. Since both reproduction and survival are affected by knowledge, the realised parental knowledge 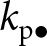 and oblique exemplar knowledge 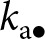 among the offspring are not solely determined by the stochastic learning process defined in eq. (2), but also by reproduction and survival, and thus by offspring survival, which we next specify.

#### 2.2.3. Offspring survival

After completing their learning, the offspring enter a density-dependent survival stage. Let 
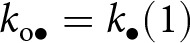
 denote the knowledge acquired by the focal offspring at the end of the learning phase, where 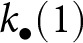 is the outcome of eq. (2). The probability of survival of the focal offspring is given by
(3)
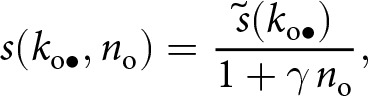
where 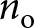 is the total number of offspring produced by all adults and 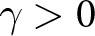 is a parameter controlling the strength of density dependence. The numerator 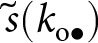, which modulates survival probability, is given by
(4)
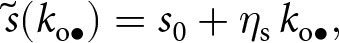
where 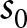 is the baseline survival, and 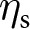 is the conversion factor that translates knowledge into survival. For example, in a context where knowledge enables predator recognition, 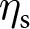 would be high. Parameter values are chosen in our analyses such that 
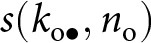
 remains between 0 and 1 for all individuals.

### 2.3. Analyses

Here, we detail the hypotheses and the method we employ to investigate the joint cultural and evolutionary dynamics. Assuming a small mutation rate and a large population size, evolutionary change proceeds more slowly than cultural dynamics. This timescale separation allows us to analyse cultural dynamics while treating population trait values as constant and consider the knowledge dynamics within lineages of individuals bearing the same trait values (Mullon and Lehmann, 2017).

#### 2.3.1. Cultural dynamics

We first aim to describe the equilibrium probability density of knowledge for a member of an 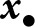-lineage, which is shaped both by the stochastic learning process defined in eq. (2), which enables both the production and intergenerational accumulation of knowledge, and by the effects of knowledge on fecundity and survival given in eqs. (1) and (3), since individuals who survive and reproduce are more likely to transmit their knowledge. In general, the learning process is too complicated to obtain an explicit expression for the probability density of knowledge. To make the analysis tractable, we assume that the outcomes of social learning are deterministic, in contrast to individual learning, where we assume that producing knowledge is inherently stochastic (i.e., 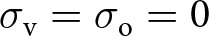, 
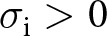
). This assumption is relaxed in individual-based simulations, which allow stochasticity in social learning and recover the same qualitative effects of stochasticity in learning on knowledge accumulation and traits evolution (Fig. S.10). Under this assumption, we can solve the stochastic differential equation (2) (see Appendix A.2). Using this solution, along with the effects of knowledge on fecundity and survival given in eqs. (1) and (3), we derive a recursion for the expected knowledge and its variance for a member of an 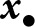-lineage (see Appendices A.3.1 and A.3.2). However, this recursion ultimately depends on the entire hierarchy of moments of the probability density of knowledge. To address this issue and be able to track the expected knowledge and its variance across generations, we employ a Gaussian closure approximation (see Appendix A.3.3). Note that the stationary distributions obtained from individual-based simulations suggest that a Gaussian approximation provides a good fit for the probability density of knowledge (see Fig. S.1). With this, we can then fully characterise the cultural equilibrium in terms of the expected knowledge and its variance (see Appendix A.4).

#### 2.3.2. Evolutionary dynamics

Under the assumptions of small mutation rate, small variance in knowledge, and a large population, the expected evolutionary dynamics can be inferred from the expected lineage fitness in a focal 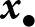-lineage, i.e., the expected fitness of a random individual in that lineage (Mullon and Lehmann, 2017). We show in Appendix B.1 that the expected lineage fitness of an 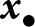-lineage in a population with mean traits 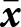 can be expressed as
(5)
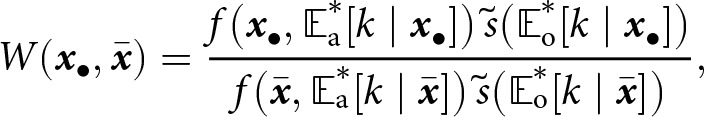
where 
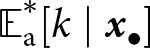
 and 
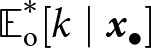
 are the expected knowledge after learning is complete and at cultural equilibrium for a random adult and a random offspring of an 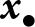-lineage. As we assume small variance in knowledge, 
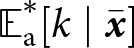
 and 
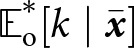
 coincide with the population mean adult and offspring knowledge.

The expected evolutionary dynamics can then be inferred from the selection gradient, defined as
(6)
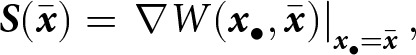
where the operator 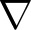 acts such that, for any function of traits 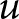, we have
(7)
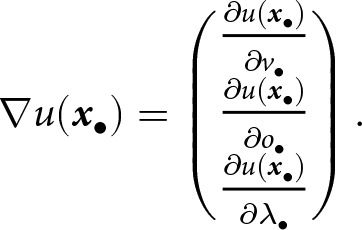


Each entry of the selection gradient indicates whether natural selection favours an increase or a decrease in the corresponding trait. According to invasion analysis (Leimar, 2009), the mean trait values 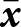 will eventually converge to a convergence stable trait vector denoted by 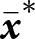, which either satisfies 
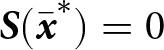
 or lies on the boundary of the phenotypic space. We use the expression of the selection gradient to highlight the distinct selective pressures acting on each learning trait and to numerically estimate the trait values 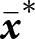 favoured by selection starting from a population initially lacking any form of learning, that is, with ancestral traits 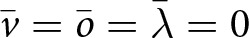 (see details in Appendix B.3). We thus focus only on the convergence stable trait vector reached from these ancestral trait values, as this corresponds to the biologically relevant scenario in which learning must first evolve before cultural dynamics operate. At 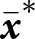, we systematically verify if selection is stabilising (see details in Appendix B.3), ensuring the maintenance of a unimodal trait distribution. In all analyses, selection was found to be stabilising.

To assess the robustness of our forthcoming analytical findings, we perform individual-based simulations that relax the previously stated assumptions (see details of individual-based simulations in Appendix D). In particular, we allow for small population sizes, allow 
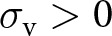
 and 
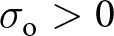
, and no longer assume that the probability density of knowledge is normal.

## 3. Results

### 3.1. Cultural dynamics

#### 3.1.1. Cultural selection enhances cumulative knowledge

To reveal the effects of cultural selection on cumulative expected knowledge, we analyse the dynamics of knowledge in a given lineage. To this end, we first determine the realised knowledge 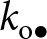 acquired by a focal offspring in an 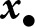-lineage, whose parent has knowledge 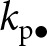 and who chooses an oblique exemplar with knowledge 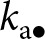. We show in Appendix A.2 that
(8)
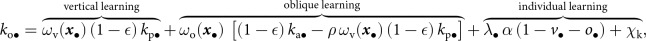
where 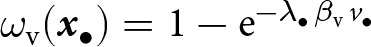 and 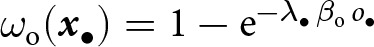 are the proportion of available knowledge at the start of the vertical and oblique learning phases, respectively, that the focal offspring acquires. Each term in eq. (8) corresponds to the amount of knowledge acquired through each type of learning. The knowledge gained through vertical learning reduces the knowledge available through oblique learning, since parental knowledge overlaps with that of the oblique exemplar with proportion 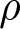. As the offspring already acquired an amount 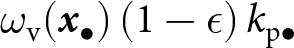 of knowledge from its parent, the amount of non-redundant knowledge available at the start of oblique learning is 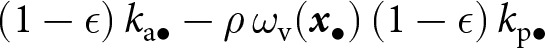. Finally, the knowledge acquired through individual learning 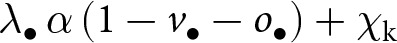 depends on a realisation of a Gaussian random variable 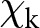. This random variable has mean zero and variance 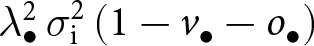, which is proportional to the time allocated to individual learning 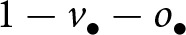.

Having characterised the realised knowledge acquired by a focal offspring, we can now characterise the lineage-level dynamics at which cultural selection acts. Specifically, by taking the expectation in eq. (8), we show in Appendix A.3.1 that the expected knowledge 
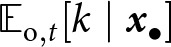
 of an offspring born to an adult of generation 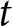 of an 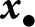-lineage, when the expected value and variance of knowledge among adults are 
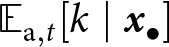
 and 
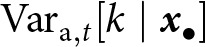
, is given by
(9)
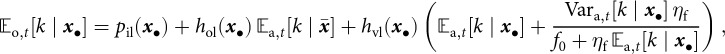
where 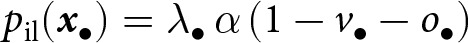, is the expected amount of knowledge produced through individual learning by the focal offspring, and 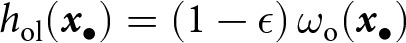 and 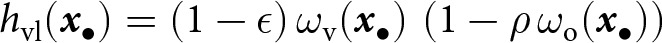 are the oblique and vertical cultural heritabilities, respectively. The expressions for 
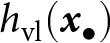
 and 
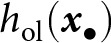
 are obtained by identifying, in eq. (8), the coefficients multiplying parental knowledge 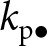 and oblique exemplar knowledge 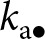 in the realised focal offspring knowledge 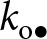. The term 
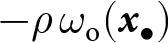
 in 
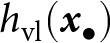
 captures that, when parental and oblique knowledge overlap, knowledge acquired from the parent reduces the amount of knowledge that remains available through oblique learning. This interference effect reduces vertical, but not oblique, cultural heritability, because it scales with the amount of parental knowledge (see eq. (8)). Intuitively, this term corrects the vertical heritability 
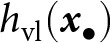
 by accounting that a part of the knowledge acquired from the parent substitutes for knowledge that would otherwise have been acquired from oblique exemplars. The oblique and vertical cultural heritabilities, both lying between 0 and 1, can then be interpreted as the proportion of knowledge from the oblique and vertical exemplar that is effectively transmitted to a learner with traits 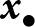.

The last two terms on the right-hand side of eq. (9) are the expected knowledge acquired from an oblique exemplar and from the parent. These terms depend on the corresponding cultural heritabilities, multiplied by the expected knowledge 
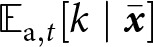
 of an oblique exemplar and that 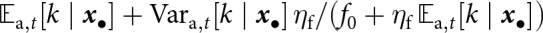 of a vertical exemplar. The expected knowledge of a vertical exemplar exceeds that of an average adult in the lineage, since individuals with greater knowledge produce more offspring and are overrepresented among vertical exemplars.

The term 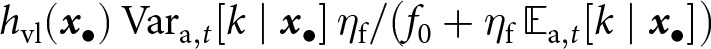 in (9) can be thought of as the response of expected knowledge to cultural selection (in analogy with the response to selection due to genetic inheritance; Lynch and Walsh, 1998), due to differences in fecundity. Because individuals with greater knowledge tend to have higher fecundity, they are more likely to transmit their knowledge vertically, which biases transmission towards higher knowledge individuals and increases the expected knowledge within the lineage. The response to cultural selection is particularly pronounced when parents transmit a substantial portion of their knowledge to their offspring (i.e., 
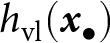
 is great), there is significant knowledge difference between adults (i.e., 
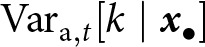
 is great), and knowledge strongly increases fecundity (i.e., 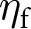 is great). The strength of the response to cultural selection diminishes as relative differences in fecundity with the lineage become less pronounced with increasing values of 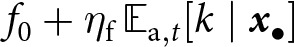.

Following learning, the expected knowledge within a lineage is further shaped by survival to adulthood. After completing their learning, offspring go through the survival stage to reach adulthood in generation 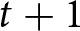. We show in Appendix A.3.1 that the expected knowledge 
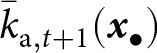
 of an adult of generation 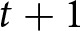 in the 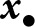-lineage is
(10)
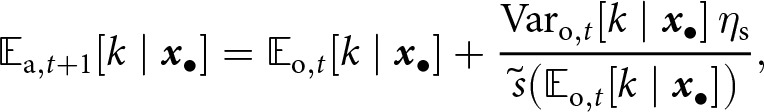
where 
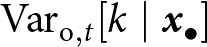
 is the variance in the knowledge held by a randomly chosen offspring born to an adult of generation 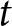 in the 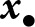-lineage. The expected knowledge among adults is higher than that among offspring (i.e., 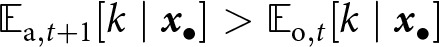) since offspring with above-average knowledge are more likely to survive to adulthood. This effect is captured by the selection differential 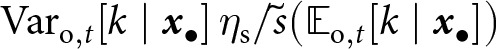, which is the difference between expected knowledge of surviving and all offspring within the lineage. This selection differential is particularly pronounced when there is a significant knowledge difference between offspring (i.e., 
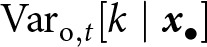
 is great) and knowledge strongly increases survival (i.e., 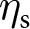 is great). The selection differential diminishes as relative differences in survival within the lineage become less pronounced with increasing values of 
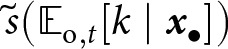
. The response of an individual’s expected knowledge to cultural selection due to variation in survival depends on how knowledge is transmitted to the new offspring cohort. It equals 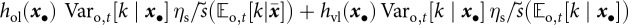 (obtained by substituting the expression of 
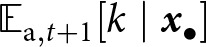
 from eq. (10) and 
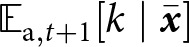
 from eq. (10) with 
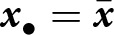
 into eq. (9) with 
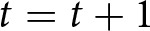
 and identifying the terms corresponding to cultural selection).

Taken together, eqs. (9) and (10) allow us to characterise the expected knowledge at equilibrium. We focus on the expected knowledge in adults only, as this is sufficient to uncover the mechanisms shaping expected knowledge. We show in Appendix A.4.1 that the equilibrium expected adult knowledge in the 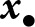-lineage satisfies
(11)
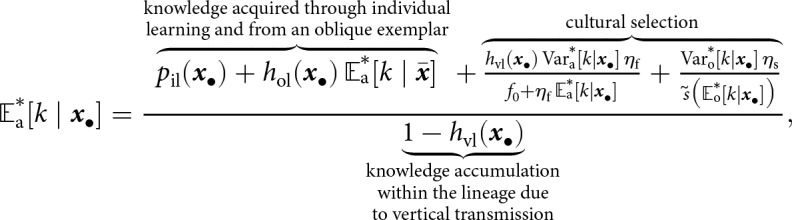
where the superscript 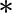 indicates that the quantities are evaluated at equilibrium (see also Fig. 1d). The first two terms in the numerator of eq. (11) are the expected amount of knowledge acquired through individual and from an oblique exemplar. The remaining terms in the numerator are the increase in expected knowledge driven by cultural selection resulting from differences in fecundity and survival within the lineage due to variation in knowledge. The denominator of eq. (11) captures the inter-generational accumulation of knowledge within the lineage due to vertical transmission. This can be seen by expressing 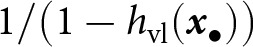 as 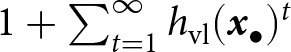, where 
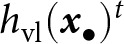
 is the proportion of knowledge acquired by an ancestor 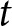 generations ago that is effectively transmitted to the focal individual. Since 
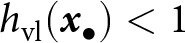
, the cumulative contribution of ancestors remains bounded.

We find that offspring exhibit a slightly lower expected knowledge than adults (i.e., 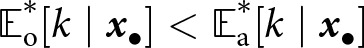; see Fig. S.2) because offspring with above-average knowledge are more likely to survive and become adults. In contrast, offspring and adults have the same expected knowledge (i.e., 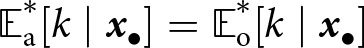; see Fig. S.2) when knowledge does not affect survival to adulthood (i.e., 
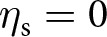
).

#### 3.1.2. Learning stochasticity enhances knowledge by increasing knowledge variance

Our results reveal that cultural selection promotes knowledge accumulation. Because the strength of cultural selection increases with knowledge variance (see eq. (11)), we next investigate the mechanisms shaping this variance. We focus on variance in adults knowledge only, as this is sufficient to identify the mechanisms shaping variance in knowledge. In Appendix A.4.2, we show that the equilibrium variance in the knowledge held by an adult from the 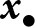-lineage satisfies
(12)
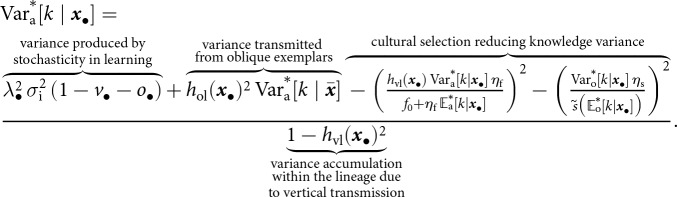


The first term in the numerator of eq. (12) is the knowledge variance produced by stochasticity in individual learning. This term increases with the level of learning stochasticity 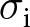, investment in learning 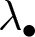, and the time allocated to individual learning 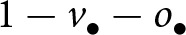. If learning were deterministic (i.e., 
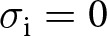
), no knowledge variance would be produced, and the only solution to eq. (12) would be 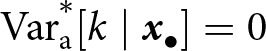 (see proof in Appendix A.4.3). The second term in the numerator accounts for variance transmitted from oblique exemplars. The remaining terms in the numerator are the effect of cultural selection, which reduces knowledge variance. Because survival to adulthood reduces knowledge variance, the equilibrium variance in the knowledge held by an adult 
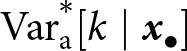
 is generally slightly lower than that of an offspring 
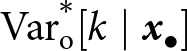
 (see Fig. S.2). When knowledge does not affect survival (i.e., 
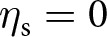
), the two variances are equal (i.e., 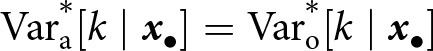; see Fig. S.2). Finally, the denominator of eq. (12) describes the accumulation of knowledge variance within the 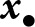-lineage through vertical transmission. This can be seen by expressing 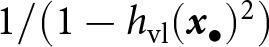 as 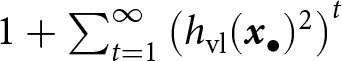, where 
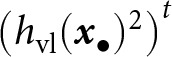
 is the proportion of knowledge variance from lineage members 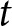 generations ago that is effectively transmitted to current lineage members.

**Figure 2.**
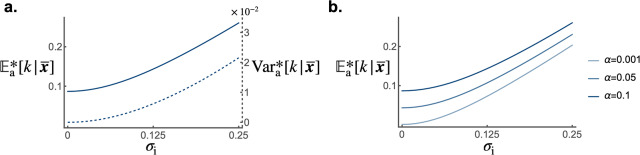
The accumulation of knowledge. (a) Population mean knowledge 
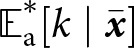
 (solid line) and population knowledge variance 
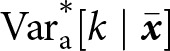
 (dashed line) at cultural equilibrium according to intensity of stochastic effects in individual learning 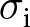 (left axis gives the scale of cumulative knowledge, and right axis gives the scale of knowledge variance). (b) Population mean knowledge 
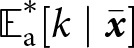
 according to intensity of stochastic effects in individual learning 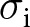 for different individual learning rates per fraction of investment allocated to learning 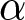. Default parameters are: 
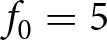
, 
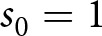
, 
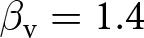
, 
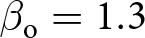
, 
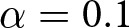
, 
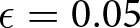
, 
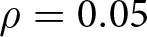
, 
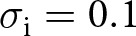
, 
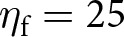
, 
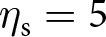
, 
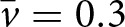
, 
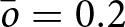
, 
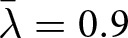
.

Altogether eqs. (11) and (12) reveal that, by generating knowledge variance, stochastic individual learning amplifies cultural selection and enhances cumulative knowledge. By numerically estimating 
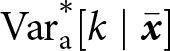
 and 
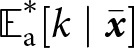
 (see Appendix A.4.4 for details on the procedure) for different values of 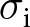, we confirm that increased stochasticity in individual learning 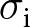 increases population knowledge variance 
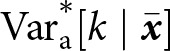
, which in turn lead to an increase in mean knowledge 
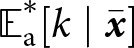
 (Fig. 2a).

Remarkably, stochasticity in individual learning 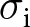 can foster knowledge accumulation even in populations with low average knowledge-production ability (e.g., 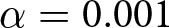; see Fig. 2b). When stochasticity is particularly high, cultural selection becomes the dominant force driving knowledge acquisition. As a result, populations with different average knowledge-production abilities tend to carry similar levels of mean knowledge, whereas, in the absence of stochasticity, they exhibit marked differences in mean knowledge (see Fig. 2b). Additionally, because knowledge improves both fecundity and survival, greater stochasticity in individual learning 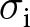 is associated with larger population size (see Fig. S.3). The effect of cultural selection in enhancing the mean knowledge 
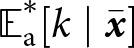
 is stronger when knowledge strongly increases fecundity and survival (i.e., when 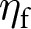 and 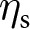 are higher; see Fig. S.4), and when vertical and oblique cultural heritability are higher (Fig. S.5).

### 3.2. Evolutionary dynamics

#### 3.2.1. Trade-off between fecundity and learning

With knowledge distribution at equilibrium characterised, we now use the selection gradient to investigate the effect of selection on the evolution of the learning traits. By substituting eq. (5) into eq. (6), we obtain the direction of selection on the traits
(13)
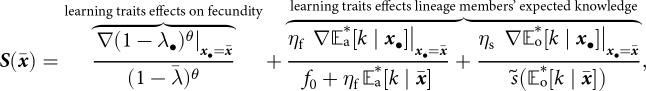
where the gradient operator 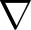 is defined in eq. (7).

The first term in eq. (13) describes selection arising from the fecundity costs associated with investment in learning. The remaining terms describe the effect of selection resulting from the impact of learning traits on the expected knowledge of members of a lineage. The strength of these selection pressures diminishes with increasing values of 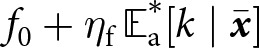 and 
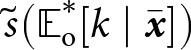
, as relative differences in fecundity and survival among lineages become less pronounced.

Overall, eq. (13) indicates that the evolution of learning traits results from a trade-off between the fecundity costs incurred and the knowledge gained. We focus on evaluating the trait values 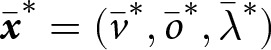 favoured by selection in the long-term starting from a population initially expressing 
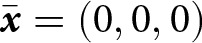
 (see Appendix B.3 for details on the procedure). Numerical estimates of the average learning traits at 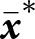 reveal that the fecundity costs can prevent the emergence of learning, particularly when individual learning is inefficient at producing knowledge (i.e., low 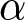; see Fig. S.6a). As 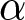 increases, learning can emerge abruptly rather than gradually, because the emergence of learning allows knowledge to accumulate across generations, promoting further social learning and further learning investment in turn.

Fecundity costs also shape the evolution of the learning traits: when the fecundity costs associated with learning investment are higher (i.e., 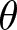 is higher), individuals invest less in learning (i.e., 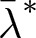 is lower; Fig. S.6b). This rapidly limits the evolution of both types of social learning (i.e., 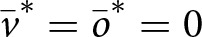; Fig. S.6b). Indeed, when the fecundity cost exponent 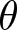 is higher, individuals allocate more resources to reproduction and less to learning at 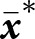 (i.e., 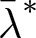 is lower; Fig. S.6b), which restricts knowledge production and consequently reduces the benefits of social learning. In the following numerical analyses, we set 
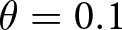
, a value that permits the evolution of social learning.

#### 3.2.2. Trade-off between different types of learning

To better understand the effect of selection on the learning traits, we decompose their effect on the expected knowledge of adults in the 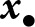-lineage. We show in Appendix B.2 that 
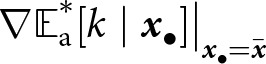
 satisfies
(14)
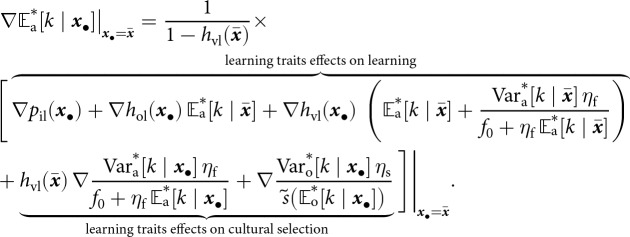
 (Appendix B.2). The terms on the second line describe the impact of learning traits on knowledge acquisition. This marginal effect depends on how traits 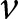, 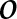, and 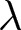 influence the knowledge produced individually, as well as that acquired from oblique and vertical exemplars. The marginal knowledge gained through individual learning is 
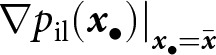
. The marginal knowledge gained from oblique and vertical exemplars depends on the effect of the learning traits on the oblique 
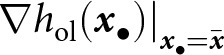
 and vertical 
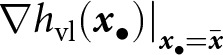
 cultural heritability, multiplied by the expected knowledge of a parent and an oblique exemplar. Since the traits 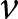 and 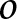 enhance the time allocated to vertical and oblique learning, but reduce the time available for individual knowledge production, their evolution is shaped by trade-offs between different types of learning. The terms on the third line describe the impact of learning traits on cultural selection acting on the expected adult knowledge in the lineage. Learning traits can impact cultural selection by impacting the variance in potential knowledge, as well as the expected fecundity and survival within the lineage.

The selection pressure scales as 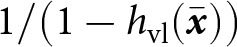, which captures the inter-generational accumulation of the effects of learning traits on knowledge acquisition and on cultural selection, due to vertical transmission. This can be seen by expressing this factor as 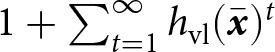, where 
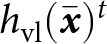
 is the population mean proportion of knowledge initially acquired by an ancestor that is transmitted to a direct descendant 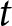 generations later. This formulation highlights that a change in knowledge in one generation, driven by a change in learning traits, influences the knowledge of all future descendants within the lineage. By capturing the cumulative effects of these interactions, which occur among relatives, the evolutionary dynamics thus incorporate multi-generational kin selection effects.

To understand selection on the learning traits, we also need to examine 
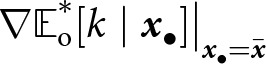
 (see eq. (13)). The term 
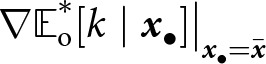
 is equal to 
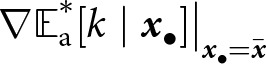
 minus the effect of learning traits on cultural selection due to differences in survival (see eq. (B.25)). This difference arises because offspring have not yet undergone survival themselves.

Altogether eqs. (13) and (14) reveal that the evolution of learning traits is influenced by the trade-off between knowledge acquisition from different learning types. Numerically estimating the mean trait values 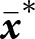 favoured by selection reveals that individuals allocate significant time to both vertical and oblique learning (i.e., high 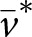 and 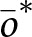; see Fig. S.6c) when knowledge remains relevant over time (i.e., low 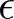; e.g., in a stable environment). Under these conditions, individuals can acquire substantial knowledge socially. Since social learning becomes more efficient in stable environments, individuals tend to invest significant resources into learning (i.e., high 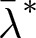; see Fig. S.6c). The trade-off between vertical and oblique learning depends on the overlap in knowledge between two adults 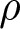. When there is a high knowledge overlap between adults (i.e., high 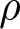), individuals tend to skip the oblique learning phase at 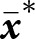 (i.e., 
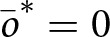
; see Fig. S.6d), since we assume individuals learn more easily from parent (i.e., 
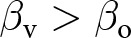
). Conversely, when there is a low knowledge overlap between adults (i.e., low 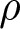), at 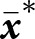 individuals allocate time to oblique learning (i.e., 
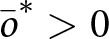
), enabling them to acquire knowledge not available from their parents (see Fig. S.6d). This outcome is consistent with the results of Maisonneuve et al. (2025) (see Appendix B.4 for details). Although it strongly influences the learning schedule, the overlap in knowledge between two adults 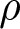 has little effect on resource allocation between learning and fecundity at the reached evolutionary equilibrium (i.e., little effect on 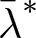; see Fig. S.6d).

#### 3.2.3. Learning stochasticity promotes social learning

**Figure 3.**
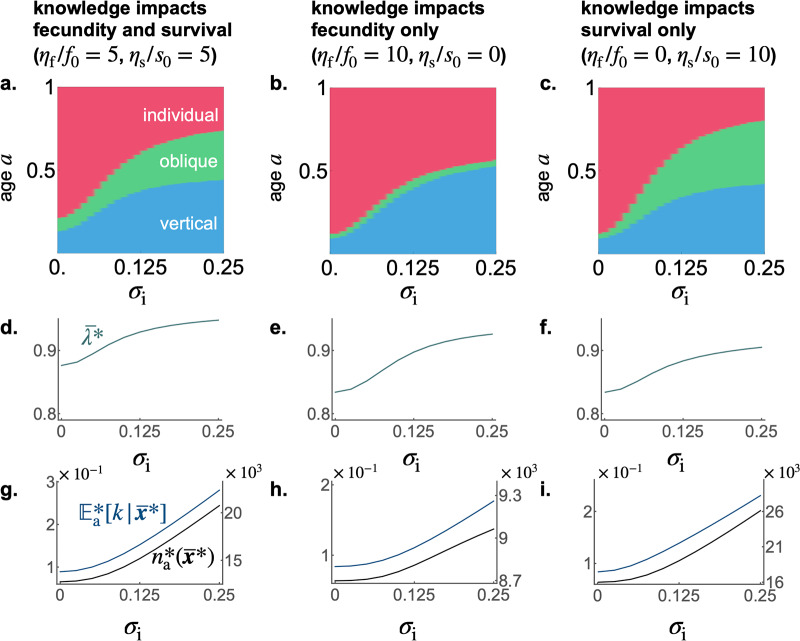
Evolution of learning traits. (a–c) Learning schedule (y-axis) at 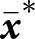 against the intensity of stochastic effects in individual learning 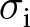 (x-axis) for different values of the conversion factor that translates knowledge into fecundity 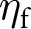 and survival benefits 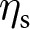. Blue, green, and pink areas represent time spent performing vertical, oblique, and individual learning, respectively. (d–f) Investment in learning 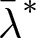 at 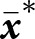 corresponding to panels a–c. (g–j) Population mean knowledge 
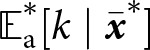
 (blue) and adult population size 
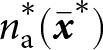
 (black) at 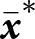 corresponding to panels a–c (left axis gives scale of knowledge, and right axis gives scale of population size, with 
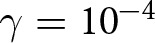
). Default parameters are the same as in Fig. 2 with 
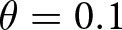
.

We now investigate the impact of stochasticity in individual learning, quantified by the parameter 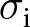, on the evolution of learning traits. We show that as 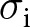 increases, individuals tend to engage more in both vertical and oblique learning at 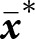 (i.e., 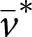 and 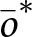 increase; see Fig. 3a). This is because high 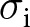 allows the population to accumulate substantial knowledge (see Fig. 3g), resulting in cultural exemplars possessing greater knowledge. Under high 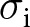, the increased efficiency of social learning then drives individuals to invest more resources in learning at 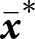 (i.e., high 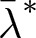; see Fig. 3d). This effect is especially pronounced in populations with low knowledge-production ability: stochasticity in learning can drive the evolution of great investment in learning (e.g., 
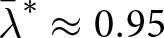
 when 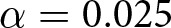 and 
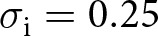
; see Fig. S.7), whereas in its absence, investment in learning would be low (e.g., 
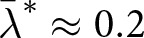
 when 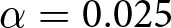 and 
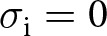
; see Fig. S.7). However, in populations with very low knowledge-production ability, individuals evolve to allocate all resources to fecundity and none to learning, so greater stochasticity in learning has no effect (e.g., 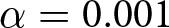; see Fig. S.7). The impact of 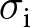 on learning traits at 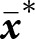 also weakens when the obsolescence rate is high, as rapid knowledge decay across generations limits knowledge accumulation (e.g., 
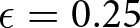
; Fig. S.6e). Although higher 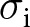 leads to investing more in learning at the expense of fecundity (i.e., higher 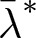), the resulting enhancement in population mean knowledge still leads to larger population sizes at 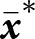 (see Fig. 3g).

Individual-based simulations, which notably relax the assumption of a Gaussian probability density of knowledge, confirm that greater stochasticity in individual learning promotes both increased time allocated to social learning and greater investment in learning at 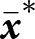 and leads to higher population mean knowledge and population size (see Fig. S.9). In addition, simulations that relax the assumption of non-stochastic social learning (i.e., allowing 
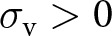
 or 
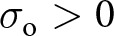
) reveal similar patterns under increased stochasticity in vertical and oblique learning (see Fig. S.10).

However, when adults knowledge significantly overlaps, an increase in 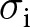 results in a greater allocation of time to vertical learning and a reduced allocation to oblique learning at 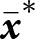 (e.g., increase in 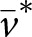 and decrease in 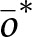 when 
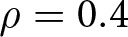
; see Fig. S.6f). In this regime, both parents and other adults provide access to similar knowledge units, so selection favours the most effective social learning mode for acquiring this shared knowledge. Under higher stochasticity in individual learning, the average knowledge of parents 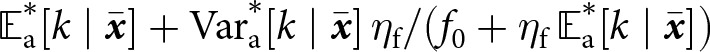 increases more than that of oblique exemplars 
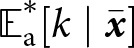
 (see Fig. S.8) since parents tend to have higher fertility and, consequently, above-average knowledge. As a result, when learning is more stochastic, offspring acquire more of the shared knowledge from their parents.

#### 3.2.4. Selection promotes learning from selected individuals

We now turn to the question of whom individuals should learn from when knowledge varies across the population. Specifically, we investigate which social learning exemplars are favoured by natural selection when knowledge improves survival and/or fecundity.

When knowledge enhances fecundity but does not affect survival (i.e., when 
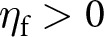
 and 
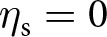
), increased stochasticity in individual learning (i.e., increased 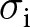) leads to allocating more time to vertical learning rather than oblique learning at 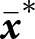 (i.e., a rise in 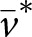 but not in 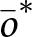; see Fig. 3b). In that case, stochasticity in individual learning leads to uncertainty in the knowledge held by potential social learning exemplars. Being a parent then acts as a cue for high fecundity and, therefore, greater knowledge. By contrast, when knowledge increases survival but does not affect fecundity (i.e., when 
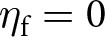
 and 
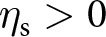
), increased stochasticity in individual learning (i.e., increased 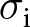) leads to allocating more time to both vertical and oblique learning at 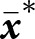 (i.e., a rise in 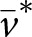 and 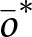; see Fig 3c). It follows that because adults who are available as social learning exemplars, whether during vertical or oblique learning, have survived until adulthood, their survival is a cue for sizable knowledge. In summary, when stochasticity in learning renders the knowledge of social learning exemplars unpredictable, natural selection favours learning from individuals who exhibit cues for possessing sizable knowledge (e.g., being a parent or having survived to adulthood).

#### 3.2.5. Selection promotes stochasticity in learning

The results so far show that stochasticity in individual learning, quantified by 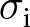, markedly affects both the cultural and evolutionary dynamics, and in perhaps counterintuitive ways. Yet, these results may not have traction if 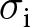 itself is selected away. Here, we show that learning stochasticity can in fact be favoured by selection. We relax the assumption of fixed 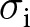 by introducing an additional evolving trait 
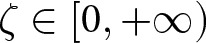
 that increases stochasticity (i.e., 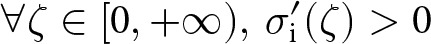). For instance, 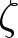 may be a behavioural tendency towards exploration during learning. We assume that learning is inherently stochastic, such that even in the absence of any trait promoting stochasticity (i.e., when 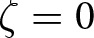), a small baseline level of stochasticity remains (i.e., 
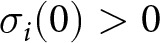
, but small). In Appendix C, we show that selection always favours the emergence of the trait 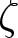. This is because in a lineage with traits 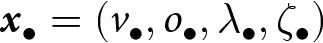, 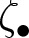 increases knowledge variance (see eq. (C.13)), thereby strengthening cultural selection and leading to higher expected knowledge (see eqs. (C.6) and (C.7)). This effect is further amplified under strong vertical transmission, as increases in expected knowledge driven by cultural selection accumulate across generations within the lineage (see eqs. (C.6) and (C.7)). These results thus confirm the potent role of 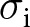 on both the cultural and evolutionary dynamics.

## 4. Discussion

We here first derived the dynamics of individual knowledge in a population using a stochastic model of learning that links generations through vertical and oblique transmission. We showed that as long as the learning process generates variability in knowledge, and knowledge is transmitted across generations, cultural selection, driven by differences in transmission linked to individual knowledge levels, will inevitably impact the dynamics of knowledge. By estimating the population mean of individual knowledge at equilibrium, our model shows that such cultural selection enhances cumulative knowledge. Those who, by chance, acquire greater knowledge are more likely to survive to adulthood, allowing them to interact with potential learners from the next generation, and to produce more offspring, thereby increasing opportunities for vertical transmission. As a result, individuals with higher knowledge are more likely to pass it on, thereby promoting knowledge accumulation across generations. Our results show that stochasticity in learning amplifies the action of natural selection by promoting variation in knowledge levels.

Our findings are consistent with previous theoretical work in which cultural selection arises because learners preferentially choose more knowledgeable exemplars (e.g., Henrich, 2004, Powell et al., 2009, Kobayashi and Aoki, 2012). In addition, our results highlight that cultural selection can operate even when learners cannot directly assess exemplar knowledge, as long as individuals with greater knowledge are still more likely to transmit, an effect also shown by Cavalli-Sforza and Feldman (1981). Thus, cultural selection is likely to contribute to the improvement of any form of adaptive knowledge that enhances fecundity or survival, even when it is difficult to identify which individuals possess greater knowledge. This may be particularly relevant for knowledge whose benefits are not directly observable because they may be delayed, probabilistic, or rely on hidden causal mechanisms, such as knowledge related to food processing, medicine, rituals, ecological knowledge, or institutional knowledge.

Stochasticity in learning has been extensively studied in reinforcement learning through the effects of random exploration of novel ‘actions’ (which can be thought of as cultural variants in our framework) on learning (Kaelbling et al., 1996, Ladosz et al., 2022, Hao et al., 2024). By increasing the chance of discovering high-reward actions, random exploration enhances the efficiency of selecting and retaining such actions, thereby improving reinforcement learning. While this role of stochasticity is well established, our results demonstrate that it can also enhance the efficiency of social learning, provided knowledge-biased transmission. In stationary environments, the advantages of random exploration in reinforcement learning generally decline as learners approach the optimal action (e.g., Tokic, 2010, Zhang et al., 2023). In contrast, in our model, greater stochasticity remains consistently advantageous because knowledge is unbounded and becomes obsolete due to environmental change. If we were instead to consider a cultural trait with an optimum (e.g., an ideal tool design or behaviour suited to a specific ecological challenge), then, much like in reinforcement learning, stochasticity would likely promote early cultural improvement by enabling the discovery of more adaptive variants, but slow convergence as the population’s traits approach their optimal value. For example, combining individual learning through trial-and-error with social transmission, trait variability across trials can speed convergence when the cultural trait is simple, but increases the distance from the optimum at equilibrium (Lehmann and Wakano, 2013).

Our results also show that, by enhancing cumulative knowledge, the cultural selection introduced by stochasticity in learning favours the evolution of extended social learning phases, as individuals can acquire substantial knowledge from exemplars. In addition, since social learning can provide a large amount of knowledge under these conditions, stochasticity in learning also promotes the evolution of more efficient learning, despite associated fecundity costs. This enables individuals to acquire a greater amount of knowledge during the social learning phase. Our findings may contribute to understanding how the mode of knowledge acquisition differs across knowledge types that vary in the extent of stochasticity in their production. For example, the production of opaque knowledge, such as adaptive taboos (Henrich and Henrich, 2010), adaptive supernatural beliefs (Lightner and Hagen, 2022), or food preparation methods that trigger complex chemical reactions (Beck, 1992), may be particularly stochastic, as their adaptive value is not readily apparent and it is difficult to intentionally produce knowledge that reliably enhances survival and fecundity. Due to this opacity, such knowledge likely became adaptive through a gradual accumulation of random improvements over time. As a result, individuals can acquire far more of this type of knowledge through social learning than they could by independently discovering it. Our results, therefore, predict that opaque knowledge would be primarily acquired socially. This could be the case for knowledge related to food preparation among the Aka Pygmies. Indeed, interviewed individuals reported that they had acquired all of their knowledge related to the preparation of koko, magnoc, and palm wine through social learning (Hewlett and Cavalli-Sforza, 1986). By contrast, other forms of knowledge, such as basic hunting techniques, navigating terrain, or learning to avoid predators, may rely on more transparent causal relationships. As a result, their acquisition may involve less stochasticity and is more likely to occur through individual learning. Nevertheless, stochasticity in knowledge production is likely to explain only part of the variation in modes of knowledge acquisition across knowledge types. Other factors, including the costs of producing knowledge, the need for motor skill practice, the ease of assessing success, and obsolescence, may also play important roles.

Our results reveal that whether knowledge affects fecundity or survival shapes the evolution of learning traits. For instance, when knowledge enhances only fecundity, natural selection favours individuals who mainly learn from their parents rather than from unrelated adults, consistent with the findings of McElreath and Strimling (2008). This is because, when stochasticity introduces variability in knowledge levels, parents tend to have above-average reproductive success and are therefore more likely to possess above-average knowledge. In contrast, randomly chosen adults tend to have average reproductive success and are not particularly likely to possess higher levels of knowledge. Therefore, individuals can acquire more knowledge by learning from their parents than from random adults. One interpretation is that parenthood serves as a cue for possessing knowledge that enhances fecundity, whereas simply being an adult provides no such indication. These findings suggest that knowledge enhancing fecundity should be preferentially acquired from parents. This pattern is supported by empirical evidence from the Aka Pygmies: interviewed individuals reported acquiring, on average, 85.6% of their knowledge about infant care from their parents, compared to 80.7% for knowledge across all domains (Hewlett and Cavalli-Sforza, 1986).

Our results rely on several assumptions that merit further discussion. In particular, we assume that knowledge affects survival and fecundity linearly. This assumption does not affect our conclusions regarding the impact of stochasticity in learning on cumulative knowledge and the evolution of learning traits. However, nonlinear fitness effects could alter knowledge dynamics and the evolutionary trajectories of learning traits, thereby quantitatively affecting our results. For instance, if knowledge yields increasing returns on survival and fecundity, individuals who possess above-average knowledge would gain greater advantages as knowledge accumulates in the population. This would amplify knowledge accumulation, since variation in knowledge would more strongly translate into differences in survival to adulthood and fecundity, thereby increasing cultural selection. As potential exemplars would carry a higher level of knowledge, selection would favour greater reliance on social learning. By contrast, if knowledge yields diminishing returns, we would expect the opposite outcome: knowledge accumulation would slow, and the evolutionary pressure for social learning would weaken.

We also make specific assumptions about the learning process. We assume that learning occurs sequentially through vertical, oblique, and individual phases. Although this is a simplification, it aligns with a general developmental pattern: social learning is more common early in life, while individual learning becomes more prevalent with age (Reader and Laland, 2001, Biesmeijer and Seeley, 2005, Noble et al., 2014, Carr et al., 2015). In humans, the sources of social learning also tend to shift, from primarily vertical transmission during childhood, to greater reliance on oblique transmission in later life stages (Hewlett et al., 2011, Demps et al., 2012, Garfield et al., 2016). In addition, we assume that individuals produce knowledge at a constant average rate, a simplification that allows us to focus on intergenerational knowledge accumulation without explicitly modelling the mechanistic processes underlying individual learning (but see Lehmann and Wakano, 2013, for a model with temporal cultural dynamics with mechanistic individual learning). Finally, we assume that the overlap in knowledge between two adults 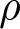 is constant. Allowing 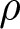 to emerge endogenously from the learning process could affect our results. Increased stochasticity in learning would reduce overlap in knowledge among adults, making oblique exemplars more likely to possess knowledge not held by parents and thereby promoting greater reliance on oblique learning.

Our study highlights the central role of stochasticity in learning in favouring cumulative knowledge and driving the evolution of learning behaviours. Such stochasticity may be widespread, not only because some level is inevitable, but also because our results show that selection favours increased stochasticity, as it increases the expected knowledge of lineage members when vertical transmission is non-negligible. However, fully understanding the evolution of traits underlying stochasticity in learning requires considering potential trade-offs; for example, a greater tendency to explore may be energetically costly or increase risk exposure. Another trade-off arises because exploratory behaviour may slow the rate of knowledge production, as random exploration is more likely to generate non-functional outcomes than functional ones. Incorporating this effect would require explicitly modelling the mechanistic processes underlying individual learning. These insights point to promising directions for future research on the evolutionary dynamics of traits impacting knowledge acquisition and their role in shaping cumulative cultural knowledge.

## Supporting information

10.1017/ehs.2026.10044.sm001 Maisonneuve and Lehmann supplementary material Maisonneuve and Lehmann supplementary material

## Data Availability

n/a

## Supplementary material

The supplementary material for this article can be found at https://doi.org/10.1017/ehs.2026.10044.

## Research transparency and reproducibility interest

All the codes used in this study are accessible at github.com/Ludovic-Maisonneuve/stochasticity_evolution_learning.
