# Supplementary material for: How does stochasticity in learning impact the accumulation of knowledge and the evolution of learning?
Source: Evol Hum Sci. 2026 Apr 6;8:e18. doi: 10.1017/ehs.2026.10044 (PMC13150785; doi:10.1017/ehs.2026.10044)
Supplement: Maisonneuve and Lehmann supplementary material [file S2513843X26100449sup001.pdf]

Appendix to "How does stochasticity in learning impact the accumulation of knowledge and the evolution of learning?"

Ludovic Maisonneuve[1,*], Laurent Lehmann[1]

1. Department of Ecology and Evolution, University of Lausanne, 1015 Lausanne, Switzerland

∗ ludovic.maisonneuve.2015@polytechnique.org.

Appendix A: Cultural dynamics and equilibrium

Here, we begin by highlighting the similarities and differences between our learning model and previous works (section A.1). We then characterize the outcome of the learning process described by eq. (2) in the absence of stochasticity in social learning (section A.2), and derive the recurrence equations for the expected knowledge and knowledge variance within an $\boldsymbol{x}_\bullet$-lineage using a Gaussian moment closure (section A.3). Next, we characterize the cultural equilibrium and develop a numerical procedure for computing the population mean knowledge and knowledge variance (section A.4).

A.1 Connection to previous models

Our learning model, described in the main text and formalized in eq. (2), builds on previous work by Kobayashi et al. (2016) and Maisonneuve et al. (2025), sharing some core assumptions while differing in key aspects. Following Kobayashi et al. (2016), we model stochasticity in both individual and social learning using using white noise from stochastic calculus (see eq. (2) in the main text and eq. (15) in the appendix of Kobayashi et al., 2016). As in Maisonneuve et al. (2025), we assume that learning occurs sequentially through vertical, oblique, and individual phases. A key difference between our model and that of Maisonneuve et al. (2025) lies in

the structure of oblique learning: while Maisonneuve et al. (2025) allows individuals to learn from all adults, we assume they learn from only one, reflecting the realistic constraint that offspring cannot interact with everyone. Learning from the entire adult population can inflate the available knowledge, especially under stochastic learning, where some individuals, by chance, accumulate significantly more knowledge than others.

A.2 Knowledge acquisition without stochasticity in social learning

In this section, we derive eq. (8) using stochastic calculus (e.g., Gardiner, 1985), which provides an expression for computing the realized knowledge acquired by a focal offspring at the end of its learning process, under the assumption that social learning occurs without stochasticity (i.e., $\sigma_\mathrm{v} = \sigma_\mathrm{o} = 0$). When we neglect stochasticity during social learning, the realized knowledge of a focal offspring at each age $a \in [0,1]$ with traits $\boldsymbol{x}_\bullet = (v_\bullet, o_\bullet, \lambda_\bullet)$, who learn from a parent with knowledge $k_{\mathrm{p}\bullet}$ and an oblique exemplar with knowledge $k_{\mathrm{a}\bullet}$, is a realization of the following stochastic differential equation

$$\frac{\mathrm{d}k_\bullet(a)}{\mathrm{d}a} = \begin{cases} \lambda_\bullet\,\beta_\mathrm{v}\,\left[(1-\epsilon)\,k_{\mathrm{p}\bullet} - k_\bullet(a)\right] & \text{for } a \in [0, v_\bullet) \\ \lambda_\bullet\,\beta_\mathrm{o}\,\left[(1-\epsilon)\,k_{\mathrm{a}\bullet} - \rho\,k_\bullet(v_\bullet) - \big(k_\bullet(a) - k_\bullet(v_\bullet)\big)\right] & \text{for } a \in [v_\bullet, v_\bullet + o_\bullet) \\ \lambda_\bullet\,\big(\alpha + \sigma_\mathrm{i}\,\eta(a)\big) & \text{for } a \in [v_\bullet + o_\bullet, 1], \end{cases} \tag{A.1}$$

with $k_\bullet(0) = 0$, which is obtained by substituting $\sigma_\mathrm{v} = \sigma_\mathrm{o} = 0$ into eq. (2).

For the final phase of learning, where the dynamics include a stochastic component, eq. (A.1) is interpreted in the Itô sense (Gardiner, 1985, Sec. 4.2, pp. 83–84), whereby

$$k_\bullet(a) = k_\bullet(v_\bullet + o_\bullet) + \int_{v_\bullet + o_\bullet}^{a} \lambda_\bullet\,\alpha\,\mathrm{d}s + \int_{v_\bullet + o_\bullet}^{a} \lambda_\bullet\,\sigma_\mathrm{i}\,\mathrm{d}B_s, \tag{A.2}$$

where B_s is the standard Brownian process, which is normally distributed with mean zero and variance s (Gardiner, 1985; Bass, 2011, and informally $\mathrm{d}B_s = \eta(s)\mathrm{d}s$).

Accordingly, the knowledge accumulated by the focal offspring at the end of the learning period is

$$k_\bullet(1) = k_\bullet(v_\bullet + o_\bullet) + \int_{v_\bullet + o_\bullet}^{1} \lambda_\bullet\,\alpha\,\mathrm{d}s + \int_{v_\bullet + o_\bullet}^{1} \lambda_\bullet\,\sigma_\mathrm{i}\,\mathrm{d}B_s, \tag{A.3}$$

which simplifies to

$$k_\bullet(1) = k_\bullet(v_\bullet + o_\bullet) + \lambda_\bullet\,\alpha\,(1 - v_\bullet - o_\bullet) + \chi_\mathrm{k}, \tag{A.4}$$

where

$$\chi_\mathrm{k} = \lambda_\bullet\,\sigma_\mathrm{i}\,B_{1-v_\bullet-o_\bullet} \tag{A.5}$$

captures the effect of stochastic fluctuations in individual learning. Since $B_{1-v_\bullet-o_\bullet}$ is the realization of a Gaussian random variable with mean 0 and variance $1-v_\bullet-o_\bullet$, it follows that χ_k can be obtained as a realization of a Gaussian variable with mean 0 and variance $\lambda_\bullet^2\,\sigma_\mathrm{i}^2\,(1-v_\bullet-o_\bullet)$.

Substituting the explicit expression for $k_\bullet(v_\bullet + o_\bullet)$, obtained by solving eq. (A.1) over the deterministic phases, into eq. (A.4) yields

$$\boxed{k_\bullet(1) = \overbrace{\mathcal{L}(\boldsymbol{x}_\bullet, k_{\mathrm{p}\bullet}, k_{\mathrm{a}\bullet})}^{\text{expected knowledge acquired}} + \overbrace{\chi_\mathrm{k}}^{\substack{\text{stochastic fluctuations}\\ \text{in knowledge acquired}}},} \tag{A.6}$$

where

$$\mathcal{L}(\boldsymbol{x}_\bullet, k_{\mathrm{p}\bullet}, k_{\mathrm{a}\bullet}) = \omega_\mathrm{v}(\boldsymbol{x}_\bullet)\,(1-\epsilon)\,k_{\mathrm{p}\bullet} + \omega_\mathrm{o}(\boldsymbol{x}_\bullet)\,\left[(1-\epsilon)\,k_{\mathrm{a}\bullet} - \rho\,\omega_\mathrm{v}(\boldsymbol{x}_\bullet)\,(1-\epsilon)\,k_{\mathrm{p}\bullet}\right] + \lambda_\bullet\,\alpha\,(1-v_\bullet-o_\bullet), \tag{A.7}$$

is the expected knowledge acquired. The terms $\omega_\mathrm{v}(\boldsymbol{x}_\bullet)$ and $\omega_\mathrm{o}(\boldsymbol{x}_\bullet)$, given by

$$\omega_\mathrm{v}(\boldsymbol{x}_\bullet) = 1 - \mathrm{e}^{-\lambda_\bullet\,\beta_\mathrm{v}\,v_\bullet} \quad \text{and} \quad \omega_\mathrm{o}(\boldsymbol{x}_\bullet) = 1 - \mathrm{e}^{-\lambda_\bullet\,\beta_\mathrm{o}\,o_\bullet}. \tag{A.8}$$

are the proportion of available knowledge at the start of the vertical and oblique learning phases, respectively, that is effectively transmitted to the focal offspring. Substituting the expression for $\mathcal{L}(\boldsymbol{x}_\bullet, k_{\mathrm{p}\bullet}, k_{\mathrm{a}\bullet})$ from eq. (A.7) into eq. (A.6) gives eq. (8) of the main text.

To simplify the notation in what follows, we rewrite eq. (A.7) as

$$\mathcal{L}(\boldsymbol{x}_\bullet, k_{\mathrm{p}\bullet}, k_{\mathrm{a}\bullet}) = h_\mathrm{vl}(\boldsymbol{x}_\bullet)\,k_{\mathrm{p}\bullet} + h_\mathrm{ol}(\boldsymbol{x}_\bullet)\,k_{\mathrm{a}\bullet} + p_\mathrm{il}(\boldsymbol{x}_\bullet), \tag{A.9}$$

where terms $h_\mathrm{vl}(\boldsymbol{x}_\bullet)$, $h_\mathrm{ol}(\boldsymbol{x}_\bullet)$ and $p_\mathrm{il}(\boldsymbol{x}_\bullet)$ are given by

$$h_\mathrm{vl}(\boldsymbol{x}_\bullet) = (1-\epsilon)\,\omega_\mathrm{v}(\boldsymbol{x}_\bullet)\,\left(1 - \rho\,\omega_\mathrm{o}(\boldsymbol{x}_\bullet)\right), \tag{A.10}$$

$$h_\mathrm{ol}(\boldsymbol{x}_\bullet) = (1-\epsilon)\,\omega_\mathrm{o}(\boldsymbol{x}_\bullet), \tag{A.11}$$

and

$$p_\mathrm{il}(\boldsymbol{x}_\bullet) = \lambda_\bullet\,\alpha\,(1-v_\bullet-o_\bullet). \tag{A.12}$$

A.3 Knowledge dynamics

In this section, we derive expressions for the change in the expected knowledge (section A.3.1) and knowledge variance (section A.3.2) in an $\boldsymbol{x}_\bullet$-lineage. Then we apply a Gaussian closure approximation to derive a closed dynamical system for tracking across generations the expected knowledge and knowledge variance (section A.3.3).

A.3.1 Expected knowledge dynamics

Here, we detail how we get eqs. (9) and (10) of the main text, which give the dynamics of the expected knowledge in an $\boldsymbol{x}_\bullet$-lineage. To this end, it is convenient to define

$$\mathbb{E}_{\mathrm{a},t+1}[u(k) \mid \boldsymbol{x}_\bullet] = \int u(k)\,\phi_{\mathrm{a},t+1}(k \mid \boldsymbol{x}_\bullet)\,\mathrm{d}k, \tag{A.13}$$

for any function of knowledge $u(k)$. This quantity denotes the expectation of $u(k)$ with respect to the probability density $\phi_{\mathrm{a},t+1}(k \mid \boldsymbol{x}_\bullet)$ that an adult from the $\boldsymbol{x}_\bullet$-lineage in generation $t+1$ possesses knowledge k. With this notation $\mathbb{E}_{\mathrm{a},t+1}[k \mid \boldsymbol{x}_\bullet]$ is the expected knowledge of a random adult from the $\boldsymbol{x}_\bullet$-lineage at generation $t+1$ (here and throughout the subscript ${}_{\mathrm{a},t}$ denotes quantities computed among adults of generation t for all $t \geq 1$; see fig. A.1).

By definition,

$$\mathbb{E}_{\mathrm{a},t+1}[k \mid \boldsymbol{x}_\bullet] = \int k\,\phi_{\mathrm{a},t+1}(k \mid \boldsymbol{x}_\bullet)\,\mathrm{d}k. \tag{A.14}$$

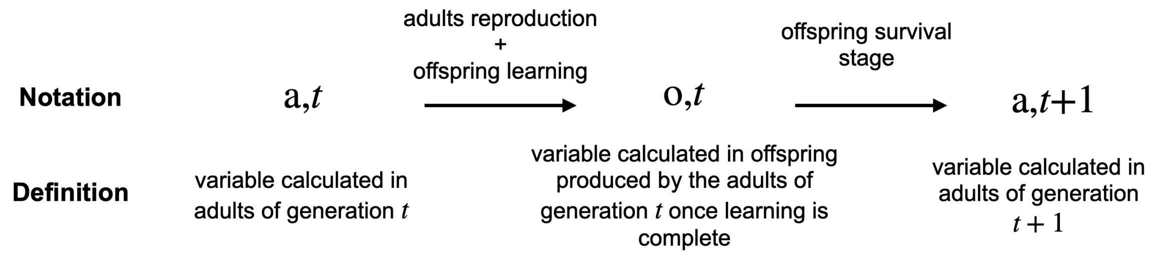

Figure A.1: **Notation specifying the stage in the life cycle and generation at which variables are calculated.**

The probability density that a random adult from the $\boldsymbol{x}_\bullet$-lineage of generation $t+1$ possesses knowledge k, $\phi_{\mathrm{a},t+1}(k \mid \boldsymbol{x}_\bullet)$, can be derived from the probability density that a random offspring produced by an adult from the $\boldsymbol{x}_\bullet$-lineage of generation t possesses knowledge k, $\phi_{\mathrm{o},t}(k \mid \boldsymbol{x}_\bullet)$ (here and throughout, the subscript ${}_{\mathrm{o},t}$ denotes quantities computed among an offspring produced by an adult of generation t after learning is complete and before the survival stage; see

fig. A.1), who will potentially become one of the adults of generation $t+1$. The probability density of knowledge of a random offspring is then weighted by the relative survival probability associated with each possible knowledge:

$$\forall k, \quad \phi_{\mathrm{a},t+1}(k \mid \boldsymbol{x}_\bullet) = \frac{s(k, n_{\mathrm{o},t})}{\mathbb{E}_{\mathrm{o},t}[s(k, n_{\mathrm{o},t}) \mid \boldsymbol{x}_\bullet]}\, \phi_{\mathrm{o},t}(k \mid \boldsymbol{x}_\bullet), \tag{A.15}$$

where $n_{\mathrm{o},t}$ is the number of offspring produced by adults of generation t and $s(k, n_{\mathrm{o},t})$ is the survival probability associated with traits $\boldsymbol{x}_\bullet$ and knowledge k. For any function of knowledge $u(k)$,

$$\mathbb{E}_{\mathrm{o},t}[u(k) \mid \boldsymbol{x}_\bullet] = \int u(k)\, \phi_{\mathrm{o},t}(k \mid \boldsymbol{x}_\bullet)\, \mathrm{d}k, \tag{A.16}$$

denotes the expectation of this function over the probability density of knowledge $\phi_{\mathrm{o},t}(k \mid \boldsymbol{x}_\bullet)$ of a random offspring produced by an adult of generation t. Here, $\mathbb{E}_{\mathrm{o},t}[s(k, n_{\mathrm{o},t}) \mid \boldsymbol{x}_\bullet]$ is the expected survival probability of an offspring produced by an adult of the $\boldsymbol{x}_\bullet$-lineage of generation t. Note that because $\phi_{\mathrm{a},t+1}(k \mid \boldsymbol{x}_\bullet)$ is a probability density of knowledge among surviving offspring within the $\boldsymbol{x}_\bullet$-lineage, the survival probability in eq. (A.15) is normalized by the expected survival probability in that lineage $\mathbb{E}_{\mathrm{o},t}[s(k, n_{\mathrm{o},t}) \mid \boldsymbol{x}_\bullet]$, not by the expected survival probability in the population.

Noting from eqs. (3) and (4) with $k_{\mathrm{o}\bullet} = k$ that $s(k, n_{\mathrm{o},t})$ depends linearly on k and using that $\int \phi_{\mathrm{o},t}(k \mid \boldsymbol{x}_\bullet)\, \mathrm{d}k = 1$ we have

$$\mathbb{E}_{\mathrm{o},t}[s(k, n_{\mathrm{o},t}) \mid \boldsymbol{x}_\bullet] = s(\mathbb{E}_{\mathrm{o},t}[k \mid \boldsymbol{x}_\bullet], n_{\mathrm{o},t}). \tag{A.17}$$

By substituting the expressions of $\phi_{\mathrm{a},t+1}(k \mid \boldsymbol{x}_\bullet)$ and $\mathbb{E}_{\mathrm{o},t}[s(k, n_{\mathrm{o},t}) \mid \boldsymbol{x}_\bullet]$ from eqs. (A.15) and (A.17) into eq. (A.14) we obtain

$$\mathbb{E}_{\mathrm{a},t+1}[k \mid \boldsymbol{x}_\bullet] = \int k\, \frac{s(k, n_{\mathrm{o},t})}{s(\mathbb{E}_{\mathrm{o},t}[k \mid \boldsymbol{x}_\bullet], n_{\mathrm{o},t})}\, \phi_{\mathrm{o},t}(k \mid \boldsymbol{x}_\bullet)\, \mathrm{d}k. \tag{A.18}$$

Next, we rewrite $s(k, n_{\mathrm{o},t})/s(\mathbb{E}_{\mathrm{o},t}[k \mid \boldsymbol{x}_\bullet], n_{\mathrm{o},t})$. Using the expression for $s(k, n_{\mathrm{o},t})$ and $s(\mathbb{E}_{\mathrm{o},t}[k \mid \boldsymbol{x}_\bullet], n_{\mathrm{o},t})$ from eq. (3) with $k_{\mathrm{o}\bullet} = k$, $n_{\mathrm{o}} = n_{\mathrm{o},t}$ and with $k_{\mathrm{o}\bullet} = \mathbb{E}_{\mathrm{o},t}[k \mid \boldsymbol{x}_\bullet]$, $n_{\mathrm{o}} = n_{\mathrm{o},t}$ we obtain

$$\frac{s(k, n_{\mathrm{o},t})}{s(\mathbb{E}_{\mathrm{o},t}[k \mid \boldsymbol{x}_\bullet], n_{\mathrm{o},t})} = \frac{\tilde{s}(k)}{\tilde{s}\big(\mathbb{E}_{\mathrm{o},t}[k \mid \boldsymbol{x}_\bullet]\big)}. \tag{A.19}$$

By substituting $k = \mathbb{E}_{\mathrm{o},t}[k \mid \boldsymbol{x}_\bullet] + \xi_{k_{\mathrm{o}\bullet},t}$, where $\xi_{k_{\mathrm{o}\bullet},t} = k - \mathbb{E}_{\mathrm{o},t}[k \mid \boldsymbol{x}_\bullet]$, into eq. (A.19) and noting from eq. (4) with $k_{\mathrm{o}\bullet} = k$ that $\tilde{s}(k)$ depends linearly on k with a linear coefficient η_s, we obtain

$$\frac{s(k, n_{\mathrm{o},t})}{s(\mathbb{E}_{\mathrm{o},t}[k \mid \boldsymbol{x}_\bullet], n_{\mathrm{o},t})} = 1 + \xi_{k_{\mathrm{o}\bullet},t}\, \frac{\eta_\mathrm{s}}{\tilde{s}\big(\mathbb{E}_{\mathrm{o},t}[k \mid \boldsymbol{x}_\bullet]\big)}. \tag{A.20}$$

Substituting $k = \mathbb{E}_{\mathrm{o},t}[k \mid \boldsymbol{x}_\bullet] + \xi_{k_{\mathrm{o}\bullet},t}$ and eq. (A.20) into eq. (A.18) gives

$$\mathbb{E}_{\mathrm{a},t+1}[k \mid \boldsymbol{x}_\bullet] = \mathbb{E}_{\mathrm{o},t}[k \mid \boldsymbol{x}_\bullet] + \frac{\eta_\mathrm{s}}{\tilde{s}\big(\mathbb{E}_{\mathrm{o},t}[k \mid \boldsymbol{x}_\bullet]\big)} \int \xi^2_{k_{\mathrm{o}\bullet},t}\, \phi_{\mathrm{o},t}(k \mid \boldsymbol{x}_\bullet)\, \mathrm{d}k. \tag{A.21}$$

Noting that the integral is the variance in the knowledge held by a random offspring produced by an adult from the $\boldsymbol{x}_\bullet$-lineage at generation t, denoted as $\mathrm{Var}_{\mathrm{o},t}[k \mid \boldsymbol{x}_\bullet]$, we obtain

$$\boxed{\mathbb{E}_{\mathrm{a},t+1}[k \mid \boldsymbol{x}_\bullet] = \mathbb{E}_{\mathrm{o},t}[k \mid \boldsymbol{x}_\bullet] + \frac{\mathrm{Var}_{\mathrm{o},t}[k \mid \boldsymbol{x}_\bullet]\, \eta_\mathrm{s}}{\tilde{s}\big(\mathbb{E}_{\mathrm{o},t}[k \mid \boldsymbol{x}_\bullet]\big)},} \tag{A.22}$$

which is eq. (10) from the main text.

Note that, in eq. (A.22), the term $\mathrm{Var}_{\mathrm{o},t}[k \mid \boldsymbol{x}_\bullet]\, \eta_\mathrm{s}/\tilde{s}\big(\mathbb{E}_{\mathrm{o},t}[k \mid \boldsymbol{x}_\bullet]\big)$ captures the effect of cultural selection arising from differential survival among lineage members, due to variation in knowledge.

To obtain the expected knowledge dynamics across one generation, we need to determine the expected knowledge of a random offspring produced by an adult of generation t, $\mathbb{E}_{\mathrm{o},t}[k \mid \boldsymbol{x}_\bullet]$. By definition,

$$\mathbb{E}_{\mathrm{o},t}[k \mid \boldsymbol{x}_\bullet] = \int k\, \phi_{\mathrm{o},t}(k \mid \boldsymbol{x}_\bullet)\, \mathrm{d}k. \tag{A.23}$$

For each offspring, the knowledge acquired depends on the knowledge of both the parent and the oblique exemplar, as well as on the outcome of the stochasticity in the learning process. By integrating over the probability density of parental knowledge, the probability density of knowledge among potential oblique exemplars, and the probability density of stochastic learning outcomes, we obtain

$$\mathbb{E}_{\mathrm{o},t}[k \mid \boldsymbol{x}_\bullet] = \iiint k\, \nu(k \mid \boldsymbol{x}_\bullet, k'', k')\, \phi_{\mathrm{a},t}(k' \mid \bar{\boldsymbol{x}})\, \frac{f(\boldsymbol{x}_\bullet, k'')}{\mathbb{E}_{\mathrm{a},t}[f(\boldsymbol{x}_\bullet, k) \mid \boldsymbol{x}_\bullet]}\, \phi_{\mathrm{a},t}(k'' \mid \boldsymbol{x}_\bullet)\, \mathrm{d}k\, \mathrm{d}k'\, \mathrm{d}k'', \tag{A.24}$$

where $\mathbb{E}_{\mathrm{a},t}[f(\boldsymbol{x}_\bullet, k) \mid \boldsymbol{x}_\bullet]$ is the mean fecundity of adults belonging to the $\boldsymbol{x}_\bullet$-lineage at generation

t and is equal to

$$\mathbb{E}_{\mathrm{a},t}[f(\boldsymbol{x}_\bullet, k) \mid \boldsymbol{x}_\bullet] = \int f(\boldsymbol{x}_\bullet, k)\, \phi_{\mathrm{a},t}(k \mid \boldsymbol{x}_\bullet)\, \mathrm{d}k = f\big(\boldsymbol{x}_\bullet, \mathbb{E}_{\mathrm{a},t}[k \mid \boldsymbol{x}_\bullet]\big). \tag{A.25}$$

The integral over k'' encompasses all possible knowledge value of parents, weighted by the probability density $f(\boldsymbol{x}_\bullet, k'')/\mathbb{E}_{\mathrm{a},t}[f(\boldsymbol{x}_\bullet, k) \mid \boldsymbol{x}_\bullet]\, \phi_{\mathrm{a},t}(k'' \mid \boldsymbol{x}_\bullet)$ that a parent has knowledge k''. Each parent's contribution depends on its fecundity relative to the lineage's mean fecundity, $f(\boldsymbol{x}_\bullet, k'')/\mathbb{E}_{\mathrm{a},t}[f(\boldsymbol{x}_\bullet, k) \mid \boldsymbol{x}_\bullet]$, because lineage members with higher fecundity are more likely to transmit their traits and knowledge.

The integral over k' encompasses all possible knowledge values of oblique exemplars in generation t, weighted by the probability density $\phi_{\mathrm{a},t}(k' \mid \bar{\boldsymbol{x}})$ that an oblique exemplar has knowledge k'. Since the variances of population traits are small, we approximate the population as being quasi-monomorphic for the mean traits $\bar{\boldsymbol{x}}$, and thus assume that offspring acquire knowledge obliquely from adults with traits $\bar{\boldsymbol{x}}$.

Finally, the integral over k encompasses all possible outcomes of the learning process, weighted by the probability density $\nu(k \mid \boldsymbol{x}_\bullet, k'', k')$ of the offspring's knowledge k at the end of the learning process, given parental knowledge k'' and oblique learning examplar knowledge k'.

From eq. (A.6) with $k_\bullet(1) = k$, we have that $k = \mathcal{L}(\boldsymbol{x}_\bullet, k'', k') + \chi_{\mathrm{k}}$ where $\mathcal{L}(\boldsymbol{x}_\bullet, k'', k')$ is deterministic and χ_{k} is a realization a Gaussian random variable with mean 0 and variance $\lambda_\bullet^2\, \sigma_{\mathrm{i}}^2\, (1 - v_\bullet - o_\bullet)$. Since k is the sum of a deterministic term $\mathcal{L}(\boldsymbol{x}_\bullet, k'', k')$ and a random fluctuation χ_{k}, its probability density has the same shape as that of χ_{k}, but centered around $\mathcal{L}(\boldsymbol{x}_\bullet, k'', k')$ instead of 0. This means the probability density of k is obtained by shifting the density of χ_{k} horizontally so that its mean aligns with $\mathcal{L}(\boldsymbol{x}_\bullet, k'', k')$

$$\nu(k \mid \boldsymbol{x}_\bullet, k'', k') = \nu_{\chi_{\mathrm{k}}}\big(k - \mathcal{L}(\boldsymbol{x}_\bullet, k'', k') \mid \boldsymbol{x}_\bullet\big), \tag{A.26}$$

where $\nu_{\chi_{\mathrm{k}}}$ is a Gaussian probability density function with mean 0 and variance $\lambda_\bullet^2\, \sigma_{\mathrm{i}}^2\, (1 - v_\bullet - o_\bullet)$. We obtain eq. (A.26) by noting that $k - \mathcal{L}(\boldsymbol{x}_\bullet, k'', k') = \chi_{\mathrm{k}}$, so the probability that k takes a particular value is the same as the probability that χ_{k} takes the value $k - \mathcal{L}(\boldsymbol{x}_\bullet, k'', k')$.

By substituting the expressions of $\mathbb{E}_{\mathrm{a},t}[f(\boldsymbol{x}_\bullet, k) \mid \boldsymbol{x}_\bullet]$ and $\nu(k \mid \boldsymbol{x}_\bullet, k'', k')$ from eqs. (A.25) and (A.26) into eq. (A.24) and by performing a change of variable $\chi_{\mathrm{k}} = k - \mathcal{L}(\boldsymbol{x}_\bullet, k'', k')$ we obtain

$$\mathbb{E}_{\mathrm{o},t}[k \mid \boldsymbol{x}_\bullet] = \iiint \Bigg[\big(\mathcal{L}(\boldsymbol{x}_\bullet, k'', k') + \chi_\mathrm{k}\big) \\ \times \nu_{\chi_\mathrm{k}}(\chi_\mathrm{k} \mid \boldsymbol{x}_\bullet)\, \phi_{\mathrm{a},t}(k' \mid \bar{\boldsymbol{x}})\, \frac{f(\boldsymbol{x}_\bullet, k'')}{f\big(\boldsymbol{x}_\bullet, \mathbb{E}_{\mathrm{a},t}[k \mid \boldsymbol{x}_\bullet]\big)}\, \phi_{\mathrm{a},t}(k'' \mid \boldsymbol{x}_\bullet)\Bigg] \mathrm{d}\chi_\mathrm{k}\, \mathrm{d}k'\, \mathrm{d}k''. \quad (A.27)$$

Equation (A.27) can be rearranged as follows

$$\mathbb{E}_{\mathrm{o},t}[k \mid \boldsymbol{x}_\bullet] = \iint \Bigg[\bigg(\mathcal{L}(\boldsymbol{x}_\bullet, k'', k') + \int \chi_\mathrm{k}\, \nu_{\chi_\mathrm{k}}(\chi_\mathrm{k} \mid \boldsymbol{x}_\bullet)\, \mathrm{d}\chi_\mathrm{k}\bigg) \\ \times \phi_{\mathrm{a},t}(k' \mid \bar{\boldsymbol{x}})\, \frac{f(\boldsymbol{x}_\bullet, k'')}{f\big(\boldsymbol{x}_\bullet, \mathbb{E}_{\mathrm{a},t}[k \mid \boldsymbol{x}_\bullet]\big)}\, \phi_{\mathrm{a},t}(k'' \mid \boldsymbol{x}_\bullet)\Bigg] \mathrm{d}k'\, \mathrm{d}k'', \quad (A.28)$$

and, because χ_k is centered at 0 it simplifies to

$$\mathbb{E}_{\mathrm{o},t}[k \mid \boldsymbol{x}_\bullet] = \iint \mathcal{L}(\boldsymbol{x}_\bullet, k'', k')\, \phi_{\mathrm{a},t}(k' \mid \bar{\boldsymbol{x}})\, \frac{f(\boldsymbol{x}_\bullet, k'')}{f\big(\boldsymbol{x}_\bullet, \mathbb{E}_{\mathrm{a},t}[k \mid \boldsymbol{x}_\bullet]\big)}\, \phi_{\mathrm{a},t}(k'' \mid \boldsymbol{x}_\bullet)\, \mathrm{d}k'\, \mathrm{d}k''. \quad (A.29)$$

Next, we expand $f(\boldsymbol{x}_\bullet, k'')/f\big(\boldsymbol{x}_\bullet, \mathbb{E}_{\mathrm{a},t}[k \mid \boldsymbol{x}_\bullet]\big)$. By substituting $k'' = \mathbb{E}_{\mathrm{a},t}[k \mid \boldsymbol{x}_\bullet] + \xi_{k_{\mathrm{p}\bullet},t}$, where $\xi_{k_{\mathrm{p}\bullet},t} = k'' - \mathbb{E}_{\mathrm{a},t}[k \mid \boldsymbol{x}_\bullet]$, and noting from eq. (1) that $f(\boldsymbol{x}_\bullet, k'')$ depends linearly on k'', we obtain

$$\frac{f(\boldsymbol{x}_\bullet, k'')}{f\big(\boldsymbol{x}_\bullet, \mathbb{E}_{\mathrm{a},t}[k \mid \boldsymbol{x}_\bullet]\big)} = 1 + \xi_{k_{\mathrm{p}\bullet},t}\, \frac{(1-\lambda_\bullet)^\theta\, \eta_\mathrm{f}}{f\big(\boldsymbol{x}_\bullet, \mathbb{E}_{\mathrm{a},t}[k \mid \boldsymbol{x}_\bullet]\big)}. \quad (A.30)$$

By substituting the expression of $f\big(\boldsymbol{x}_\bullet, \mathbb{E}_{\mathrm{a},t}[k \mid \boldsymbol{x}_\bullet]\big)$ from eq. (1) with $k'' = \mathbb{E}_{\mathrm{a},t}[k \mid \boldsymbol{x}_\bullet]$ into the right-hand side of eq. (A.30) we obtain

$$\frac{f(\boldsymbol{x}_\bullet, k'')}{f\big(\boldsymbol{x}_\bullet, \mathbb{E}_{\mathrm{a},t}[k \mid \boldsymbol{x}_\bullet]\big)} = 1 + \xi_{k_{\mathrm{p}\bullet},t}\, \frac{\eta_\mathrm{f}}{f_0 + \eta_\mathrm{f}\, \mathbb{E}_{\mathrm{a},t}[k \mid \boldsymbol{x}_\bullet]}. \quad (A.31)$$

We now rewrite $\mathcal{L}(\boldsymbol{x}_\bullet, k'', k')$. Because $\mathcal{L}(\boldsymbol{x}_\bullet, k'', k')$ is linear on k'' and k' with coefficient of linearity $h_\mathrm{vl}(\boldsymbol{x}_\bullet)$ and $h_\mathrm{ol}(\boldsymbol{x}_\bullet)$ (see eq. (A.9)), by substituting $k_{\mathrm{p}\bullet} = \mathbb{E}_{\mathrm{a},t}[k \mid \boldsymbol{x}_\bullet] + \xi_{k_{\mathrm{p}\bullet},t}$ and $k' = \mathbb{E}_{\mathrm{a},t}[k \mid \bar{\boldsymbol{x}}] + \xi_{k_{\mathrm{a}\bullet},t}$, where $\xi_{k_{\mathrm{a}\bullet},t} = \big(k' - \mathbb{E}_{\mathrm{a},t}[k \mid \bar{\boldsymbol{x}}]\big)$, we obtain

$$\mathcal{L}(\boldsymbol{x}_\bullet, k'', k') = \mathcal{L}\big(\boldsymbol{x}_\bullet, \mathbb{E}_{\mathrm{a},t}[k \mid \boldsymbol{x}_\bullet], \mathbb{E}_{\mathrm{a},t}[k \mid \bar{\boldsymbol{x}}]\big) + \xi_{k_{\mathrm{p}\bullet},t}\, h_\mathrm{vl}(\boldsymbol{x}_\bullet) + \xi_{k_{\mathrm{a}\bullet},t}\, h_\mathrm{ol}(\boldsymbol{x}_\bullet). \quad (A.32)$$

Substituting eqs. (A.31) and (A.32) into eq. (A.29), and noting that $\xi_{k_{\mathrm{p}\bullet},t}$ and $\xi_{k_{\mathrm{a}\bullet},t}$ are centered

around zero and are independent, we obtain

$$\mathbb{E}_{\mathrm{o},t}[k \mid \boldsymbol{x}_\bullet] = \mathcal{L}\big(\boldsymbol{x}_\bullet, \mathbb{E}_{\mathrm{a},t}[k \mid \boldsymbol{x}_\bullet], \mathbb{E}_{\mathrm{a},t}[k \mid \bar{\boldsymbol{x}}]\big) + h_{\mathrm{vl}}(\boldsymbol{x}_\bullet)\, \frac{\eta_{\mathrm{f}}}{f_0 + \eta_{\mathrm{f}}\, \mathbb{E}_{\mathrm{a},t}[k \mid \boldsymbol{x}_\bullet]} \int \xi^2_{k_{\mathrm{p}\bullet},t}\, \phi_{\mathrm{a},t}(k'' \mid \boldsymbol{x}_\bullet)\, \mathrm{d}k''. \tag{A.33}$$

Noting that the integral is the variance in the knowledge held by a random adult from the $\boldsymbol{x}_\bullet$-lineage at generation t, denoted as $\mathrm{Var}_{\mathrm{a},t}[k \mid \boldsymbol{x}_\bullet]$, we obtain

$$\boxed{\mathbb{E}_{\mathrm{o},t}[k \mid \boldsymbol{x}_\bullet] = \mathcal{L}\big(\boldsymbol{x}_\bullet, \mathbb{E}_{\mathrm{a},t}[k \mid \boldsymbol{x}_\bullet], \mathbb{E}_{\mathrm{a},t}[k \mid \bar{\boldsymbol{x}}]\big) + \frac{h_{\mathrm{vl}}(\boldsymbol{x}_\bullet)\, \mathrm{Var}_{\mathrm{a},t}[k \mid \boldsymbol{x}_\bullet]\, \eta_{\mathrm{f}}}{f_0 + \eta_{\mathrm{f}}\, \mathbb{E}_{\mathrm{a},t}[k \mid \boldsymbol{x}_\bullet]}.} \tag{A.34}$$

By substituting the expression of $\mathcal{L}\big(\boldsymbol{x}_\bullet, \mathbb{E}_{\mathrm{a},t}[k \mid \boldsymbol{x}_\bullet], \mathbb{E}_{\mathrm{a},t}[k \mid \bar{\boldsymbol{x}}]\big)$ from eq. (A.9) with $k_{\mathrm{p}\bullet} = \mathbb{E}_{\mathrm{a},t}[k \mid \boldsymbol{x}_\bullet]$ and $k_{\mathrm{a}\bullet} = \mathbb{E}_{\mathrm{a},t}[k \mid \bar{\boldsymbol{x}}]$ gives eq. (9) from the main text.

Note that, in eq. (A.34), the term $h_{\mathrm{vl}}(\boldsymbol{x}_\bullet)\, \mathrm{Var}_{\mathrm{a},t}[k \mid \boldsymbol{x}_\bullet]\, \eta_{\mathrm{f}} / \big(f_0 + \eta_{\mathrm{f}}\, \mathbb{E}_{\mathrm{a},t}[k \mid \boldsymbol{x}_\bullet]\big)$ captures the effect of cultural selection arising from differential fecundity among lineage members, due to variation in knowledge.

Equations (A.22) and (A.34) give the dynamics of the expected knowledge within the $\boldsymbol{x}_\bullet$-lineage.

A.3.2 Knowledge variance dynamics

Equations (A.22) and (A.34) enable us to track changes in expected knowledge within a lineage across a single generation. However, to iterate these equations also requires tracking the variance in the knowledge held by a lineage member simultaneously. In this section, we derive the expression of the dynamics of the variance in the knowledge held by a random adult within the $\boldsymbol{x}_\bullet$-lineage.

By definition, the variance in the knowledge held by a random adult from the $\boldsymbol{x}_\bullet$-lineage at generation $t+1$, $\mathrm{Var}_{\mathrm{a},t+1}[k \mid \boldsymbol{x}_\bullet]$, is given by

$$\mathrm{Var}_{\mathrm{a},t+1}[k \mid \boldsymbol{x}_\bullet] = \int \big(k - \mathbb{E}_{\mathrm{a},t+1}[k \mid \boldsymbol{x}_\bullet]\big)^2\, \phi_{\mathrm{a},t+1}(k \mid \boldsymbol{x}_\bullet)\, \mathrm{d}k. \tag{A.35}$$

Using $k - \mathbb{E}_{\mathrm{a},t+1}[k \mid \boldsymbol{x}_\bullet] = \xi_{k_{\mathrm{o}\bullet},t} - \big(\mathbb{E}_{\mathrm{a},t+1}[k \mid \boldsymbol{x}_\bullet] - \mathbb{E}_{\mathrm{o},t}[k \mid \boldsymbol{x}_\bullet]\big)$ and $\int \xi_{k_{\mathrm{o}\bullet},t}\, \phi_{\mathrm{a},t+1}(k \mid \boldsymbol{x}_\bullet)\, \mathrm{d}k = \mathbb{E}_{\mathrm{a},t+1}[k \mid \boldsymbol{x}_\bullet] - \mathbb{E}_{\mathrm{o},t}[k \mid \boldsymbol{x}_\bullet]$ we can expand eq. (A.35) as follows

$$\mathrm{Var}_{\mathrm{a},t+1}[k \mid \boldsymbol{x}_\bullet] = \int \xi^2_{k_{\mathrm{o}\bullet},t}\, \phi_{\mathrm{a},t+1}(k \mid \boldsymbol{x}_\bullet)\, \mathrm{d}k - \left(\mathbb{E}_{\mathrm{a},t+1}[k \mid \boldsymbol{x}_\bullet] - \mathbb{E}_{\mathrm{o},t}[k \mid \boldsymbol{x}_\bullet]\right)^2. \tag{A.36}$$

By substituting the expression of $\phi_{\mathrm{a},t+1}(k \mid \boldsymbol{x}_\bullet)$ from eq. (A.15) into eq. (A.36), we obtain

$$\mathrm{Var}_{\mathrm{a},t+1}[k \mid \boldsymbol{x}_\bullet] = \int \xi_{k_{\mathrm{o}\bullet},t}^2 \frac{s(k, n_{\mathrm{o},t})}{\mathbb{E}_{\mathrm{o},t}[s(k, n_{\mathrm{o},t}) \mid \boldsymbol{x}_\bullet]} \phi_{\mathrm{o},t}(k \mid \boldsymbol{x}_\bullet) \,\mathrm{d}k - \left(\mathbb{E}_{\mathrm{a},t+1}[k \mid \boldsymbol{x}_\bullet] - \mathbb{E}_{\mathrm{o},t}[k \mid \boldsymbol{x}_\bullet]\right)^2 . \tag{A.37}$$

Substituting the expression of $s(k, n_{\mathrm{o},t})/\mathbb{E}_{\mathrm{o},t}[s(k, n_{\mathrm{o},t}) \mid \boldsymbol{x}_\bullet]$ from eq. (A.20) (with $s(\mathbb{E}_{\mathrm{o},t}[k \mid \boldsymbol{x}_\bullet], n_{\mathrm{o},t}) = \mathbb{E}_{\mathrm{o},t}[s(k, n_{\mathrm{o},t}) \mid \boldsymbol{x}_\bullet])$ into eq. (A.37) we find

$$\mathrm{Var}_{\mathrm{a},t+1}[k \mid \boldsymbol{x}_\bullet] = \int \xi_{k_{\mathrm{o}\bullet},t}^2 \left(1 + \xi_{k_{\mathrm{o}\bullet},t} \frac{\eta_{\mathrm{s}}}{\tilde{s}\big(\mathbb{E}_{\mathrm{o},t}[k \mid \boldsymbol{x}_\bullet]\big)}\right) \phi_{\mathrm{o},t}(k \mid \boldsymbol{x}_\bullet) \,\mathrm{d}k - \left(\mathbb{E}_{\mathrm{a},t+1}[k \mid \boldsymbol{x}_\bullet] - \mathbb{E}_{\mathrm{o},t}[k \mid \boldsymbol{x}_\bullet]\right)^2 . \tag{A.38}$$

By expanding the integral in eq. (A.38) we find

$$\begin{aligned} V_{\mathrm{a},t+1} = \int \xi_{k_{\mathrm{o}\bullet},t}^2 \, \phi_{\mathrm{o},t}(k \mid \boldsymbol{x}_\bullet) \,\mathrm{d}k + \frac{\eta_{\mathrm{s}}}{\tilde{s}\big(\mathbb{E}_{\mathrm{o},t}[k \mid \boldsymbol{x}_\bullet]\big)} \int \xi_{k_{\mathrm{o}\bullet},t}^3 \, \phi_{\mathrm{o},t}(k \mid \boldsymbol{x}_\bullet) \,\mathrm{d}k \\ - \left(\mathbb{E}_{\mathrm{a},t+1}[k \mid \boldsymbol{x}_\bullet] - \mathbb{E}_{\mathrm{o},t}[k \mid \boldsymbol{x}_\bullet]\right)^2 . \end{aligned} \tag{A.39}$$

Noting that

$$\int \big(k - \mathbb{E}_{\mathrm{o},t}[k \mid \boldsymbol{x}_\bullet]\big)^2 \phi_{\mathrm{o},t}(k \mid \boldsymbol{x}_\bullet) \,\mathrm{d}k = \mathrm{Var}_{\mathrm{o},t}[k \mid \boldsymbol{x}_\bullet], \tag{A.40}$$

and

$$\int \big(k - \mathbb{E}_{\mathrm{o},t}[k \mid \boldsymbol{x}_\bullet]\big)^3 \phi_{\mathrm{o},t}(k \mid \boldsymbol{x}_\bullet) \,\mathrm{d}k = \mathrm{Skew}_{\mathrm{o},t}[k|\boldsymbol{x}_\bullet], \tag{A.41}$$

where $\mathrm{Skew}_{\mathrm{o},t}[k|\boldsymbol{x}_\bullet]$ is the skewness in the knowledge held by a random offspring produced by an adult from the $\boldsymbol{x}_\bullet$-lineage at generation t, and by substituting the expression for $\mathbb{E}_{\mathrm{a},t+1}[k \mid \boldsymbol{x}_\bullet]$ from eq. (A.22) into eq. (A.39), we obtain

$$\boxed{\mathrm{Var}_{\mathrm{a},t+1}[k \mid \boldsymbol{x}_\bullet] = \mathrm{Var}_{\mathrm{o},t}[k \mid \boldsymbol{x}_\bullet] + \frac{\mathrm{Skew}_{\mathrm{o},t}[k|\boldsymbol{x}_\bullet]\,\eta_{\mathrm{s}}}{\tilde{s}\big(\mathbb{E}_{\mathrm{o}}^*[k \mid \boldsymbol{x}_\bullet]\big)} - \left(\frac{\mathrm{Var}_{\mathrm{o},t}[k \mid \boldsymbol{x}_\bullet]\,\eta_{\mathrm{s}}}{\tilde{s}\big(\mathbb{E}_{\mathrm{o}}^*[k \mid \boldsymbol{x}_\bullet]\big)}\right)^2 .} \tag{A.42}$$

We now compute the variance in the knowledge held by a random offspring produced by an adult from the $\boldsymbol{x}_\bullet$-lineage at generation t, $\mathrm{Var}_{\mathrm{o},t}[k \mid \boldsymbol{x}_\bullet]$. The variance $\mathrm{Var}_{\mathrm{o},t}[k \mid \boldsymbol{x}_\bullet]$ can be obtained by integrating over the probability density of parental knowledge, the probability density of knowledge among potential oblique exemplars, and the probability density of all stochastic learning outcomes to obtain

$$\mathrm{Var}_{\mathrm{o},t}[k \mid \boldsymbol{x}_\bullet] = \iiint \Bigg[\big(k - \mathbb{E}_{\mathrm{o},t}[k \mid \boldsymbol{x}_\bullet]\big)^2 \times \nu(k \mid \boldsymbol{x}_\bullet, k'', k')\, \phi_{\mathrm{a},t}(k' \mid \bar{\boldsymbol{x}})\, \frac{f(\boldsymbol{x}_\bullet, k'')}{\mathbb{E}_{\mathrm{a},t}[f(\boldsymbol{x}_\bullet, k) \mid \boldsymbol{x}_\bullet]}\, \phi_{\mathrm{a},t}(k'' \mid \boldsymbol{x}_\bullet) \Bigg] \mathrm{d}k\, \mathrm{d}k'\, \mathrm{d}k''. \quad \text{(A.43)}$$

By substituting the expressions of $\mathbb{E}_{\mathrm{a},t}[f(\boldsymbol{x}_\bullet, k) \mid \boldsymbol{x}_\bullet]$ and $\nu(k \mid \boldsymbol{x}_\bullet, k'', k')$ from eqs. (A.25) and (A.26) into eq. (A.43) and by performing a change of variable $\chi_{\mathrm{k}} = k - \mathcal{L}(\boldsymbol{x}_\bullet, k'', k')$ we obtain

$$\mathrm{Var}_{\mathrm{o},t}[k \mid \boldsymbol{x}_\bullet] = \iiint \Bigg[\big(\mathcal{L}(\boldsymbol{x}_\bullet, k'', k') + \chi_{\mathrm{k}} - \mathbb{E}_{\mathrm{o},t}[k \mid \boldsymbol{x}_\bullet]\big)^2 \times \nu_{\chi_{\mathrm{k}}}(\chi_{\mathrm{k}} \mid \boldsymbol{x}_\bullet)\, \phi_{\mathrm{a},t}(k' \mid \bar{\boldsymbol{x}})\, \frac{f(\boldsymbol{x}_\bullet, k'')}{f\big(\boldsymbol{x}_\bullet, \mathbb{E}_{\mathrm{a},t}[k \mid \boldsymbol{x}_\bullet]\big)}\, \phi_{\mathrm{a},t}(k'' \mid \boldsymbol{x}_\bullet) \Bigg] \mathrm{d}\chi_{\mathrm{k}}\, \mathrm{d}k'\, \mathrm{d}k''. \quad \text{(A.44)}$$

By substituting the expression of $f(\boldsymbol{x}_\bullet, k'')/f\big(\boldsymbol{x}_\bullet, \mathbb{E}_{\mathrm{a},t}[k \mid \boldsymbol{x}_\bullet]\big)$ and $\mathcal{L}(\boldsymbol{x}_\bullet, k'', k')$ from eqs. (A.31) and (A.32) into eq. (A.44) we obtain

$$\begin{aligned}
&\mathrm{Var}_{\mathrm{o},t}[k \mid \boldsymbol{x}_\bullet] \\
&= \iiint \Bigg[\left(\mathcal{L}\left(\boldsymbol{x}_\bullet, \mathbb{E}_{\mathrm{a},t}[k \mid \boldsymbol{x}_\bullet], \mathbb{E}_{\mathrm{a},t}[k \mid \bar{\boldsymbol{x}}]\right) - \mathbb{E}_{\mathrm{o},t}[k \mid \boldsymbol{x}_\bullet] + \xi_{k_{\mathrm{p}\bullet},t}\, h_{\mathrm{vl}}(\boldsymbol{x}_\bullet) + \xi_{k_{\mathrm{a}\bullet},t}\, h_{\mathrm{ol}}(\boldsymbol{x}_\bullet) + \chi_{\mathrm{k}}\right)^2 \\
&\times \left(1 + \xi_{k_{\mathrm{p}\bullet},t}\, \frac{\eta_{\mathrm{f}}}{f_0 + \eta_{\mathrm{f}}\, \mathbb{E}_{\mathrm{a},t}[k \mid \boldsymbol{x}_\bullet]}\right) \nu_{\chi_{\mathrm{k}}}(\chi_{\mathrm{k}} \mid \boldsymbol{x}_\bullet)\, \phi_{\mathrm{a},t}(k' \mid \bar{\boldsymbol{x}})\, \phi_{\mathrm{a},t}(k'' \mid \boldsymbol{x}_\bullet) \Bigg] \mathrm{d}\chi_{\mathrm{k}}\, \mathrm{d}k'\, \mathrm{d}k''. \quad \text{(A.45)}
\end{aligned}$$

Expanding the square in eq. (A.45), and noting that χ_{k} is centered at zero, we get

$$\begin{aligned}
&\mathrm{Var}_{\mathrm{o},t}[k \mid \boldsymbol{x}_\bullet] = V_{\mathrm{l}}(\boldsymbol{x}_\bullet) \\
&+ \iiint \Bigg[\Big(\mathcal{L}\big(\boldsymbol{x}_\bullet, \mathbb{E}_{\mathrm{a},t}[k \mid \boldsymbol{x}_\bullet], \mathbb{E}_{\mathrm{a},t}[k \mid \bar{\boldsymbol{x}}]\big) - \mathbb{E}_{\mathrm{o},t}[k \mid \boldsymbol{x}_\bullet] + \xi_{k_{\mathrm{p}\bullet},t}\, h_{\mathrm{vl}}(\boldsymbol{x}_\bullet) + \xi_{k_{\mathrm{a}\bullet},t}\, h_{\mathrm{ol}}(\boldsymbol{x}_\bullet)\Big)^2 \\
&\times \left(1 + \xi_{k_{\mathrm{p}\bullet},t}\, \frac{\eta_{\mathrm{f}}}{f_0 + \eta_{\mathrm{f}}\, \mathbb{E}_{\mathrm{a},t}[k \mid \boldsymbol{x}_\bullet]}\right) \nu_{\chi_{\mathrm{k}}}(\chi_{\mathrm{k}} \mid \boldsymbol{x}_\bullet)\, \phi_{\mathrm{a},t}(k' \mid \bar{\boldsymbol{x}})\, \phi_{\mathrm{a},t}(k'' \mid \boldsymbol{x}_\bullet) \Bigg] \mathrm{d}\chi_{\mathrm{k}}\, \mathrm{d}k'\, \mathrm{d}k'', \quad \text{(A.46)}
\end{aligned}$$

where

$$V_{\mathrm{l}}(\boldsymbol{x}_\bullet) = \int \nu_{\chi_{\mathrm{k}}}(\chi_{\mathrm{k}} \mid \boldsymbol{x}_\bullet)\, \chi_{\mathrm{k}}^2\, \mathrm{d}\chi_{\mathrm{k}} = \lambda_\bullet^2\, \sigma_{\mathrm{i}}^2\, (1 - v_\bullet - o_\bullet). \quad \text{(A.47)}$$

Expanding the integral in eq. (A.46) and noting that $\xi_{k_{\mathrm{p}\bullet},t}$ and $\xi_{k_{\mathrm{a}\bullet},t}$ are centered around zero and independent, we find

$$\mathrm{Var}_{\mathrm{o},t}[k \mid \boldsymbol{x}_\bullet] = V_\mathrm{l}(\boldsymbol{x}_\bullet) + h_\mathrm{vl}(\boldsymbol{x}_\bullet)^2 \int \xi^2_{k_{\mathrm{p}\bullet},t}\, \phi_{\mathrm{a},t}(k'' \mid \boldsymbol{x}_\bullet)\,\mathrm{d}k'' + h_\mathrm{ol}(\boldsymbol{x}_\bullet)^2 \int \xi^2_{k_{\mathrm{a}\bullet},t}\, \phi_{\mathrm{a},t}(k' \mid \bar{\boldsymbol{x}})\,\mathrm{d}k'$$
$$+\big(\mathcal{L}\big(\boldsymbol{x}_\bullet, \mathbb{E}_{\mathrm{a},t}[k \mid \boldsymbol{x}_\bullet], \mathbb{E}_{\mathrm{a},t}[k \mid \bar{\boldsymbol{x}}]\big) - \mathbb{E}_{\mathrm{o},t}[k \mid \boldsymbol{x}_\bullet]\big)^2 + h_\mathrm{vl}(\boldsymbol{x}_\bullet)^2 \frac{\eta_\mathrm{f}}{f_0 + \eta_\mathrm{f}\,\mathbb{E}_{\mathrm{a},t}[k \mid \boldsymbol{x}_\bullet]} \int \xi^3_{k_{\mathrm{p}\bullet},t}\, \phi_{\mathrm{a},t}(k'' \mid \boldsymbol{x}_\bullet)\,\mathrm{d}k''$$
$$+2\,\big(\mathcal{L}\big(\boldsymbol{x}_\bullet, \mathbb{E}_{\mathrm{a},t}[k \mid \boldsymbol{x}_\bullet], \mathbb{E}_{\mathrm{a},t}[k \mid \bar{\boldsymbol{x}}]\big) - \mathbb{E}_{\mathrm{o},t}[k \mid \boldsymbol{x}_\bullet]\big)\; h_\mathrm{vl}(\boldsymbol{x}_\bullet) \frac{\eta_\mathrm{f}}{f_0 + \eta_\mathrm{f}\,\mathbb{E}_{\mathrm{a},t}[k \mid \boldsymbol{x}_\bullet]} \int \xi^2_{k_{\mathrm{p}\bullet},t}\, \phi_{\mathrm{a},t}(k'' \mid \boldsymbol{x}_\bullet)\,\mathrm{d}k''. \tag{A.48}$$

Noting that

$$\int \xi^2_{k_{\mathrm{p}\bullet},t}\, \phi_{\mathrm{a},t}(k'' \mid \boldsymbol{x}_\bullet)\,\mathrm{d}k'' = \mathrm{Var}_{\mathrm{a},t}[k \mid \boldsymbol{x}_\bullet], \tag{A.49}$$

$$\int \xi^2_{k_{\mathrm{a}\bullet},t}\, \phi_{\mathrm{a},t}(k' \mid \bar{\boldsymbol{x}})\,\mathrm{d}k' = \mathrm{Var}_{\mathrm{a},t}[k \mid \bar{\boldsymbol{x}}], \tag{A.50}$$

and

$$\int \xi^3_{k_{\mathrm{p}\bullet},t}\, \phi_{\mathrm{a},t}(k'' \mid \boldsymbol{x}_\bullet)\,\mathrm{d}k'' = \mathrm{Skew}_{\mathrm{a},t}[k|\boldsymbol{x}_\bullet], \tag{A.51}$$

where $\mathrm{Skew}_{\mathrm{a},t}[k|\boldsymbol{x}_\bullet]$ is the skewness in the knowledge held by a random adult from the $\boldsymbol{x}_\bullet$-lineage at generation t and by substituting the expression for $\mathbb{E}_{\mathrm{o},t}[k \mid \boldsymbol{x}_\bullet]$ from eq. (A.34) into eq. (A.48) we obtain

$$\boxed{\begin{aligned}\mathrm{Var}_{\mathrm{o},t}[k \mid \boldsymbol{x}_\bullet] = V_\mathrm{l}(\boldsymbol{x}_\bullet) + h_\mathrm{vl}(\boldsymbol{x}_\bullet)^2\,\mathrm{Var}_{\mathrm{a},t}[k \mid \boldsymbol{x}_\bullet] + h_\mathrm{ol}(\boldsymbol{x}_\bullet)^2\,\mathrm{Var}_{\mathrm{a},t}[k \mid \bar{\boldsymbol{x}}] + \frac{h_\mathrm{vl}(\boldsymbol{x}_\bullet)^2\,\mathrm{Skew}_{\mathrm{a},t}[k|\boldsymbol{x}_\bullet]\,\eta_\mathrm{f}}{f_0 + \eta_\mathrm{f}\,\mathbb{E}_{\mathrm{a},t}[k \mid \boldsymbol{x}_\bullet]}\\ - \left(\frac{h_\mathrm{vl}(\boldsymbol{x}_\bullet)\,\mathrm{Var}_{\mathrm{a},t}[k \mid \boldsymbol{x}_\bullet]\,\eta_\mathrm{f}}{f_0 + \eta_\mathrm{f}\,\mathbb{E}_{\mathrm{a},t}[k \mid \boldsymbol{x}_\bullet]}\right)^2.\end{aligned}} \tag{A.52}$$

Equations (A.42) and (A.52) give the dynamics of knowledge variance.

A.3.3 Gaussian closure approximation

Equations (A.22), (A.34), (A.42), and (A.52) describe the dynamics of the expected knowledge and knowledge variance in a lineage. However, tracking the expected knowledge and knowledge variance requires tracking knowledge skewness, which in turn necessitates tracking fourth-order moments of knowledge probability density. Since the dynamics of each moment depend on higher-order moments, tracking the cultural dynamics entails following the dynamics of the infinite sequence of moments. To circumvent this issue, we apply a Gaussian closure approximation, assuming that the probability density of knowledge within a lineage can be approximated by a Gaussian probability density. This assumption allows us to track the evolu-

tion of the probability density of knowledge across generations by tracking only its mean and variance.

Under the Gaussian closure approximation, the knowledge skewness is zero. By substituting $\mathrm{Skew}_{\mathrm{a},t}[k|\boldsymbol{x}_\bullet] = \mathrm{Skew}_{\mathrm{o},t}[k|\boldsymbol{x}_\bullet] = 0$ into the system of equations formed by eqs. (A.22), (A.34), (A.42) and (A.52) we obtain

$$\begin{cases} \mathbb{E}_{\mathrm{a},t+1}[k \mid \boldsymbol{x}_\bullet] = \mathbb{E}_{\mathrm{o},t}[k \mid \boldsymbol{x}_\bullet] + \frac{\mathrm{Var}_{\mathrm{o},t}[k|\boldsymbol{x}_\bullet]\,\eta_\mathrm{s}}{\tilde{s}\left(\mathbb{E}^*_\mathrm{o}[k|\boldsymbol{x}_\bullet]\right)} \\ \mathbb{E}_{\mathrm{o},t}[k \mid \boldsymbol{x}_\bullet] = \mathcal{L}\left(\boldsymbol{x}_\bullet, \mathbb{E}_{\mathrm{a},t}[k \mid \boldsymbol{x}_\bullet], \mathbb{E}_{\mathrm{a},t}[k \mid \bar{\boldsymbol{x}}]\right) + \frac{h_{\mathrm{vl}}(\boldsymbol{x}_\bullet)\,\mathrm{Var}_{\mathrm{a},t}[k|\boldsymbol{x}_\bullet]\,\eta_\mathrm{f}}{f_0+\eta_\mathrm{f}\,\mathbb{E}_{\mathrm{a},t}[k|\boldsymbol{x}_\bullet]} \\ \mathrm{Var}_{\mathrm{a},t+1}[k \mid \boldsymbol{x}_\bullet] = \mathrm{Var}_{\mathrm{o},t}[k \mid \boldsymbol{x}_\bullet] - \left(\frac{\mathrm{Var}_{\mathrm{o},t}[k|\boldsymbol{x}_\bullet]\,\eta_\mathrm{s}}{\tilde{s}\left(\mathbb{E}^*_\mathrm{o}[k|\boldsymbol{x}_\bullet]\right)}\right)^2 \\ \mathrm{Var}_{\mathrm{o},t}[k \mid \boldsymbol{x}_\bullet] = V_\mathrm{l}(\boldsymbol{x}_\bullet) + h_{\mathrm{vl}}(\boldsymbol{x}_\bullet)^2\,\mathrm{Var}_{\mathrm{a},t}[k \mid \boldsymbol{x}_\bullet] + h_{\mathrm{ol}}(\boldsymbol{x}_\bullet)^2\,\mathrm{Var}_{\mathrm{a},t}[k \mid \bar{\boldsymbol{x}}] - \left(\frac{h_{\mathrm{vl}}(\boldsymbol{x}_\bullet)\,\mathrm{Var}_{\mathrm{a},t}[k|\boldsymbol{x}_\bullet]\,\eta_\mathrm{f}}{f_0+\eta_\mathrm{f}\,\mathbb{E}_{\mathrm{a},t}[k|\boldsymbol{x}_\bullet]}\right)^2, \end{cases} \tag{A.53}$$

which characterized the dynamics of the expected knowledge and knowledge variance of the $\boldsymbol{x}_\bullet$-lineage member.

A.4 Probability density of knowledge at cultural equilibrium

In this section, we first characterize the equilibrium conditions for the expected knowledge and knowledge variance at cultural equilibrium (sections A.4.1 and A.4.2). Next, we demonstrate that in the absence of stochasticity in learning, there is no knowledge variance at the cultural equilibrium (section A.4.3). Finally, we describe the numerical method used to estimate the equilibrium values of mean knowledge and knowledge variance in the population (section A.4.4).

A.4.1 Characterization of the expected knowledge at cultural equilibrium

Here, we derive eq. (11) from the main text, which specifies the condition satisfied by the expected adults knowledge $\mathbb{E}^*_\mathrm{a}[k \mid \boldsymbol{x}_\bullet]$ at equilibrium (here and hereafter the notation * denotes a variable at equilibrium).

At equilibrium, the expected knowledge and the variance in knowledge for randomly chosen adults and offspring remain unchanged across generations. By substituting $\mathbb{E}_{\mathrm{a},t+1}[k \mid \boldsymbol{x}_\bullet] = \mathbb{E}_{\mathrm{a},t}[k \mid \boldsymbol{x}_\bullet] = \mathbb{E}^*_\mathrm{a}[k \mid \boldsymbol{x}_\bullet]$, $\mathbb{E}_{\mathrm{o},t}[k \mid \boldsymbol{x}_\bullet] = \mathbb{E}^*_\mathrm{o}[k \mid \boldsymbol{x}_\bullet]$, $\mathrm{Var}_{\mathrm{a},t+1}[k \mid \boldsymbol{x}_\bullet] = \mathrm{Var}_{\mathrm{a},t}[k \mid \boldsymbol{x}_\bullet] = \mathrm{Var}^*_\mathrm{a}[k \mid \boldsymbol{x}_\bullet]$ and $\mathrm{Var}_{\mathrm{o},t}[k \mid \boldsymbol{x}_\bullet] = \mathrm{Var}^*_\mathrm{o}[k \mid \boldsymbol{x}_\bullet]$ into the dynamical system eq. (A.53), we obtain

the following conditions that these equilibrium values must satisfy

$$\begin{cases} \mathbb{E}^*_{\mathrm{a}}[k \mid \boldsymbol{x}_\bullet] = \mathbb{E}^*_{\mathrm{o}}[k \mid \boldsymbol{x}_\bullet] + \frac{\mathrm{Var}^*_{\mathrm{o}}[k|\boldsymbol{x}_\bullet]\,\eta_{\mathrm{s}}}{\tilde{s}\left(\mathbb{E}^*_{\mathrm{o}}[k|\boldsymbol{x}_\bullet]\right)} \\ \mathbb{E}^*_{\mathrm{o}}[k \mid \boldsymbol{x}_\bullet] = \mathcal{L}\left(\boldsymbol{x}_\bullet, \mathbb{E}^*_{\mathrm{a}}[k \mid \boldsymbol{x}_\bullet], \mathbb{E}^*_{\mathrm{a}}[k \mid \bar{\boldsymbol{x}}]\right) + \frac{h_{\mathrm{vl}}(\boldsymbol{x}_\bullet)\,\mathrm{Var}^*_{\mathrm{a}}[k|\boldsymbol{x}_\bullet]\,\eta_{\mathrm{f}}}{f_0+\eta_{\mathrm{f}}\,\mathbb{E}^*_{\mathrm{a}}[k|\boldsymbol{x}_\bullet]} \\ \mathrm{Var}^*_{\mathrm{a}}[k \mid \boldsymbol{x}_\bullet] = \mathrm{Var}^*_{\mathrm{o}}[k \mid \boldsymbol{x}_\bullet] - \left(\frac{\mathrm{Var}^*_{\mathrm{o}}[k|\boldsymbol{x}_\bullet]\,\eta_{\mathrm{s}}}{\tilde{s}\left(\mathbb{E}^*_{\mathrm{o}}[k|\boldsymbol{x}_\bullet]\right)}\right)^2 \\ \mathrm{Var}^*_{\mathrm{o}}[k \mid \boldsymbol{x}_\bullet] = V_{\mathrm{l}}(\boldsymbol{x}_\bullet) + h_{\mathrm{vl}}(\boldsymbol{x}_\bullet)^2\,\mathrm{Var}^*_{\mathrm{a}}[k \mid \boldsymbol{x}_\bullet] + h_{\mathrm{ol}}(\boldsymbol{x}_\bullet)^2\,\mathrm{Var}^*_{\mathrm{a}}[k \mid \bar{\boldsymbol{x}}] - \left(\frac{h_{\mathrm{vl}}(\boldsymbol{x}_\bullet)\,\mathrm{Var}^*_{\mathrm{a}}[k|\boldsymbol{x}_\bullet]\,\eta_{\mathrm{f}}}{f_0+\eta_{\mathrm{f}}\,\mathbb{E}^*_{\mathrm{a}}[k|\boldsymbol{x}_\bullet]}\right)^2, \end{cases} \tag{A.54}$$

where $\mathbb{E}^*_{\mathrm{a}}[k \mid \bar{\boldsymbol{x}}]$ and $\mathrm{Var}^*_{\mathrm{a}}[k \mid \bar{\boldsymbol{x}}]$ are the population's mean and variance of adult knowledge, which verifies eq. (A.54) with $\boldsymbol{x}_\bullet = \bar{\boldsymbol{x}}$.

By replacing $\mathcal{L}\left(\boldsymbol{x}_\bullet, \mathbb{E}^*_{\mathrm{a}}[k \mid \boldsymbol{x}_\bullet], \mathbb{E}^*_{\mathrm{a}}[k \mid \bar{\boldsymbol{x}}]\right)$ with its expression from eq. (A.9) (with $k'' = \mathbb{E}^*_{\mathrm{a}}[k \mid \boldsymbol{x}_\bullet]$ and $k_{\mathrm{a}\bullet} = \mathbb{E}^*_{\mathrm{a}}[k \mid \bar{\boldsymbol{x}}]$) in the second line of eq. (A.54), we obtain

$$\mathbb{E}^*_{\mathrm{o}}[k \mid \boldsymbol{x}_\bullet] = h_{\mathrm{vl}}(\boldsymbol{x}_\bullet)\,\mathbb{E}^*_{\mathrm{a}}[k \mid \boldsymbol{x}_\bullet] + h_{\mathrm{ol}}(\boldsymbol{x}_\bullet)\,\mathbb{E}^*_{\mathrm{a}}[k \mid \bar{\boldsymbol{x}}] + p_{\mathrm{il}}(\boldsymbol{x}_\bullet) + \frac{h_{\mathrm{vl}}(\boldsymbol{x}_\bullet)\,\mathrm{Var}^*_{\mathrm{a}}[k \mid \boldsymbol{x}_\bullet]\,\eta_{\mathrm{f}}}{f_0 + \eta_{\mathrm{f}}\,\mathbb{E}^*_{\mathrm{a}}[k \mid \boldsymbol{x}_\bullet]}. \tag{A.55}$$

By substituting the expression for $\mathbb{E}^*_{\mathrm{o}}[k \mid \boldsymbol{x}_\bullet]$ from eq. (A.55) into the first line of eq. (A.54), we obtain

$$\mathbb{E}^*_{\mathrm{a}}[k \mid \boldsymbol{x}_\bullet] = h_{\mathrm{vl}}(\boldsymbol{x}_\bullet)\,\mathbb{E}^*_{\mathrm{a}}[k \mid \boldsymbol{x}_\bullet] + h_{\mathrm{ol}}(\boldsymbol{x}_\bullet)\,\mathbb{E}^*_{\mathrm{a}}[k \mid \bar{\boldsymbol{x}}] + p_{\mathrm{il}}(\boldsymbol{x}_\bullet) + \frac{h_{\mathrm{vl}}(\boldsymbol{x}_\bullet)\,\mathrm{Var}^*_{\mathrm{a}}[k \mid \boldsymbol{x}_\bullet]\,\eta_{\mathrm{f}}}{f_0 + \eta_{\mathrm{f}}\,\mathbb{E}^*_{\mathrm{a}}[k \mid \boldsymbol{x}_\bullet]} + \frac{\mathrm{Var}^*_{\mathrm{o}}[k \mid \boldsymbol{x}_\bullet]\,\eta_{\mathrm{s}}}{\tilde{s}\left(\mathbb{E}^*_{\mathrm{o}}[k \mid \boldsymbol{x}_\bullet]\right)}. \tag{A.56}$$

This can be rewritten as

$$\boxed{\mathbb{E}^*_{\mathrm{a}}[k \mid \boldsymbol{x}_\bullet] = \frac{p_{\mathrm{il}}(\boldsymbol{x}_\bullet) + h_{\mathrm{ol}}(\boldsymbol{x}_\bullet)\,\mathbb{E}^*_{\mathrm{a}}[k \mid \bar{\boldsymbol{x}}] + \frac{h_{\mathrm{vl}}(\boldsymbol{x}_\bullet)\,\mathrm{Var}^*_{\mathrm{a}}[k|\boldsymbol{x}_\bullet]\,\eta_{\mathrm{f}}}{f_0+\eta_{\mathrm{f}}\,\mathbb{E}^*_{\mathrm{a}}[k|\boldsymbol{x}_\bullet]} + \frac{\mathrm{Var}^*_{\mathrm{o}}[k|\boldsymbol{x}_\bullet]\,\eta_{\mathrm{s}}}{\tilde{s}\left(\mathbb{E}^*_{\mathrm{o}}[k|\boldsymbol{x}_\bullet]\right)}}{1 - h_{\mathrm{vl}}(\boldsymbol{x}_\bullet)}.} \tag{A.57}$$

Equation (A.57) is eq. (11) from the main text.

A.4.2 Characterization of the knowledge variance at cultural equilibrium

Here, we derive eq. (12) from the main text, which specifies the condition satisfied by the variance in the knowledge held by a random adult $\mathrm{Var}^*_{\mathrm{a}}[k \mid \boldsymbol{x}_\bullet]$ at equilibrium. By substituting the expression for $\mathrm{Var}^*_{\mathrm{o}}[k \mid \boldsymbol{x}_\bullet]$ from the fourth line of eq. (A.54) into the third line of the same

equation, we obtain

$$\mathrm{Var}_{\mathrm{a}}^{*}[k \mid \boldsymbol{x}_{\bullet}] = V_{\mathrm{l}}(\boldsymbol{x}_{\bullet}) + h_{\mathrm{vl}}(\boldsymbol{x}_{\bullet})^2 \,\mathrm{Var}_{\mathrm{a}}^{*}[k \mid \boldsymbol{x}_{\bullet}] + h_{\mathrm{ol}}(\boldsymbol{x}_{\bullet})^2 \,\mathrm{Var}_{\mathrm{a}}^{*}[k \mid \bar{\boldsymbol{x}}] - \left(\frac{h_{\mathrm{vl}}(\boldsymbol{x}_{\bullet}) \,\mathrm{Var}_{\mathrm{a}}^{*}[k \mid \boldsymbol{x}_{\bullet}]\, \eta_{\mathrm{f}}}{f_0 + \eta_{\mathrm{f}}\, \mathbb{E}_{\mathrm{a}}^{*}[k \mid \boldsymbol{x}_{\bullet}]}\right)^2 - \left(\frac{\mathrm{Var}_{\mathrm{o}}^{*}[k \mid \boldsymbol{x}_{\bullet}]\, \eta_{\mathrm{s}}}{\tilde{s}\big(\mathbb{E}_{\mathrm{o}}^{*}[k \mid \boldsymbol{x}_{\bullet}]\big)}\right)^2, \tag{A.58}$$

which can be rearranged as

$$\boxed{\mathrm{Var}_{\mathrm{a}}^{*}[k \mid \boldsymbol{x}_{\bullet}] = \frac{V_{\mathrm{l}}(\boldsymbol{x}_{\bullet}) + h_{\mathrm{ol}}(\boldsymbol{x}_{\bullet})^2 \,\mathrm{Var}_{\mathrm{a}}^{*}[k \mid \bar{\boldsymbol{x}}] - \left(\frac{h_{\mathrm{vl}}(\boldsymbol{x}_{\bullet}) \,\mathrm{Var}_{\mathrm{a}}^{*}[k|\boldsymbol{x}_{\bullet}]\, \eta_{\mathrm{f}}}{f_0 + \eta_{\mathrm{f}}\, \mathbb{E}_{\mathrm{a}}^{*}[k|\boldsymbol{x}_{\bullet}]}\right)^2 - \left(\frac{\mathrm{Var}_{\mathrm{o}}^{*}[k|\boldsymbol{x}_{\bullet}]\, \eta_{\mathrm{s}}}{\tilde{s}\big(\mathbb{E}_{\mathrm{o}}^{*}[k|\boldsymbol{x}_{\bullet}]\big)}\right)^2}{1 - h_{\mathrm{vl}}(\boldsymbol{x}_{\bullet})^2}.} \tag{A.59}$$

Substituting the expression for $V_{\mathrm{l}}(\boldsymbol{x}_{\bullet})$ from eq. (A.47) into eq. (A.59) gives eq. (12) from the main text.

A.4.3 Knowledge variance in the absence of stochasticity in learning

Here, we show that if individual learning is deterministic (i.e., $\sigma_{\mathrm{i}} = 0$), the equilibrium variance of adult knowledge is zero (i.e., $\mathrm{Var}_{\mathrm{a}}^{*}[k \mid \boldsymbol{x}_{\bullet}] = 0$).

We assume that, initially, all individuals have no knowledge, so that the knowledge variance is null: $V_0(\bar{\boldsymbol{x}}) = 0$. We aim to prove that this remains true for all generations t, i.e., $\mathrm{Var}_{\mathrm{a},t}[k \mid \bar{\boldsymbol{x}}] = 0$ for all t. Using mathematical induction, this is equivalent to showing that $\mathrm{Var}_{\mathrm{a},t}[k \mid \bar{\boldsymbol{x}}] = 0$ implies $\mathrm{Var}_{\mathrm{a},t+1}[k \mid \bar{\boldsymbol{x}}] = 0$.

Assume that $\mathrm{Var}_{\mathrm{a},t}[k \mid \bar{\boldsymbol{x}}] = 0$. Substituting $\sigma_{\mathrm{i}} = 0$ into eq. (A.47), we obtain $V_{\mathrm{l}}(\boldsymbol{x}_{\bullet}) = 0$. Then, substituting both $\mathrm{Var}_{\mathrm{a},t}[k \mid \bar{\boldsymbol{x}}] = 0$ and $V_{\mathrm{l}}(\boldsymbol{x}_{\bullet}) = 0$ into the fourth line of eq. (A.53), we find that $\mathrm{Var}_{\mathrm{o},t}[k \mid \boldsymbol{x}_{\bullet}] = 0$. Finally, substituting $\mathrm{Var}_{\mathrm{o},t}[k \mid \boldsymbol{x}_{\bullet}] = 0$ into the third line of eq. (A.53) yields $\mathrm{Var}_{\mathrm{a},t+1}[k \mid \bar{\boldsymbol{x}}] = 0$. This completes the inductive step and shows that $\mathrm{Var}_{\mathrm{a},t}[k \mid \bar{\boldsymbol{x}}] = 0$ for all t, which implies that at equilibrium, $\mathrm{Var}_{\mathrm{a}}^{*}[k \mid \boldsymbol{x}_{\bullet}] = 0$. This concludes the proof.

A.4.4 Numerical estimation of population mean and variance in knowledge

To numerically determine the mean and variance of knowledge within the population at cultural equilibrium, $\mathbb{E}_{\mathrm{a}}^{*}[k \mid \bar{\boldsymbol{x}}]$ and $\mathrm{Var}_{\mathrm{a}}^{*}[k \mid \bar{\boldsymbol{x}}]$, we iterated the dynamics described by eq. (A.53) with $\boldsymbol{x}_{\bullet} = \bar{\boldsymbol{x}}$ across generations, starting from the initial conditions $\bar{k}_{\mathrm{a},1}(\bar{\boldsymbol{x}}) = V_{\mathrm{a},1}(\bar{\boldsymbol{x}}) = 0$. The

expressions for $\tilde{s}(\bar{k}_{\mathrm{o},t}(\bar{\boldsymbol{x}}))$, $f(\bar{\boldsymbol{x}}, \mathbb{E}_{\mathrm{a},t}[k \mid \bar{\boldsymbol{x}}])$, $\mathcal{L}(\bar{\boldsymbol{x}}, \mathbb{E}_{\mathrm{a},t}[k \mid \bar{\boldsymbol{x}}], \mathbb{E}_{\mathrm{a},t}[k \mid \bar{\boldsymbol{x}}])$, $h_{\mathrm{vl}}(\bar{\boldsymbol{x}})$, $h_{\mathrm{ol}}(\bar{\boldsymbol{x}})$, $p_{\mathrm{il}}(\bar{\boldsymbol{x}})$ and $V_{\mathrm{l}}(\boldsymbol{x}_\bullet)$ are given by eqs. (4) (with $\boldsymbol{x}_\bullet = \bar{\boldsymbol{x}}$ and $k_{\mathrm{o}\bullet} = \bar{k}_{\mathrm{o},t}(\bar{\boldsymbol{x}})$), (1) (with $\boldsymbol{x}_\bullet = \bar{\boldsymbol{x}}$ and $k_{\mathrm{p}\bullet} = \mathbb{E}_{\mathrm{a},t}[k \mid \bar{\boldsymbol{x}}]$), (A.9) (with $\boldsymbol{x}_\bullet = \bar{\boldsymbol{x}}$ and $k_{\mathrm{p}\bullet} = k_{\mathrm{a}\bullet} = \mathbb{E}_{\mathrm{a},t}[k \mid \bar{\boldsymbol{x}}]$), (A.10) (with $\boldsymbol{x}_\bullet = \bar{\boldsymbol{x}}$), (A.11) (with $\boldsymbol{x}_\bullet = \bar{\boldsymbol{x}}$), (A.12) (with $\boldsymbol{x}_\bullet = \bar{\boldsymbol{x}}$) and (A.47) (with $\boldsymbol{x}_\bullet = \bar{\boldsymbol{x}}$) respectively, treating $\bar{\boldsymbol{x}}$ as a parameter. We iterate this dynamics until

$$\sqrt{\left(\mathbb{E}_{\mathrm{a},t+1}[k \mid \bar{\boldsymbol{x}}] - \mathbb{E}_{\mathrm{a},t}[k \mid \bar{\boldsymbol{x}}]\right)^2 + \left(\mathrm{Var}_{\mathrm{a},t+1}[k \mid \bar{\boldsymbol{x}}] - \mathrm{Var}_{\mathrm{a},t}[k \mid \bar{\boldsymbol{x}}]\right)^2} < 10^{-6}.$$

Appendix B: Evolutionary dynamics

We here derive eq. (5) from the main text. We then derive eq. (14) from the main text, which establishes the conditions that $\nabla\mathbb{E}^*_{\mathrm{a}}[k \mid \boldsymbol{x}_\bullet]|_{\boldsymbol{x}_\bullet=\bar{\boldsymbol{x}}}$ and $\nabla\mathbb{E}^*_{\mathrm{o}}[k \mid \boldsymbol{x}_\bullet]|_{\boldsymbol{x}_\bullet=\bar{\boldsymbol{x}}}$ satisfy (section B.2). Lastly, we detail the numerical procedure for computing the values of mean traits favored by selection (section B.3).

B.1 Lineage fitness

We now explain how we obtained eq. (5), which provides an expression for the lineage fitness $W(\boldsymbol{x}_\bullet, \bar{\boldsymbol{x}})$ of an $\boldsymbol{x}_\bullet$-lineage introduced into a population with mean trait values $\bar{\boldsymbol{x}}$. Lineage fitness is here defined as that quantity, which determines whether a single mutation resulting in trait expression $\boldsymbol{x}_\bullet$ will go extinct with certainty or has a positive probability of persisting in a population with average trait $\bar{\boldsymbol{x}}$, i.e., the mutation's lineage goes extinct with probability one if and only if $W(\boldsymbol{x}_\bullet, \bar{\boldsymbol{x}}) \leq 1$. This perspective envisions the process of spread of the mutant lineage $\boldsymbol{x}_\bullet$ as a branching process (Harris, 1963) and has been used before to understand invasion fitness in class-structured populations when interactions occur between relatives, and is fully consistent with standard invasion analysis (Lehmann et al., 2016). Following this framework, we derive $W(\boldsymbol{x}_\bullet, \bar{\boldsymbol{x}})$ by adapting these arguments to the present setting.

To that end, we treat our model as a class-structured population where an individual's class is its realized knowledge at the end of the learning process. The model presented in section 2.2 allows us to define the class-specific fitness function $w(k' \mid k, \boldsymbol{x}_\bullet, \bar{\boldsymbol{x}})$, which is the expected number of adults of class k' at the onset of stage (1) produced by a single adult of class k at the onset of stage (1) in the previous generation. Crucially, this class-specific fitness depends on the stationary distribution of knowledge in the resident population (captured by the argument $\bar{\boldsymbol{x}}$), as individuals acquire knowledge obliquely from resident exemplars.

Now observe that $w(k' \mid k, \boldsymbol{x}_\bullet, \bar{\boldsymbol{x}})$ is the mean transition density (or kernel) of an infinite-dimensional multitype branching process (Harris, 1963, Chapter 3) describing the dynamics of an $\boldsymbol{x}_\bullet$ lineage started from a single mutation introduced into an $\bar{\boldsymbol{x}}$ population. Standard results for such processes apply, provided this transition density satisfies Condition 10.1 p.67 of Harris (1963), which requires that an individual of any given class has a positive probability of producing descendants in any other class within a finite number of generations. In our model, this holds because the stochastic nature of learning ensures that offspring can eventually reach any knowledge.

The mutant fate is then fully determined by the leading eigenvalue $W(\boldsymbol{x}_\bullet, \bar{\boldsymbol{x}})$ of the linear operator determined by the transition density $w(k' \mid k, \boldsymbol{x}_\bullet, \bar{\boldsymbol{x}})$. The $\boldsymbol{x}_\bullet$ lineage goes extinct with probability one if $W(\boldsymbol{x}_\bullet, \bar{\boldsymbol{x}}) \leq 1$ and the probability of extinction is lower than one if $W(\boldsymbol{x}_\bullet, \bar{\boldsymbol{x}}) > 1$ (Harris, 1963, Th.12.1, p.70 along Corollary to Th. 14.1, p.73). Hence, $W(\boldsymbol{x}_\bullet, \bar{\boldsymbol{x}})$ satisfying

$$W(\boldsymbol{x}_\bullet, \bar{\boldsymbol{x}})\, \phi^*(k' \mid \boldsymbol{x}_\bullet) = \int w(k' \mid k, \boldsymbol{x}_\bullet, \bar{\boldsymbol{x}})\, \phi^*(k \mid \boldsymbol{x}_\bullet)\, \mathrm{d}k, \tag{B.1}$$

is the proper measure of lineage fitness (or invasion fitness) for our model. Here, $\phi^*(\cdot \mid \boldsymbol{x}_\bullet)$ is the right positive eigenfuction associated to the leading eigenvalue $W(\boldsymbol{x}_\bullet, \bar{\boldsymbol{x}})$, such that $\int \phi^*(k \mid \boldsymbol{x}_\bullet)\, \mathrm{d}k = 1$. The term $\phi^*(k \mid \boldsymbol{x}_\bullet)$ is the stationary probability density that an adult with traits $\boldsymbol{x}_\bullet$ carries knowledge value k at cultural equilibrium. Integrating both sides of eq. (B.1) over k', and using that $\int \phi^*(k' \mid \boldsymbol{x}_\bullet)\, \mathrm{d}k' = 1$ shows that

$$W(\boldsymbol{x}_\bullet, \bar{\boldsymbol{x}}) = \iint w(k' \mid k, \boldsymbol{x}_\bullet, \bar{\boldsymbol{x}})\, \phi^*(k \mid \boldsymbol{x}_\bullet)\, \mathrm{d}k\, \mathrm{d}k', \tag{B.2}$$

is the expected number of surviving offspring produced by a single adult of type $\boldsymbol{x}_\bullet$ (in a $\bar{\boldsymbol{x}}$ population).

Hereafter, we use k and k'' to denote the knowledge of offspring and parent, respectively, to remain consistent with the notation adopted in the previous appendix. From the model presented in section 2.2, we have

$$w(k \mid k'', \boldsymbol{x}_\bullet, \bar{\boldsymbol{x}}) = f(\boldsymbol{x}_\bullet, k'') \int \nu(k \mid \boldsymbol{x}_\bullet, k'', k')\, \phi^*(k' \mid \bar{\boldsymbol{x}})\, \mathrm{d}k'\, \frac{\tilde{s}(k)}{1 + \gamma\, n_{\mathrm{o}}^*(\bar{\boldsymbol{x}})}, \tag{B.3}$$

where $f(\boldsymbol{x}_\bullet, k'')$ (whose expression is given in eq. (1)) is the expected number of offspring produced by an adult with traits $\boldsymbol{x}_\bullet$ and knowledge k''. The term $\int \nu(k \mid \boldsymbol{x}_\bullet, k'', k')\, \phi^*(k' \mid \bar{\boldsymbol{x}})\, \mathrm{d}k'$ is the probability density that an offspring born from a parent with knowledge k'' acquires knowledge k. This is obtained by integrating the probability density $\nu(k \mid \boldsymbol{x}_\bullet, k'', k')$ that an

offspring with trait $\boldsymbol{x}_\bullet$, parental knowledge k'', and exemplar knowledge k' acquires knowledge k over the probability density of the exemplar's knowledge k'. Given that the variances of population traits are small, we approximate the population as being quasi-monomorphic for the mean traits $\bar{\boldsymbol{x}}$, so that the probability density of the exemplar's knowledge $\phi^*(k' \mid \bar{\boldsymbol{x}})$. Since $k = k_\bullet(1)$, where $k_\bullet(a)$ is a realization of the stochastic differential equation eq. (2), the probability density $\nu(k \mid \boldsymbol{x}_\bullet, k'', k')$ is entirely determined by eq. (2) (see, for example, the expression of $\nu(k \mid \boldsymbol{x}_\bullet, k'', k')$ given in eq. (A.26) when $\sigma_\mathrm{v} = \sigma_\mathrm{o} = 0$). Finally $\tilde{s}(k)/(1+\gamma\, n_\mathrm{o}^*(\bar{\boldsymbol{x}}))$ is the probability that an offspring with knowledge k survives to adulthood. In this term, $n_\mathrm{o}^*(\bar{\boldsymbol{x}})$ is the expected number of offspring produced at demographic and cultural equilibrium in a population with mean traits $\bar{\boldsymbol{x}}$.

Using the expression for $w(k \mid k'', \boldsymbol{x}_\bullet, \bar{\boldsymbol{x}})$ and $\tilde{s}(k)$ from eq. (B.3) with $k' = k$ and $k = k''$, and eq. (4) with $k_{\mathrm{o}\bullet} = k$, we rewrite eq. (B.2) as

$$W(\boldsymbol{x}_\bullet, \bar{\boldsymbol{x}}) = \int \Bigg[f(\boldsymbol{x}_\bullet, k'') \times \left(s_0 + \frac{\eta_\mathrm{s}}{1+\gamma\, n_\mathrm{o}^*(\bar{\boldsymbol{x}})} \iint k\, \nu(k \mid \boldsymbol{x}_\bullet, k'', k')\, \phi^*(k' \mid \bar{\boldsymbol{x}})\, \mathrm{d}k\, \mathrm{d}k'\, \phi^*(k'' \mid \boldsymbol{x}_\bullet) \right) \Bigg] \mathrm{d}k'', \quad \text{(B.4)}$$

which can be rewritten as

$$W(\boldsymbol{x}_\bullet, \bar{\boldsymbol{x}}) = \frac{s_0 \int f(\boldsymbol{x}_\bullet, k'')\, \phi^*(k'' \mid \boldsymbol{x}_\bullet)\, \mathrm{d}k''}{1+\gamma\, n_\mathrm{o}^*(\bar{\boldsymbol{x}})} + \frac{\eta_\mathrm{s} \iiint f(\boldsymbol{x}_\bullet, k'')\, k\, \nu(k \mid \boldsymbol{x}_\bullet, k'', k')\, \phi^*(k' \mid \bar{\boldsymbol{x}})\, \phi^*(k'' \mid \boldsymbol{x}_\bullet)\, \mathrm{d}k\, \mathrm{d}k'\, \mathrm{d}k''}{1+\gamma\, n_\mathrm{o}^*(\bar{\boldsymbol{x}})}. \quad \text{(B.5)}$$

Because $f(\boldsymbol{x}_\bullet, k'')$ depends linearly on k'' and $\int \phi^*(k'' \mid \boldsymbol{x}_\bullet)\, \mathrm{d}k'' = 1$ we have

$$\int f(\boldsymbol{x}_\bullet, k'')\, \phi^*(k'' \mid \boldsymbol{x}_\bullet)\, \mathrm{d}k'' = f\big(\boldsymbol{x}_\bullet, \int k''\, \phi^*(k'' \mid \boldsymbol{x}_\bullet)\, \mathrm{d}k''\big). \quad \text{(B.6)}$$

By definition, $\int k''\, \phi^*(k'' \mid \boldsymbol{x}_\bullet)\, \mathrm{d}k''$ is the expected knowledge of a random adult at cultural equilibrium in the $\boldsymbol{x}_\bullet$-lineage, which we denote as $\mathbb{E}_\mathrm{a}^*[k \mid \boldsymbol{x}_\bullet]$. Using this notation and eq. (B.6), and factorizing by $f\big(\boldsymbol{x}_\bullet, \mathbb{E}_\mathrm{a}^*[k \mid \boldsymbol{x}_\bullet]\big)/\big(1+\gamma\, n_\mathrm{o}^*(\bar{\boldsymbol{x}})\big)$, eq. (B.5) reduces to

$$W(\boldsymbol{x}_\bullet, \bar{\boldsymbol{x}}) = \frac{f\big(\boldsymbol{x}_\bullet, \mathbb{E}_\mathrm{a}^*[k \mid \boldsymbol{x}_\bullet]\big)}{1+\gamma\, n_\mathrm{o}^*(\bar{\boldsymbol{x}})} \Big[s_0 + \eta_\mathrm{s}\, \mathbb{E}_\mathrm{o}^*[k \mid \boldsymbol{x}_\bullet] \Big], \quad \text{(B.7)}$$

where

$$\mathbb{E}_{\mathrm{o}}^{*}[k \mid \boldsymbol{x}_{\bullet}] = \iiint k\, \nu(k \mid \boldsymbol{x}_{\bullet}, k'', k')\, \phi^{*}(k' \mid \bar{\boldsymbol{x}})\, \frac{f(\boldsymbol{x}_{\bullet}, k'')}{f\big(\boldsymbol{x}_{\bullet}, \mathbb{E}_{\mathrm{a}}^{*}[k \mid \boldsymbol{x}_{\bullet}]\big)}\, \phi^{*}(k'' \mid \boldsymbol{x}_{\bullet})\, \mathrm{d}k\, \mathrm{d}k'\, \mathrm{d}k''. \quad \text{(B.8)}$$

The term $\mathbb{E}_{\mathrm{o}}^{*}[k \mid \boldsymbol{x}_{\bullet}]$ is the expected knowledge of a random offspring once it has completed learning within the $\boldsymbol{x}_{\bullet}$-lineage at cultural equilibrium, taking into account the probability density of parental knowledge $f(\boldsymbol{x}_{\bullet}, k'')/f\big(\boldsymbol{x}_{\bullet}, \mathbb{E}_{\mathrm{a}}^{*}[k \mid \boldsymbol{x}_{\bullet}]\big)\, \phi^{*}(k'' \mid \boldsymbol{x}_{\bullet})$, the probability density of knowledge among potential oblique exemplars $\phi^{*}(k' \mid \bar{\boldsymbol{x}})$, and the probability density of stochastic learning outcomes $\nu(k \mid \boldsymbol{x}_{\bullet}, k'', k')$. Note that the probability density of parental knowledge is proportional to $f(\boldsymbol{x}_{\bullet}, k'')/f\big(\boldsymbol{x}_{\bullet}, \mathbb{E}_{\mathrm{a}}^{*}[k \mid \boldsymbol{x}_{\bullet}]\big)$ because lineage members with higher fecundity are overrepresented among parents and, consequently, among vertical exemplars.

By replacing $s_0 + \eta_{\mathrm{s}}\, \mathbb{E}_{\mathrm{o}}^{*}[k \mid \boldsymbol{x}_{\bullet}]$ by $\tilde{s}\big(\mathbb{E}_{\mathrm{o}}^{*}[k \mid \boldsymbol{x}_{\bullet}]\big)$ (obtained using eq. (4) with $k_{\mathrm{o}\bullet} = \mathbb{E}_{\mathrm{o}}^{*}[k \mid \boldsymbol{x}_{\bullet}]$) we obtain

$$W(\boldsymbol{x}_{\bullet}, \bar{\boldsymbol{x}}) = \frac{f\big(\boldsymbol{x}_{\bullet}, \mathbb{E}_{\mathrm{a}}^{*}[k \mid \boldsymbol{x}_{\bullet}]\big)\, \tilde{s}\big(\mathbb{E}_{\mathrm{o}}^{*}[k \mid \boldsymbol{x}_{\bullet}]\big)}{1 + \gamma\, n_{\mathrm{o}}^{*}(\bar{\boldsymbol{x}})}. \quad \text{(B.9)}$$

Now note that at equilibrium we have $n_{\mathrm{o}}^{*}(\bar{\boldsymbol{x}}) = f\big(\bar{\boldsymbol{x}}, \mathbb{E}_{\mathrm{a}}^{*}[k \mid \bar{\boldsymbol{x}}]\big)\, n_{\mathrm{a}}^{*}(\bar{\boldsymbol{x}})$ where $n_{\mathrm{a}}^{*}(\bar{\boldsymbol{x}})$ is the number of adults in the population. By substituting this expression into eq. (B.9) we obtain

$$W(\boldsymbol{x}_{\bullet}, \bar{\boldsymbol{x}}) = \frac{f\big(\boldsymbol{x}_{\bullet}, \mathbb{E}_{\mathrm{a}}^{*}[k \mid \boldsymbol{x}_{\bullet}]\big)\, \tilde{s}\big(\mathbb{E}_{\mathrm{o}}^{*}[k \mid \boldsymbol{x}_{\bullet}]\big)}{1 + \gamma f\big(\bar{\boldsymbol{x}}, \mathbb{E}_{\mathrm{a}}^{*}[k \mid \bar{\boldsymbol{x}}]\big)\, n_{\mathrm{a}}^{*}(\bar{\boldsymbol{x}})}. \quad \text{(B.10)}$$

To move forward, we derive an expression for $n_{\mathrm{a}}^{*}(\bar{\boldsymbol{x}})$. At demographic equilibrium, each adult, on average, produces one viable offspring to replace itself, so that

$$W(\bar{\boldsymbol{x}}, \bar{\boldsymbol{x}}) = 1. \quad \text{(B.11)}$$

By substituting the expression for $W(\bar{\boldsymbol{x}}, \bar{\boldsymbol{x}})$ from eq. (B.10) with $\boldsymbol{x}_{\bullet} = \bar{\boldsymbol{x}}$ into eq. (B.11) and solving for $n_{\mathrm{a}}^{*}(\bar{\boldsymbol{x}})$ we obtain

$$n_{\mathrm{a}}^{*}(\bar{\boldsymbol{x}}) = \frac{f\big(\bar{\boldsymbol{x}}, \mathbb{E}_{\mathrm{a}}^{*}[k \mid \bar{\boldsymbol{x}}]\big)\, \tilde{s}\big(\mathbb{E}_{\mathrm{o}}^{*}[k \mid \bar{\boldsymbol{x}}]\big) - 1}{\gamma\, f\big(\bar{\boldsymbol{x}}, \mathbb{E}_{\mathrm{a}}^{*}[k \mid \bar{\boldsymbol{x}}]\big)}, \quad \text{(B.12)}$$

where $\mathbb{E}_{\mathrm{a}}^{*}[k \mid \bar{\boldsymbol{x}}]$ and $\mathbb{E}_{\mathrm{o}}^{*}[k \mid \bar{\boldsymbol{x}}]$ are the expected knowledge at cultural equilibrium for a random adult and a random offspring after learning is complete in an $\bar{\boldsymbol{x}}$-lineage. Because trait variances are small, $\mathbb{E}_{\mathrm{a}}^{*}[k \mid \bar{\boldsymbol{x}}]$ and $\mathbb{E}_{\mathrm{o}}^{*}[k \mid \bar{\boldsymbol{x}}]$ are equal to the mean adult and offspring knowledge in the population at cultural equilibrium.

By substituting the expression for $n_{\mathrm{a}}^*(\bar{\boldsymbol{x}})$ from eq. (B.12) into eq. (B.10), we obtain

$$\boxed{W(\boldsymbol{x}_\bullet, \bar{\boldsymbol{x}}) = \frac{f\left(\boldsymbol{x}_\bullet, \mathbb{E}_{\mathrm{a}}^*[k \mid \boldsymbol{x}_\bullet]\right) \tilde{s}\left(\mathbb{E}_{\mathrm{o}}^*[k \mid \boldsymbol{x}_\bullet]\right)}{f\left(\bar{\boldsymbol{x}}, \mathbb{E}_{\mathrm{a}}^*[k \mid \bar{\boldsymbol{x}}]\right) \tilde{s}\left(\mathbb{E}_{\mathrm{o}}^*[k \mid \bar{\boldsymbol{x}}]\right)},} \tag{B.13}$$

which is eq. (5) of the main text.

B.2 Learning traits effect on lineage knowledge

Here, we derive the conditions that $\nabla\mathbb{E}_{\mathrm{a}}^*[k \mid \boldsymbol{x}_\bullet]|_{\boldsymbol{x}_\bullet=\bar{\boldsymbol{x}}}$ and $\nabla\mathbb{E}_{\mathrm{o}}^*[k \mid \boldsymbol{x}_\bullet]|_{\boldsymbol{x}_\bullet=\bar{\boldsymbol{x}}}$ satisfy.

We first derive eq. (14) from the main text, which give the conditions that $\nabla\mathbb{E}_{\mathrm{a}}^*[k \mid \boldsymbol{x}_\bullet]|_{\boldsymbol{x}_\bullet=\bar{\boldsymbol{x}}}$ satisfies. Differentiating both sides of eq. (A.56) with respect to the traits $\boldsymbol{x}_\bullet$ and evaluating at $\boldsymbol{x}_\bullet = \bar{\boldsymbol{x}}$, we obtain

$$\begin{aligned}
&\nabla\mathbb{E}_{\mathrm{a}}^*[k \mid \boldsymbol{x}_\bullet]|_{\boldsymbol{x}_\bullet=\bar{\boldsymbol{x}}} = \\
&h_{\mathrm{vl}}(\bar{\boldsymbol{x}})\ \nabla\mathbb{E}_{\mathrm{a}}^*[k \mid \boldsymbol{x}_\bullet]|_{\boldsymbol{x}_\bullet=\bar{\boldsymbol{x}}} + \nabla h_{\mathrm{vl}}(\boldsymbol{x}_\bullet)|_{\boldsymbol{x}_\bullet=\bar{\boldsymbol{x}}}\ \mathbb{E}_{\mathrm{a}}^*[k \mid \bar{\boldsymbol{x}}] + \nabla h_{\mathrm{ol}}(\boldsymbol{x}_\bullet)|_{\boldsymbol{x}_\bullet=\bar{\boldsymbol{x}}}\ \mathbb{E}_{\mathrm{a}}^*[k \mid \bar{\boldsymbol{x}}] + \nabla p_{\mathrm{il}}(\boldsymbol{x}_\bullet)|_{\boldsymbol{x}_\bullet=\bar{\boldsymbol{x}}} \\
&+\nabla h_{\mathrm{vl}}(\boldsymbol{x}_\bullet)|_{\boldsymbol{x}_\bullet=\bar{\boldsymbol{x}}}\ \frac{\mathrm{Var}_{\mathrm{a}}^*[k \mid \bar{\boldsymbol{x}}]\,\eta_{\mathrm{f}}}{f_0 + \eta_{\mathrm{f}}\,\mathbb{E}_{\mathrm{a}}^*[k \mid \boldsymbol{x}_\bullet]} + h_{\mathrm{vl}}(\bar{\boldsymbol{x}})\ \nabla\frac{\mathrm{Var}_{\mathrm{a}}^*[k \mid \boldsymbol{x}_\bullet]\,\eta_{\mathrm{f}}}{f_0 + \eta_{\mathrm{f}}\,\mathbb{E}_{\mathrm{a}}^*[k \mid \boldsymbol{x}_\bullet]}\bigg|_{\boldsymbol{x}_\bullet=\bar{\boldsymbol{x}}} + \nabla\frac{\mathrm{Var}_{\mathrm{o}}^*[k \mid \boldsymbol{x}_\bullet]\,\eta_{\mathrm{s}}}{\tilde{s}\left(\mathbb{E}_{\mathrm{o}}^*[k \mid \boldsymbol{x}_\bullet]\right)}\bigg|_{\boldsymbol{x}_\bullet=\bar{\boldsymbol{x}}}.
\end{aligned} \tag{B.14}$$

Equation (B.14) can be rewritten as

$$\boxed{\begin{aligned}
&\nabla\mathbb{E}_{\mathrm{a}}^*[k \mid \boldsymbol{x}_\bullet]|_{\boldsymbol{x}_\bullet=\bar{\boldsymbol{x}}} = \frac{1}{1 - h_{\mathrm{vl}}(\bar{\boldsymbol{x}})} \times \\
&\Bigg[\nabla p_{\mathrm{il}}(\boldsymbol{x}_\bullet)|_{\boldsymbol{x}_\bullet=\bar{\boldsymbol{x}}} + \nabla h_{\mathrm{ol}}(\boldsymbol{x}_\bullet)|_{\boldsymbol{x}_\bullet=\bar{\boldsymbol{x}}}\ \mathbb{E}_{\mathrm{a}}^*[k \mid \bar{\boldsymbol{x}}] + \nabla h_{\mathrm{vl}}(\boldsymbol{x}_\bullet)|_{\boldsymbol{x}_\bullet=\bar{\boldsymbol{x}}}\ \left(\mathbb{E}_{\mathrm{a}}^*[k \mid \bar{\boldsymbol{x}}] + \frac{\mathrm{Var}_{\mathrm{a}}^*[k \mid \bar{\boldsymbol{x}}]\,\eta_{\mathrm{f}}}{f_0 + \eta_{\mathrm{f}}\,\mathbb{E}_{\mathrm{a}}^*[k \mid \boldsymbol{x}_\bullet]}\right) \\
&+ h_{\mathrm{vl}}(\bar{\boldsymbol{x}})\ \nabla\frac{\mathrm{Var}_{\mathrm{a}}^*[k \mid \boldsymbol{x}_\bullet]\,\eta_{\mathrm{f}}}{f_0 + \eta_{\mathrm{f}}\,\mathbb{E}_{\mathrm{a}}^*[k \mid \boldsymbol{x}_\bullet]}\bigg|_{\boldsymbol{x}_\bullet=\bar{\boldsymbol{x}}} + \nabla\frac{\mathrm{Var}_{\mathrm{o}}^*[k \mid \boldsymbol{x}_\bullet]\,\eta_{\mathrm{s}}}{\tilde{s}\left(\mathbb{E}_{\mathrm{o}}^*[k \mid \boldsymbol{x}_\bullet]\right)}\bigg|_{\boldsymbol{x}_\bullet=\bar{\boldsymbol{x}}}\Bigg],
\end{aligned}} \tag{B.15}$$

which is eq. (14) of the main text.

We now derive the conditions that $\nabla\mathbb{E}_{\mathrm{o}}^*[k \mid \boldsymbol{x}_\bullet]|_{\boldsymbol{x}_\bullet=\bar{\boldsymbol{x}}}$ satisfies. Differentiating both sides of eq. (A.55) with respect to the traits $\boldsymbol{x}_\bullet$ and evaluating at $\boldsymbol{x}_\bullet = \bar{\boldsymbol{x}}$, we obtain

$$\nabla \mathbb{E}^*_{\mathrm{o}}[k \mid \boldsymbol{x}_\bullet]|_{\boldsymbol{x}_\bullet=\bar{\boldsymbol{x}}} =$$

$$h_{\mathrm{vl}}(\bar{\boldsymbol{x}}) \ \nabla \mathbb{E}^*_{\mathrm{a}}[k \mid \boldsymbol{x}_\bullet]|_{\boldsymbol{x}_\bullet=\bar{\boldsymbol{x}}} + \nabla h_{\mathrm{vl}}(\boldsymbol{x}_\bullet)|_{\boldsymbol{x}_\bullet=\bar{\boldsymbol{x}}} \ \mathbb{E}^*_{\mathrm{a}}[k \mid \bar{\boldsymbol{x}}] + \nabla h_{\mathrm{ol}}(\boldsymbol{x}_\bullet)|_{\boldsymbol{x}_\bullet=\bar{\boldsymbol{x}}} \ \mathbb{E}^*_{\mathrm{a}}[k \mid \bar{\boldsymbol{x}}] + \nabla p_{\mathrm{il}}(\boldsymbol{x}_\bullet)|_{\boldsymbol{x}_\bullet=\bar{\boldsymbol{x}}}$$
$$+ \nabla h_{\mathrm{vl}}(\boldsymbol{x}_\bullet)|_{\boldsymbol{x}_\bullet=\bar{\boldsymbol{x}}} \ \frac{\mathrm{Var}^*_{\mathrm{a}}[k \mid \bar{\boldsymbol{x}}] \, \eta_{\mathrm{f}}}{f_0 + \eta_{\mathrm{f}} \, \mathbb{E}^*_{\mathrm{a}}[k \mid \boldsymbol{x}_\bullet]} + h_{\mathrm{vl}}(\bar{\boldsymbol{x}}) \ \nabla \frac{\mathrm{Var}^*_{\mathrm{a}}[k \mid \boldsymbol{x}_\bullet] \, \eta_{\mathrm{f}}}{f_0 + \eta_{\mathrm{f}} \, \mathbb{E}^*_{\mathrm{a}}[k \mid \boldsymbol{x}_\bullet]}\Bigg|_{\boldsymbol{x}_\bullet=\bar{\boldsymbol{x}}} . \quad \text{(B.16)}$$

By substituting the expression of $\mathbb{E}^*_{\mathrm{a}}[k \mid \boldsymbol{x}_\bullet]$ from the first line of eq. (A.54) into eq. (B.16) and rearranging we obtain

$$\boxed{\begin{aligned} &\nabla \mathbb{E}^*_{\mathrm{o}}[k \mid \boldsymbol{x}_\bullet]|_{\boldsymbol{x}_\bullet=\bar{\boldsymbol{x}}} = \frac{1}{1 - h_{\mathrm{vl}}(\bar{\boldsymbol{x}})} \times \\ &\Bigg[\nabla p_{\mathrm{il}}(\boldsymbol{x}_\bullet)|_{\boldsymbol{x}_\bullet=\bar{\boldsymbol{x}}} + \nabla h_{\mathrm{ol}}(\boldsymbol{x}_\bullet)|_{\boldsymbol{x}_\bullet=\bar{\boldsymbol{x}}} \ \mathbb{E}^*_{\mathrm{a}}[k \mid \bar{\boldsymbol{x}}] + \nabla h_{\mathrm{vl}}(\boldsymbol{x}_\bullet)|_{\boldsymbol{x}_\bullet=\bar{\boldsymbol{x}}} \left(\mathbb{E}^*_{\mathrm{a}}[k \mid \bar{\boldsymbol{x}}] + \frac{\mathrm{Var}^*_{\mathrm{a}}[k \mid \bar{\boldsymbol{x}}] \, \eta_{\mathrm{f}}}{f_0 + \eta_{\mathrm{f}} \, \mathbb{E}^*_{\mathrm{a}}[k \mid \boldsymbol{x}_\bullet]} \right) \\ &+ h_{\mathrm{vl}}(\bar{\boldsymbol{x}}) \ \nabla \frac{\mathrm{Var}^*_{\mathrm{a}}[k \mid \boldsymbol{x}_\bullet] \, \eta_{\mathrm{f}}}{f_0 + \eta_{\mathrm{f}} \, \mathbb{E}^*_{\mathrm{a}}[k \mid \boldsymbol{x}_\bullet]}\Bigg|_{\boldsymbol{x}_\bullet=\bar{\boldsymbol{x}}} + h_{\mathrm{vl}}(\bar{\boldsymbol{x}}) \ \nabla \frac{\mathrm{Var}^*_{\mathrm{o}}[k \mid \boldsymbol{x}_\bullet] \, \eta_{\mathrm{s}}}{\tilde{s}\big(\mathbb{E}^*_{\mathrm{o}}[k \mid \boldsymbol{x}_\bullet]\big)}\Bigg|_{\boldsymbol{x}_\bullet=\bar{\boldsymbol{x}}} \Bigg], \end{aligned}}$$
$$\text{(B.17)}$$

which gives a condition satisfied by $\nabla \mathbb{E}^*_{\mathrm{o}}[k \mid \boldsymbol{x}_\bullet]|_{\boldsymbol{x}_\bullet=\bar{\boldsymbol{x}}}$.

We next link the conditions satisfied by $\nabla \mathbb{E}^*_{\mathrm{a}}[k \mid \boldsymbol{x}_\bullet]|_{\boldsymbol{x}_\bullet=\bar{\boldsymbol{x}}}$ and $\nabla \mathbb{E}^*_{\mathrm{o}}[k \mid \boldsymbol{x}_\bullet]|_{\boldsymbol{x}_\bullet=\bar{\boldsymbol{x}}}$. To this end, we rewrite (B.17) in terms of the expression for $\nabla \mathbb{E}^*_{\mathrm{a}}[k \mid \boldsymbol{x}_\bullet]|_{\boldsymbol{x}_\bullet=\bar{\boldsymbol{x}}}$ given in eq. (B.15)

$$\nabla \mathbb{E}^*_{\mathrm{o}}[k \mid \boldsymbol{x}_\bullet]|_{\boldsymbol{x}_\bullet=\bar{\boldsymbol{x}}} = \frac{1}{1 - h_{\mathrm{vl}}(\bar{\boldsymbol{x}})} \times$$
$$\Bigg[\nabla p_{\mathrm{il}}(\boldsymbol{x}_\bullet)|_{\boldsymbol{x}_\bullet=\bar{\boldsymbol{x}}} + \nabla h_{\mathrm{ol}}(\boldsymbol{x}_\bullet)|_{\boldsymbol{x}_\bullet=\bar{\boldsymbol{x}}} \ \mathbb{E}^*_{\mathrm{a}}[k \mid \bar{\boldsymbol{x}}] + \nabla h_{\mathrm{vl}}(\boldsymbol{x}_\bullet)|_{\boldsymbol{x}_\bullet=\bar{\boldsymbol{x}}} \left(\mathbb{E}^*_{\mathrm{a}}[k \mid \bar{\boldsymbol{x}}] + \frac{\mathrm{Var}^*_{\mathrm{a}}[k \mid \bar{\boldsymbol{x}}] \, \eta_{\mathrm{f}}}{f_0 + \eta_{\mathrm{f}} \, \mathbb{E}^*_{\mathrm{a}}[k \mid \boldsymbol{x}_\bullet]} \right)$$
$$+ h_{\mathrm{vl}}(\bar{\boldsymbol{x}}) \ \nabla \frac{\mathrm{Var}^*_{\mathrm{a}}[k \mid \boldsymbol{x}_\bullet] \, \eta_{\mathrm{f}}}{f_0 + \eta_{\mathrm{f}} \, \mathbb{E}^*_{\mathrm{a}}[k \mid \boldsymbol{x}_\bullet]}\Bigg|_{\boldsymbol{x}_\bullet=\bar{\boldsymbol{x}}} + \nabla \frac{\mathrm{Var}^*_{\mathrm{o}}[k \mid \boldsymbol{x}_\bullet] \, \eta_{\mathrm{s}}}{\tilde{s}\big(\mathbb{E}^*_{\mathrm{o}}[k \mid \boldsymbol{x}_\bullet]\big)}\Bigg|_{\boldsymbol{x}_\bullet=\bar{\boldsymbol{x}}} \Bigg]$$
$$+ \frac{1}{1 - h_{\mathrm{vl}}(\bar{\boldsymbol{x}})} (h_{\mathrm{vl}}(\bar{\boldsymbol{x}}) - 1) \ \nabla \frac{\mathrm{Var}^*_{\mathrm{o}}[k \mid \boldsymbol{x}_\bullet] \, \eta_{\mathrm{s}}}{\tilde{s}\big(\mathbb{E}^*_{\mathrm{o}}[k \mid \boldsymbol{x}_\bullet]\big)}\Bigg|_{\boldsymbol{x}_\bullet=\bar{\boldsymbol{x}}} , \quad \text{(B.18)}$$

which simplifies to

$$\boxed{\nabla \mathbb{E}^*_{\mathrm{o}}[k \mid \boldsymbol{x}_\bullet]|_{\boldsymbol{x}_\bullet=\bar{\boldsymbol{x}}} = \nabla \mathbb{E}^*_{\mathrm{a}}[k \mid \boldsymbol{x}_\bullet]|_{\boldsymbol{x}_\bullet=\bar{\boldsymbol{x}}} - \nabla \frac{\mathrm{Var}^*_{\mathrm{o}}[k \mid \boldsymbol{x}_\bullet] \, \eta_{\mathrm{s}}}{\tilde{s}\big(\mathbb{E}^*_{\mathrm{o}}[k \mid \boldsymbol{x}_\bullet]\big)}\Bigg|_{\boldsymbol{x}_\bullet=\bar{\boldsymbol{x}}} .} \quad \text{(B.19)}$$

B.3 Numerical estimation of the average learning traits favored by selection

Let $\bar{\boldsymbol{x}}_t$ denote the mean trait values in generation t. The expected change in these mean trait values from one generation to the next is given by

$$\bar{\boldsymbol{x}}_{t+1} = \bar{\boldsymbol{x}}_t + \boldsymbol{G} \cdot \boldsymbol{S}(\bar{\boldsymbol{x}}_t), \tag{B.20}$$

where $\boldsymbol{G}$ is the traits covariance matrix, assumed to be constant here (Iwasa et al., 1991; Mullon and Lehmann, 2019).

To numerically determine the mean trait values favored by selection $\bar{\boldsymbol{x}}^*$, we first need to obtain the full expression for the selection gradient $\boldsymbol{S}(\bar{\boldsymbol{x}}_t)$. This requires deriving an exact expression for $\nabla \mathbb{E}^*_{\text{a}}[k \mid \boldsymbol{x}_\bullet]|_{\boldsymbol{x}_\bullet = \bar{\boldsymbol{x}}_t}$ and $\nabla \mathbb{E}^*_{\text{o}}[k \mid \boldsymbol{x}_\bullet]|_{\boldsymbol{x}_\bullet = \bar{\boldsymbol{x}}_t}$.

To simplify notation, we rewrite eq. (A.54) in functional form

$$\begin{cases} \mathbb{E}^*_{\text{a}}[k \mid \boldsymbol{x}_\bullet] = \mathcal{F}_1\big(\mathbb{E}^*_{\text{a}}[k \mid \boldsymbol{x}_\bullet], \mathbb{E}^*_{\text{o}}[k \mid \boldsymbol{x}_\bullet], \text{Var}^*_{\text{a}}[k \mid \boldsymbol{x}_\bullet], \text{Var}^*_{\text{o}}[k \mid \boldsymbol{x}_\bullet]\big) \\ \mathbb{E}^*_{\text{o}}[k \mid \boldsymbol{x}_\bullet] = \mathcal{F}_2\big(\mathbb{E}^*_{\text{a}}[k \mid \boldsymbol{x}_\bullet], \mathbb{E}^*_{\text{o}}[k \mid \boldsymbol{x}_\bullet], \text{Var}^*_{\text{a}}[k \mid \boldsymbol{x}_\bullet], \text{Var}^*_{\text{o}}[k \mid \boldsymbol{x}_\bullet]\big) \\ \text{Var}^*_{\text{a}}[k \mid \boldsymbol{x}_\bullet] = \mathcal{F}_3\big(\mathbb{E}^*_{\text{a}}[k \mid \boldsymbol{x}_\bullet], \mathbb{E}^*_{\text{o}}[k \mid \boldsymbol{x}_\bullet], \text{Var}^*_{\text{a}}[k \mid \boldsymbol{x}_\bullet], \text{Var}^*_{\text{o}}[k \mid \boldsymbol{x}_\bullet]\big) \\ \text{Var}^*_{\text{o}}[k \mid \boldsymbol{x}_\bullet] = \mathcal{F}_4\big(\mathbb{E}^*_{\text{a}}[k \mid \boldsymbol{x}_\bullet], \mathbb{E}^*_{\text{o}}[k \mid \boldsymbol{x}_\bullet], \text{Var}^*_{\text{a}}[k \mid \boldsymbol{x}_\bullet], \text{Var}^*_{\text{o}}[k \mid \boldsymbol{x}_\bullet]\big), \end{cases} \tag{B.21}$$

where the functions $\mathcal{F}_1$, $\mathcal{F}_2$, $\mathcal{F}_3$ and $\mathcal{F}_4$ are defined by the right-hand sides of eq. (A.54).

Thus, by differentiating both sides of each line in the system (B.21) with respect to $\boldsymbol{x}_\bullet$ and evaluating at $\boldsymbol{x}_\bullet = \bar{\boldsymbol{x}}_t$, we obtain the system

$$\begin{cases} \nabla \mathbb{E}^*_{\text{a}}[k \mid \boldsymbol{x}_\bullet]|_{\boldsymbol{x}_\bullet = \bar{\boldsymbol{x}}_t} = \nabla \mathcal{F}_1\big(\mathbb{E}^*_{\text{a}}[k \mid \boldsymbol{x}_\bullet], \mathbb{E}^*_{\text{o}}[k \mid \boldsymbol{x}_\bullet], \text{Var}^*_{\text{a}}[k \mid \boldsymbol{x}_\bullet], \text{Var}^*_{\text{o}}[k \mid \boldsymbol{x}_\bullet]\big)\big|_{\boldsymbol{x}_\bullet = \bar{\boldsymbol{x}}_t} \\ \nabla \mathbb{E}^*_{\text{o}}[k \mid \boldsymbol{x}_\bullet]|_{\boldsymbol{x}_\bullet = \bar{\boldsymbol{x}}_t} = \nabla \mathcal{F}_2\big(\mathbb{E}^*_{\text{a}}[k \mid \boldsymbol{x}_\bullet], \mathbb{E}^*_{\text{o}}[k \mid \boldsymbol{x}_\bullet], \text{Var}^*_{\text{a}}[k \mid \boldsymbol{x}_\bullet], \text{Var}^*_{\text{o}}[k \mid \boldsymbol{x}_\bullet]\big)\big|_{\boldsymbol{x}_\bullet = \bar{\boldsymbol{x}}_t} \\ \nabla \text{Var}^*_{\text{a}}[k \mid \boldsymbol{x}_\bullet]|_{\boldsymbol{x}_\bullet = \bar{\boldsymbol{x}}_t} = \nabla \mathcal{F}_3\big(\mathbb{E}^*_{\text{a}}[k \mid \boldsymbol{x}_\bullet], \mathbb{E}^*_{\text{o}}[k \mid \boldsymbol{x}_\bullet], \text{Var}^*_{\text{a}}[k \mid \boldsymbol{x}_\bullet], \text{Var}^*_{\text{o}}[k \mid \boldsymbol{x}_\bullet]\big)\big|_{\boldsymbol{x}_\bullet = \bar{\boldsymbol{x}}_t} \\ \nabla \text{Var}^*_{\text{o}}[k \mid \boldsymbol{x}_\bullet]|_{\boldsymbol{x}_\bullet = \bar{\boldsymbol{x}}_t} = \nabla \mathcal{F}_4\big(\mathbb{E}^*_{\text{a}}[k \mid \boldsymbol{x}_\bullet], \mathbb{E}^*_{\text{o}}[k \mid \boldsymbol{x}_\bullet], \text{Var}^*_{\text{a}}[k \mid \boldsymbol{x}_\bullet], \text{Var}^*_{\text{o}}[k \mid \boldsymbol{x}_\bullet]\big)\big|_{\boldsymbol{x}_\bullet = \bar{\boldsymbol{x}}_t} \end{cases} . \tag{B.22}$$

By solving eq. (B.22) for $\nabla \mathbb{E}^*_{\text{a}}[k \mid \boldsymbol{x}_\bullet]|_{\boldsymbol{x}_\bullet = \bar{\boldsymbol{x}}_t}$, $\nabla \mathbb{E}^*_{\text{o}}[k \mid \boldsymbol{x}_\bullet]|_{\boldsymbol{x}_\bullet = \bar{\boldsymbol{x}}_t}$, $\nabla \text{Var}^*_{\text{a}}[k \mid \boldsymbol{x}_\bullet]|_{\boldsymbol{x}_\bullet = \bar{\boldsymbol{x}}_t}$ and $\nabla \text{Var}^*_{\text{o}}[k \mid \boldsymbol{x}_\bullet]|_{\boldsymbol{x}_\bullet = \bar{\boldsymbol{x}}_t}$ using the *Solve* function in Wolfram Mathematica 13.0.0, we obtain an exact expression for $\nabla \mathbb{E}^*_{\text{a}}[k \mid \boldsymbol{x}_\bullet]|_{\boldsymbol{x}_\bullet = \bar{\boldsymbol{x}}_t}$ and $\nabla \mathbb{E}^*_{\text{o}}[k \mid \boldsymbol{x}_\bullet]|_{\boldsymbol{x}_\bullet = \bar{\boldsymbol{x}}_t}$. These results enable us to derive

an exact expression for the selection gradient $\boldsymbol{S}(\bar{\boldsymbol{x}}_t)$ (see the Mathematica Notebook in the supplementary material).

With the expression of the selection gradient in hand, we can now numerically track the evolution of mean traits across generations. We iterated the dynamics described by eq. (B.20) with $\boldsymbol{G} = 0.1\boldsymbol{I}$ where $\boldsymbol{I}$ is the identity matrix, starting from the initial conditions $\bar{\boldsymbol{x}}_1 = (0,0,0)$ and $\mathbb{E}^*_{\mathrm{a}}[k \mid \bar{\boldsymbol{x}}_1] = \mathrm{Var}^*_{\mathrm{a}}[k \mid \bar{\boldsymbol{x}}_1] = 0$. For each subsequent generation $t > 1$, $\mathbb{E}^*_{\mathrm{a}}[k \mid \bar{\boldsymbol{x}}_t]$ and $\mathrm{Var}^*_{\mathrm{a}}[k \mid \bar{\boldsymbol{x}}_t]$ are recalculated using the procedure described in section A.4.4, setting $\bar{\boldsymbol{x}} = \bar{\boldsymbol{x}}_t$ and initializing the procedure with the equilibrium values of $\mathbb{E}^*_{\mathrm{a}}[k \mid \bar{\boldsymbol{x}}_{t-1}]$ and $\mathrm{Var}^*_{\mathrm{a}}[k \mid \bar{\boldsymbol{x}}_{t-1}]$ from the previous generation. We iterate this dynamics until $\sqrt{(\bar{v}_{t+1} - \bar{v}_t)^2 + (\bar{o}_{t+1} - \bar{o}_t)^2 + \left(\bar{\lambda}_{t+1} - \bar{\lambda}_t\right)^2} < 10^{-7}$.

Once the mean traits converge to an interior singular point $\bar{\boldsymbol{x}}^*$ at which directional selection vanishes (i.e., $\boldsymbol{S}(\bar{\boldsymbol{x}}^*) = 0$), selection may become disruptive, potentially leading to evolutionary branching whereby trait distribution becomes bimodal (Geritz et al., 2016). To assess we compute the Hessian matrix

$$\boldsymbol{H}(\boldsymbol{x}_\bullet) = \begin{pmatrix} \left.\dfrac{\partial^2 W(\boldsymbol{x}_\bullet, \bar{\boldsymbol{x}}^*)}{\partial v_\bullet^2}\right|_{\boldsymbol{x}_\bullet = \bar{\boldsymbol{x}}} & \left.\dfrac{\partial^2 W(\boldsymbol{x}_\bullet, \bar{\boldsymbol{x}}^*)}{\partial v_\bullet \partial o_\bullet}\right|_{\boldsymbol{x}_\bullet = \bar{\boldsymbol{x}}} & \left.\dfrac{\partial^2 W(\boldsymbol{x}_\bullet, \bar{\boldsymbol{x}}^*)}{\partial v_\bullet \partial \lambda_\bullet}\right|_{\boldsymbol{x}_\bullet = \bar{\boldsymbol{x}}} \\ \left.\dfrac{\partial^2 W(\boldsymbol{x}_\bullet, \bar{\boldsymbol{x}}^*)}{\partial v_\bullet \partial o_\bullet}\right|_{\boldsymbol{x}_\bullet = \bar{\boldsymbol{x}}} & \left.\dfrac{\partial^2 W(\boldsymbol{x}_\bullet, \bar{\boldsymbol{x}}^*)}{\partial o_\bullet^2}\right|_{\boldsymbol{x}_\bullet = \bar{\boldsymbol{x}}} & \left.\dfrac{\partial^2 W(\boldsymbol{x}_\bullet, \bar{\boldsymbol{x}}^*)}{\partial o_\bullet \partial \lambda_\bullet}\right|_{\boldsymbol{x}_\bullet = \bar{\boldsymbol{x}}} \\ \left.\dfrac{\partial^2 W(\boldsymbol{x}_\bullet, \bar{\boldsymbol{x}}^*)}{\partial v_\bullet \partial \lambda_\bullet}\right|_{\boldsymbol{x}_\bullet = \bar{\boldsymbol{x}}} & \left.\dfrac{\partial^2 W(\boldsymbol{x}_\bullet, \bar{\boldsymbol{x}}^*)}{\partial o_\bullet \partial \lambda_\bullet}\right|_{\boldsymbol{x}_\bullet = \bar{\boldsymbol{x}}} & \left.\dfrac{\partial^2 W(\boldsymbol{x}_\bullet, \bar{\boldsymbol{x}}^*)}{\partial \lambda_\bullet^2}\right|_{\boldsymbol{x}_\bullet = \bar{\boldsymbol{x}}} \end{pmatrix}. \tag{B.23}$$

If the leading eigenvalue of $\boldsymbol{H}(\boldsymbol{x}_\bullet)$ is negative, selection is stabilizing and evolutionary branching cannot occur. In all cases considered, we find that the leading eigenvalue of $\boldsymbol{H}(\boldsymbol{x}_\bullet)$ is negative, confirming the absence of evolutionary branching.

Numerically evaluating $\boldsymbol{H}(\boldsymbol{x}_\bullet)$ requires second derivatives of $\mathbb{E}^*_{\mathrm{a}}[k \mid \boldsymbol{x}_\bullet]$ and $\mathbb{E}^*_{\mathrm{o}}[k \mid \boldsymbol{x}_\bullet]$ with respect to traits at $\boldsymbol{x}_\bullet = \bar{\boldsymbol{x}}^*$. These are obtained by differentiating system (B.21) twice with respect to traits $\boldsymbol{x}_\bullet$ at $\boldsymbol{x}_\bullet = \bar{\boldsymbol{x}}^*$ and solving the resulting system using the *Solve* function in Wolfram Mathematica 13.0.0.

B.4 Link to the findings of Maisonneuve et al. (2025)

Here, we present the connection with the findings of Maisonneuve et al. (2025). Maisonneuve et al. (2025) showed that oblique learning can evolve even if vertical learning is more efficient, because individuals can access knowledge from other adults that differs from their parents. Oblique learning evolves when the parameter ρ, defined in Maisonneuve et al. (2025) as the

probability that two individuals from the same generation produce simultaneously different knowledge during individual learning, is low, thereby limiting overlap in adult knowledge. In our model, we explicitly control the degree of knowledge overlap among adults using the parameter ρ as well. Although ρ is not defined identically in both models, in both cases, low values of ρ correspond to low overlap in the knowledge held by adults. Consistent with previous findings, we show that when adult knowledge does not fully overlap (i.e., low value of ρ), oblique learning can evolve, as it enables individuals to acquire information unavailable from their parents.

Appendix C: Selection pressure on stochasticity in learning

In this section, we introduce and derive a condition for the emergence of an additional trait $\zeta \in [0, +\infty)$ that affects stochasticity in individual learning $\sigma_i(\zeta)$. We assume that $\forall \zeta \in [0, +\infty)$, $\sigma_i'(\zeta) > 0$, meaning that higher values of ζ correspond to greater stochasticity in learning. We also assume that $\sigma_i(0) > 0$, with $\sigma_i(0)$ remaining small, reflecting that learning is inherently stochastic even in the absence of a specific trait promoting it.

Each individual is now characterized by a vector of traits $\boldsymbol{x}_\bullet = (v_\bullet, o_\bullet, \lambda_\bullet, \zeta_\bullet)$. We study how selection may favor the emergence of the trait ζ that increases stochasticity in learning, starting from a population with mean trait vector $\bar{\boldsymbol{x}}_0 = (\bar{v}, \bar{o}, \bar{\lambda}, 0)$. A trait located at its lower boundary will emerge if selection favors an increase in its population mean value, that is, if the corresponding component of the selection gradient is positive. Using eq. (13) we obtain that the trait ζ emerge if

$$\boxed{\frac{\eta_{\mathrm{f}}}{f_0 + \eta_{\mathrm{f}}\,\mathbb{E}_{\mathrm{a}}^*[k \mid \bar{\boldsymbol{x}}_0]} \left.\frac{\partial \mathbb{E}_{\mathrm{a}}^*[k \mid \boldsymbol{x}_\bullet]}{\partial \zeta_\bullet}\right|_{\boldsymbol{x}_\bullet = \bar{\boldsymbol{x}}_0} + \frac{\eta_{\mathrm{s}}}{\tilde{s}\big(\mathbb{E}_{\mathrm{o}}^*[k \mid \bar{\boldsymbol{x}}_0]\big)} \left.\frac{\partial \mathbb{E}_{\mathrm{o}}^*[k \mid \boldsymbol{x}_\bullet]}{\partial \zeta_\bullet}\right|_{\boldsymbol{x}_\bullet = \bar{\boldsymbol{x}}_0} > 0.} \tag{C.1}$$

To assess the emergence of ζ, we need to determine in the $\boldsymbol{x}_\bullet$-lineage the effect of $\zeta_\bullet$ on the expected knowledge of adults and offspring. Differentiating both sides of eq. (A.57) and the first line of eq. (A.54) with respect to $\zeta_\bullet$ (noting that $\mathbb{E}_{\mathrm{a}}^*[k \mid \boldsymbol{x}_\bullet]$ and $\mathbb{E}_{\mathrm{o}}^*[k \mid \boldsymbol{x}_\bullet]$ appear in both the numerator and denominator of the cultural selection term) and by substituting $\boldsymbol{x}_\bullet = \bar{\boldsymbol{x}}_0$ we find that

$$
\begin{aligned}
\left.\frac{\partial \mathbb{E}_{\mathrm{a}}^{*}[k \mid \boldsymbol{x}_{\bullet}]}{\partial \zeta_{\bullet}}\right|_{\boldsymbol{x}_{\bullet}=\bar{\boldsymbol{x}}_{0}} &= \frac{1}{1-h_{\mathrm{vl}}(\bar{\boldsymbol{x}}_{0})}\times \\
&\left[\frac{h_{\mathrm{vl}}(\bar{\boldsymbol{x}}_{0})\ \left.\frac{\partial \mathrm{Var}_{\mathrm{a}}^{*}[k|\boldsymbol{x}_{\bullet}]}{\partial \zeta_{\bullet}}\right|_{\boldsymbol{x}_{\bullet}=\bar{\boldsymbol{x}}_{0}}\ \eta_{\mathrm{f}}}{f_{0}+\eta_{\mathrm{f}}\,\mathbb{E}_{\mathrm{a}}^{*}[k \mid \bar{\boldsymbol{x}}_{0}]} - \frac{h_{\mathrm{vl}}(\bar{\boldsymbol{x}}_{0})\,\mathrm{Var}_{\mathrm{a}}^{*}[k \mid \bar{\boldsymbol{x}}_{0}]\,\eta_{\mathrm{f}}^{2}\ \left.\frac{\partial \mathbb{E}_{\mathrm{a}}^{*}[k|\boldsymbol{x}_{\bullet}]}{\partial \zeta_{\bullet}}\right|_{\boldsymbol{x}_{\bullet}=\bar{\boldsymbol{x}}_{0}}}{\left(f_{0}+\eta_{\mathrm{f}}\,\mathbb{E}_{\mathrm{a}}^{*}[k \mid \bar{\boldsymbol{x}}_{0}]\right)^{2}}\right. \\
&\left. + \frac{\left.\frac{\partial \mathrm{Var}_{\mathrm{o}}^{*}[k|\boldsymbol{x}_{\bullet}]}{\partial \zeta_{\bullet}}\right|_{\boldsymbol{x}_{\bullet}=\bar{\boldsymbol{x}}_{0}}\ \eta_{\mathrm{s}}}{\tilde{s}\left(\mathbb{E}_{\mathrm{o}}^{*}[k \mid \bar{\boldsymbol{x}}_{0}]\right)} - \frac{\mathrm{Var}_{\mathrm{o}}^{*}[k \mid \bar{\boldsymbol{x}}_{0}]\,\eta_{\mathrm{s}}^{2}\ \left.\frac{\partial \mathbb{E}_{\mathrm{o}}^{*}[k|\boldsymbol{x}_{\bullet}]}{\partial \zeta_{\bullet}}\right|_{\boldsymbol{x}_{\bullet}=\bar{\boldsymbol{x}}_{0}}}{\tilde{s}\left(\mathbb{E}_{\mathrm{o}}^{*}[k \mid \bar{\boldsymbol{x}}_{0}]\right)^{2}}\right],
\end{aligned}
\tag{C.2}
$$

and

$$
\begin{aligned}
\left.\frac{\partial \mathbb{E}_{\mathrm{a}}^{*}[k \mid \boldsymbol{x}_{\bullet}]}{\partial \zeta_{\bullet}}\right|_{\boldsymbol{x}_{\bullet}=\bar{\boldsymbol{x}}_{0}} &= \left.\frac{\partial \mathbb{E}_{\mathrm{o}}^{*}[k \mid \boldsymbol{x}_{\bullet}]}{\partial \zeta_{\bullet}}\right|_{\boldsymbol{x}_{\bullet}=\bar{\boldsymbol{x}}_{0}} \\
&+ \frac{\left.\frac{\partial \mathrm{Var}_{\mathrm{o}}^{*}[k|\boldsymbol{x}_{\bullet}]}{\partial \zeta_{\bullet}}\right|_{\boldsymbol{x}_{\bullet}=\bar{\boldsymbol{x}}_{0}}\ \eta_{\mathrm{s}}}{\tilde{s}\left(\mathbb{E}_{\mathrm{o}}^{*}[k \mid \bar{\boldsymbol{x}}_{0}]\right)} - \frac{\mathrm{Var}_{\mathrm{o}}^{*}[k \mid \bar{\boldsymbol{x}}_{0}]\,\eta_{\mathrm{s}}^{2}\ \left.\frac{\partial \mathbb{E}_{\mathrm{o}}^{*}[k|\boldsymbol{x}_{\bullet}]}{\partial \zeta_{\bullet}}\right|_{\boldsymbol{x}_{\bullet}=\bar{\boldsymbol{x}}_{0}}}{\tilde{s}\left(\mathbb{E}_{\mathrm{o}}^{*}[k \mid \bar{\boldsymbol{x}}_{0}]\right)^{2}}.
\end{aligned}
\tag{C.3}
$$

Solving eqs. (C.2) and (C.3) for $\left.\frac{\partial \mathbb{E}_{\mathrm{a}}^{*}[k|\boldsymbol{x}_{\bullet}]}{\partial \zeta_{\bullet}}\right|_{\boldsymbol{x}_{\bullet}=\bar{\boldsymbol{x}}_{0}}$ and $\left.\frac{\partial \mathbb{E}_{\mathrm{o}}^{*}[k|\boldsymbol{x}_{\bullet}]}{\partial \zeta_{\bullet}}\right|_{\boldsymbol{x}_{\bullet}=\bar{\boldsymbol{x}}_{0}}$ we obtain

$$
\begin{aligned}
&\left.\frac{\partial \mathbb{E}_{\mathrm{a}}^{*}[k \mid \boldsymbol{x}_{\bullet}]}{\partial \zeta_{\bullet}}\right|_{\boldsymbol{x}_{\bullet}=\bar{\boldsymbol{x}}_{0}} = \\
&\frac{1}{1-h_{\mathrm{vl}}(\bar{\boldsymbol{x}}_{0})\left(1-\left(\frac{\eta_{\mathrm{f}}}{f_{0}+\eta_{\mathrm{f}}\,\mathbb{E}_{\mathrm{a}}^{*}[k|\bar{\boldsymbol{x}}_{0}]}\right)^{2}\mathrm{Var}_{\mathrm{a}}^{*}[k \mid \bar{\boldsymbol{x}}_{0}]\right)\left(1-\left(\frac{\eta_{\mathrm{s}}}{\tilde{s}\left(\mathbb{E}_{\mathrm{o}}^{*}[k|\bar{\boldsymbol{x}}_{0}]\right)}\right)^{2}\mathrm{Var}_{\mathrm{o}}^{*}[k \mid \bar{\boldsymbol{x}}_{0}]\right)} \\
&\times\left[\frac{\left(1-\left(\frac{\eta_{\mathrm{s}}}{\tilde{s}\left(\mathbb{E}_{\mathrm{o}}^{*}[k|\bar{\boldsymbol{x}}_{0}]\right)}\right)^{2}\mathrm{Var}_{\mathrm{o}}^{*}[k \mid \bar{\boldsymbol{x}}_{0}]\right)h_{\mathrm{vl}}(\bar{\boldsymbol{x}}_{0})\ \left.\frac{\partial \mathrm{Var}_{\mathrm{a}}^{*}[k|\boldsymbol{x}_{\bullet}]}{\partial \zeta_{\bullet}}\right|_{\boldsymbol{x}_{\bullet}=\bar{\boldsymbol{x}}_{0}}\ \eta_{\mathrm{f}}}{f_{0}+\eta_{\mathrm{f}}\,\mathbb{E}_{\mathrm{a}}^{*}[k \mid \bar{\boldsymbol{x}}_{0}]} + \frac{\left.\frac{\partial \mathrm{Var}_{\mathrm{o}}^{*}[k|\boldsymbol{x}_{\bullet}]}{\partial \zeta_{\bullet}}\right|_{\boldsymbol{x}_{\bullet}=\bar{\boldsymbol{x}}_{0}}\ \eta_{\mathrm{s}}}{\tilde{s}\left(\mathbb{E}_{\mathrm{o}}^{*}[k \mid \bar{\boldsymbol{x}}_{0}]\right)}\right],
\end{aligned}
\tag{C.4}
$$

and

$$
\begin{aligned}
&\left.\frac{\partial \mathbb{E}_{\mathrm{o}}^{*}[k \mid \boldsymbol{x}_{\bullet}]}{\partial \zeta_{\bullet}}\right|_{\boldsymbol{x}_{\bullet}=\bar{\boldsymbol{x}}_{0}} = \\
&\frac{1}{1-h_{\mathrm{vl}}(\bar{\boldsymbol{x}}_{0})\left(1-\left(\frac{\eta_{\mathrm{f}}}{f_{0}+\eta_{\mathrm{f}}\,\mathbb{E}_{\mathrm{a}}^{*}[k|\bar{\boldsymbol{x}}_{0}]}\right)^{2}\mathrm{Var}_{\mathrm{a}}^{*}[k \mid \bar{\boldsymbol{x}}_{0}]\right)\left(1-\left(\frac{\eta_{\mathrm{s}}}{\tilde{s}\left(\mathbb{E}_{\mathrm{o}}^{*}[k|\bar{\boldsymbol{x}}_{0}]\right)}\right)^{2}\mathrm{Var}_{\mathrm{o}}^{*}[k \mid \bar{\boldsymbol{x}}_{0}]\right)} \\
&\times h_{\mathrm{vl}}(\bar{\boldsymbol{x}}_{0})\left[\frac{\left.\frac{\partial \mathrm{Var}_{\mathrm{a}}^{*}[k|\boldsymbol{x}_{\bullet}]}{\partial \zeta_{\bullet}}\right|_{\boldsymbol{x}_{\bullet}=\bar{\boldsymbol{x}}_{0}}\ \eta_{\mathrm{f}}}{f_{0}+\eta_{\mathrm{f}}\,\mathbb{E}_{\mathrm{a}}^{*}[k \mid \bar{\boldsymbol{x}}_{0}]} + \frac{\left(1-\left(\frac{\eta_{\mathrm{f}}}{f_{0}+\eta_{\mathrm{f}}\,\mathbb{E}_{\mathrm{a}}^{*}[k|\bar{\boldsymbol{x}}_{0}]}\right)^{2}\mathrm{Var}_{\mathrm{a}}^{*}[k \mid \bar{\boldsymbol{x}}_{0}]\right)\left.\frac{\partial \mathrm{Var}_{\mathrm{o}}^{*}[k|\boldsymbol{x}_{\bullet}]}{\partial \zeta_{\bullet}}\right|_{\boldsymbol{x}_{\bullet}=\bar{\boldsymbol{x}}_{0}}\ \eta_{\mathrm{s}}}{\tilde{s}\left(\mathbb{E}_{\mathrm{o}}^{*}[k \mid \bar{\boldsymbol{x}}_{0}]\right)}\right].
\end{aligned}
\tag{C.5}
$$

Since $\sigma_{\mathrm{i}}(0)$ is small at $\bar{\boldsymbol{x}}_0$, the variance in knowledge of both adults and offspring is small in

each lineage. In this regime, eqs. (C.4) and (C.5) simplify to

$$\boxed{\left.\frac{\partial \mathbb{E}_{\mathrm{a}}^{*}[k \mid \boldsymbol{x}_\bullet]}{\partial \zeta_\bullet}\right|_{\boldsymbol{x}_\bullet=\bar{\boldsymbol{x}}_0} \approx \frac{1}{1-h_{\mathrm{vl}}(\bar{\boldsymbol{x}}_0)}\left[\frac{h_{\mathrm{vl}}(\bar{\boldsymbol{x}}_0)\left.\frac{\partial \mathrm{Var}_{\mathrm{a}}^{*}[k|\boldsymbol{x}_\bullet]}{\partial \zeta_\bullet}\right|_{\boldsymbol{x}_\bullet=\bar{\boldsymbol{x}}_0}\eta_{\mathrm{f}}}{f_0+\eta_{\mathrm{f}}\,\mathbb{E}_{\mathrm{a}}^{*}[k \mid \bar{\boldsymbol{x}}_0]}+\frac{\left.\frac{\partial \mathrm{Var}_{\mathrm{o}}^{*}[k|\boldsymbol{x}_\bullet]}{\partial \zeta_\bullet}\right|_{\boldsymbol{x}_\bullet=\bar{\boldsymbol{x}}_0}\eta_{\mathrm{s}}}{\tilde{s}\big(\mathbb{E}_{\mathrm{o}}^{*}[k \mid \bar{\boldsymbol{x}}_0]\big)}\right],} \tag{C.6}$$

and

$$\boxed{\left.\frac{\partial \mathbb{E}_{\mathrm{o}}^{*}[k \mid \boldsymbol{x}_\bullet]}{\partial \zeta_\bullet}\right|_{\boldsymbol{x}_\bullet=\bar{\boldsymbol{x}}_0} \approx \frac{1}{1-h_{\mathrm{vl}}(\bar{\boldsymbol{x}}_0)}\times\left[\frac{h_{\mathrm{vl}}(\bar{\boldsymbol{x}}_0)\left.\frac{\partial \mathrm{Var}_{\mathrm{a}}^{*}[k|\boldsymbol{x}_\bullet]}{\partial \zeta_\bullet}\right|_{\boldsymbol{x}_\bullet=\bar{\boldsymbol{x}}_0}\eta_{\mathrm{f}}}{f_0+\eta_{\mathrm{f}}\,\mathbb{E}_{\mathrm{a}}^{*}[k \mid \bar{\boldsymbol{x}}_0]}+\frac{h_{\mathrm{vl}}(\bar{\boldsymbol{x}}_0)\left.\frac{\partial \mathrm{Var}_{\mathrm{o}}^{*}[k|\boldsymbol{x}_\bullet]}{\partial \zeta_\bullet}\right|_{\boldsymbol{x}_\bullet=\bar{\boldsymbol{x}}_0}\eta_{\mathrm{s}}}{\tilde{s}\big(\mathbb{E}_{\mathrm{o}}^{*}[k \mid \bar{\boldsymbol{x}}_0]\big)}\right].} \tag{C.7}$$

To proceed further, we determine the effect of $\zeta_\bullet$ on the variance of knowledge held by a randomly chosen adult and offspring within the $\boldsymbol{x}_\bullet$-lineage. By differentiating the third and fourth lines of eq. (A.54) with respect to $\zeta_\bullet$ and evaluating at $\boldsymbol{x}_\bullet=\bar{\boldsymbol{x}}_0$ we obtain

$$\begin{aligned}\left.\frac{\partial \mathrm{Var}_{\mathrm{a}}^{*}[k \mid \boldsymbol{x}_\bullet]}{\partial \zeta_\bullet}\right|_{\boldsymbol{x}_\bullet=\bar{\boldsymbol{x}}_0} = \left.\frac{\partial \mathrm{Var}_{\mathrm{o}}^{*}[k \mid \boldsymbol{x}_\bullet]}{\partial \zeta_\bullet}\right|_{\boldsymbol{x}_\bullet=\bar{\boldsymbol{x}}_0} &- \frac{2\,\eta_{\mathrm{s}}^2\,\mathrm{Var}_{\mathrm{o}}^{*}[k \mid \bar{\boldsymbol{x}}_0]\left.\frac{\partial \mathrm{Var}_{\mathrm{o}}^{*}[k|\boldsymbol{x}_\bullet]}{\partial \zeta_\bullet}\right|_{\boldsymbol{x}_\bullet=\bar{\boldsymbol{x}}_0}}{\tilde{s}\big(\mathbb{E}_{\mathrm{o}}^{*}[k \mid \bar{\boldsymbol{x}}_0]\big)^2}\\ &+\frac{2\,\eta_{\mathrm{s}}^3\,\mathrm{Var}_{\mathrm{o}}^{*}[k \mid \bar{\boldsymbol{x}}_0]^2\left.\frac{\partial \mathbb{E}_{\mathrm{o}}^{*}[k|\boldsymbol{x}_\bullet]}{\partial \zeta_\bullet}\right|_{\boldsymbol{x}_\bullet=\bar{\boldsymbol{x}}_0}}{\tilde{s}\big(\mathbb{E}_{\mathrm{o}}^{*}[k \mid \bar{\boldsymbol{x}}_0]\big)^3},\end{aligned} \tag{C.8}$$

and

$$\begin{aligned}\left.\frac{\partial \mathrm{Var}_{\mathrm{o}}^{*}[k \mid \boldsymbol{x}_\bullet]}{\partial \zeta_\bullet}\right|_{\boldsymbol{x}_\bullet=\bar{\boldsymbol{x}}_0} &= \left.\frac{\partial V_{\mathrm{l}}(\boldsymbol{x}_\bullet)}{\partial \zeta_\bullet}\right|_{\boldsymbol{x}_\bullet=\bar{\boldsymbol{x}}_0} + h_{\mathrm{vl}}(\bar{\boldsymbol{x}}_0)^2\left.\frac{\partial \mathrm{Var}_{\mathrm{a}}^{*}[k \mid \boldsymbol{x}_\bullet]}{\partial \zeta_\bullet}\right|_{\boldsymbol{x}_\bullet=\bar{\boldsymbol{x}}_0}\\ h_{\mathrm{vl}}(\bar{\boldsymbol{x}}_0)^2&\left[-\frac{2\,\eta_{\mathrm{f}}^2\,\mathrm{Var}_{\mathrm{a}}^{*}[k \mid \bar{\boldsymbol{x}}_0]\left.\frac{\partial \mathrm{Var}_{\mathrm{a}}^{*}[k|\boldsymbol{x}_\bullet]}{\partial \zeta_\bullet}\right|_{\boldsymbol{x}_\bullet=\bar{\boldsymbol{x}}_0}}{\big(f_0+\eta_{\mathrm{f}}\,\mathbb{E}_{\mathrm{a}}^{*}[k \mid \bar{\boldsymbol{x}}_0]\big)^2}+\frac{2\,\eta_{\mathrm{f}}^3\,\mathrm{Var}_{\mathrm{a}}^{*}[k \mid \bar{\boldsymbol{x}}_0]^2\left.\frac{\partial \mathbb{E}_{\mathrm{a}}^{*}[k|\boldsymbol{x}_\bullet]}{\partial \zeta_\bullet}\right|_{\boldsymbol{x}_\bullet=\bar{\boldsymbol{x}}_0}}{\big(f_0+\eta_{\mathrm{f}}\,\mathbb{E}_{\mathrm{a}}^{*}[k \mid \bar{\boldsymbol{x}}_0]\big)^3}\right].\end{aligned} \tag{C.9}$$

By substituting the expression of $\left.\frac{\partial \mathbb{E}_{\mathrm{a}}^{*}[k|\boldsymbol{x}_\bullet]}{\partial \zeta_\bullet}\right|_{\boldsymbol{x}_\bullet=\bar{\boldsymbol{x}}_0}$ and $\left.\frac{\partial \mathbb{E}_{\mathrm{o}}^{*}[k|\boldsymbol{x}_\bullet]}{\partial \zeta_\bullet}\right|_{\boldsymbol{x}_\bullet=\bar{\boldsymbol{x}}_0}$ from eqs. (C.6) and (C.7) into eqs. (C.8) and (C.9) and by solving for $\left.\frac{\partial \mathrm{Var}_{\mathrm{a}}^{*}[k|\boldsymbol{x}_\bullet]}{\partial \zeta_\bullet}\right|_{\boldsymbol{x}_\bullet=\bar{\boldsymbol{x}}_0}$ and $\left.\frac{\partial \mathrm{Var}_{\mathrm{o}}^{*}[k|\boldsymbol{x}_\bullet]}{\partial \zeta_\bullet}\right|_{\boldsymbol{x}_\bullet=\bar{\boldsymbol{x}}_0}$ we obtain

$$\begin{aligned}\left.\frac{\partial \mathrm{Var}_{\mathrm{a}}^{*}[k \mid \boldsymbol{x}_\bullet]}{\partial \zeta_\bullet}\right|_{\boldsymbol{x}_\bullet=\bar{\boldsymbol{x}}_0} \approx \frac{1}{D}\Bigg[&h_{\mathrm{vl}}(\bar{\boldsymbol{x}}_0)\\ &\times\left(2\left(\frac{\eta_{\mathrm{s}}}{\tilde{s}\big(\mathbb{E}_{\mathrm{o}}^{*}[k \mid \bar{\boldsymbol{x}}_0]\big)}\right)^4\mathrm{Var}_{\mathrm{o}}^{*}[k \mid \bar{\boldsymbol{x}}_0]^2+2\left(\frac{\eta_{\mathrm{s}}}{\tilde{s}\big(\mathbb{E}_{\mathrm{o}}^{*}[k \mid \bar{\boldsymbol{x}}_0]\big)}\right)^2\mathrm{Var}_{\mathrm{o}}^{*}[k \mid \bar{\boldsymbol{x}}_0]-1\right)\\ &-2\left(\frac{\eta_{\mathrm{s}}}{\tilde{s}\big(\mathbb{E}_{\mathrm{o}}^{*}[k \mid \bar{\boldsymbol{x}}_0]\big)}\right)^2\mathrm{Var}_{\mathrm{o}}^{*}[k \mid \bar{\boldsymbol{x}}_0]+1\Bigg]\left.\frac{\partial V_{\mathrm{l}}(\boldsymbol{x}_\bullet)}{\partial \zeta_\bullet}\right|_{\boldsymbol{x}_\bullet=\bar{\boldsymbol{x}}_0},\end{aligned} \tag{C.10}$$

and

$$\left.\frac{\partial \mathrm{Var}_{\mathrm{o}}^{*}[k \mid \boldsymbol{x}_{\bullet}]}{\partial \zeta_{\bullet}}\right|_{\boldsymbol{x}_{\bullet}=\bar{\boldsymbol{x}}_{0}} \approx \frac{1}{D}\left[1-2h_{\mathrm{vl}}(\bar{\boldsymbol{x}}_0)\frac{\eta_{\mathrm{f}}}{f_0+\eta_{\mathrm{f}}\,\mathbb{E}_{\mathrm{a}}^{*}[k \mid \bar{\boldsymbol{x}}_0]}\left(\frac{\eta_{\mathrm{s}}}{\tilde{s}\big(\mathbb{E}_{\mathrm{o}}^{*}[k \mid \bar{\boldsymbol{x}}_0]\big)}\right)^{3}\mathrm{Var}_{\mathrm{o}}^{*}[k \mid \bar{\boldsymbol{x}}_0]^{2}-h_{\mathrm{vl}}(\bar{\boldsymbol{x}}_0)\right] \times \left.\frac{\partial V_{\mathrm{l}}(\boldsymbol{x}_{\bullet})}{\partial \zeta_{\bullet}}\right|_{\boldsymbol{x}_{\bullet}=\bar{\boldsymbol{x}}_{0}}, \quad \text{(C.11)}$$

where

$$\begin{aligned} D = {} & 1-h_{\mathrm{vl}}(\bar{\boldsymbol{x}}_0)\left[1+2\frac{\eta_{\mathrm{f}}}{f_0+\eta_{\mathrm{f}}\,\mathbb{E}_{\mathrm{a}}^{*}[k \mid \bar{\boldsymbol{x}}_0]}\left(\frac{\eta_{\mathrm{s}}}{\tilde{s}\big(\mathbb{E}_{\mathrm{o}}^{*}[k \mid \bar{\boldsymbol{x}}_0]\big)}\right)^{3}\mathrm{Var}_{\mathrm{o}}^{*}[k \mid \bar{\boldsymbol{x}}_0]^{2}\right] \\ & + h_{\mathrm{vl}}(\bar{\boldsymbol{x}}_0)^{2}\Bigg[-2\left(\frac{\eta_{\mathrm{f}}}{f_0+\eta_{\mathrm{f}}\,\mathbb{E}_{\mathrm{a}}^{*}[k \mid \bar{\boldsymbol{x}}_0]}\right)^{3}\left(\frac{\eta_{\mathrm{s}}}{\tilde{s}\big(\mathbb{E}_{\mathrm{o}}^{*}[k \mid \bar{\boldsymbol{x}}_0]\big)}\right)\mathrm{Var}_{\mathrm{a}}^{*}[k \mid \bar{\boldsymbol{x}}_0]^{2} \\ & + \left(\frac{\eta_{\mathrm{f}}}{f_0+\eta_{\mathrm{f}}\,\mathbb{E}_{\mathrm{a}}^{*}[k \mid \bar{\boldsymbol{x}}_0]}\right)^{2}\mathrm{Var}_{\mathrm{a}}^{*}[k \mid \bar{\boldsymbol{x}}_0]\left(2-4\left(\frac{\eta_{\mathrm{s}}}{\tilde{s}\big(\mathbb{E}_{\mathrm{o}}^{*}[k \mid \bar{\boldsymbol{x}}_0]\big)}\right)^{2}\mathrm{Var}_{\mathrm{o}}^{*}[k \mid \bar{\boldsymbol{x}}_0]\right) \\ & + 2\left(\frac{\eta_{\mathrm{s}}}{\tilde{s}\big(\mathbb{E}_{\mathrm{o}}^{*}[k \mid \bar{\boldsymbol{x}}_0]\big)}\right)^{2}\mathrm{Var}_{\mathrm{o}}^{*}[k \mid \bar{\boldsymbol{x}}_0]-1\Bigg] \\ & h_{\mathrm{vl}}(\bar{\boldsymbol{x}}_0)^{3}\left(1-2\left(\frac{\eta_{\mathrm{f}}}{f_0+\eta_{\mathrm{f}}\,\mathbb{E}_{\mathrm{a}}^{*}[k \mid \bar{\boldsymbol{x}}_0]}\right)^{4}\mathrm{Var}_{\mathrm{a}}^{*}[k \mid \bar{\boldsymbol{x}}_0]^{2}-2\left(\frac{\eta_{\mathrm{f}}}{f_0+\eta_{\mathrm{f}}\,\mathbb{E}_{\mathrm{a}}^{*}[k \mid \bar{\boldsymbol{x}}_0]}\right)^{2}\mathrm{Var}_{\mathrm{a}}^{*}[k \mid \bar{\boldsymbol{x}}_0]\right) \\ & \times\left(1-2\left(\frac{\eta_{\mathrm{s}}}{\tilde{s}\big(\mathbb{E}_{\mathrm{o}}^{*}[k \mid \bar{\boldsymbol{x}}_0]\big)}\right)^{4}\mathrm{Var}_{\mathrm{o}}^{*}[k \mid \bar{\boldsymbol{x}}_0]^{2}-2\left(\frac{\eta_{\mathrm{s}}}{\tilde{s}\big(\mathbb{E}_{\mathrm{o}}^{*}[k \mid \bar{\boldsymbol{x}}_0]\big)}\right)^{2}\mathrm{Var}_{\mathrm{o}}^{*}[k \mid \bar{\boldsymbol{x}}_0]\right). \quad \text{(C.12)} \end{aligned}$$

Given that the variance in knowledge of both adults and offspring is small, and using the expression of $V_{\mathrm{l}}(\boldsymbol{x}_{\bullet})$ from eq. (A.47) with $\sigma_{\mathrm{i}} = \sigma_{\mathrm{i}}(\zeta_{\bullet})$, we obtain

$$\boxed{\left.\frac{\partial \mathrm{Var}_{\mathrm{a}}^{*}[k \mid \boldsymbol{x}_{\bullet}]}{\partial \zeta_{\bullet}}\right|_{\boldsymbol{x}_{\bullet}=\bar{\boldsymbol{x}}_{0}} \approx \left.\frac{\partial \mathrm{Var}_{\mathrm{o}}^{*}[k \mid \boldsymbol{x}_{\bullet}]}{\partial \zeta_{\bullet}}\right|_{\boldsymbol{x}_{\bullet}=\bar{\boldsymbol{x}}_{0}} \approx \frac{2\bar{\lambda}^{2}\,\sigma_{\mathrm{i}}(0)\sigma_{\mathrm{i}}'(0)\,(1-\bar{v}-\bar{o})}{1-h_{\mathrm{vl}}(\bar{\boldsymbol{x}}_0)^{2}}.} \quad \text{(C.13)}$$

Since the right-hand side of eq. (C.13) is always positive, it shows that, in the $\boldsymbol{x}_{\bullet}$-lineage, the trait $\zeta_{\bullet}$ consistently increases the variance of knowledge among randomly chosen adults and offspring within the lineage. Moreover, from eqs. (C.6) and (C.7), we infer that $\zeta_{\bullet}$ also consistently increases their expected knowledge. Consequently, eq. (C.1) always holds, and selection systematically favors the emergence of the trait ζ.

Appendix D: Individual-based simulations

Our individual-based simulations track the evolution of the trait distribution, the distribution of individual knowledge, and the population size at each generation for a fixed number of generations following the life cycle described in the main text. Each individual i at each generation is characterised by the vector of its three traits (v_i, o_i, λ_i), as well as its knowledge k_i at the end of the learning period, the knowledge of its parent $k_{\mathrm{p,i}}$ and its oblique exemplar $k_{\mathrm{a,i}}$. At the first generation, the values of v_i, o_i, λ_i, $k_{\mathrm{p,i}}$ and $k_{\mathrm{a,i}}$ are by default all initialized to 0 for each individual $i \in \{1, \ldots, n_1\}$, where $n_1 = 100$ is the number of individuals generation 1.

At each generation $t \geq 1$, the following occurs:

(i) *Reproduction.* We first determine the fecundity f_i of each individual $i \in \{1, ..., n_t\}$, where n_t is the number of adult individuals at generation t. The fecundity of each individual i, is calculated using $f_i = f((v_i, o_i, \lambda_i), k_i)$, where the expression of $f((v_i, o_i, \lambda_i), k_i)$ is given in eq. (1) with $\boldsymbol{x}_\bullet = (v_i, o_i, \lambda_i)$ and $k_{\mathrm{p}\bullet} = k_i$. Each individual i then produces a number of offspring that is sampled from a Poisson probability density with mean equal to f_i. In total, they produce a total number of offspring that we denote as $n_{\mathrm{o},t}$.

(ii) *Mutation.* With probability $1 - \mu$, an offspring inherits the same traits as its parent. Otherwise, with probability μ, traits mutate; we model this by adding an effect sampled from a normal probability density with mean 0 and variance σ^2 to each parental trait value. If necessary, the resulting trait values are truncated to remain in $\{(v, o, \lambda) : 0 \leq v \leq 1, 0 \leq o \leq 1, 0 \leq v + o \leq 1, 0 \leq \lambda \leq 1\}$. In all simulations, we set $\mu = 0.01$ and $\sigma = 0.01$.

(iii) *Learning.* For each offspring, i, an oblique exemplar is randomly sampled from the adults, with its knowledge denoted as $k_{\mathrm{a,i}}$. The knowledge k_i of each offspring i after learning is complete, for $i \in \{1, ..., n_{\mathrm{o},t}\}$, is determined by $k_i = k_\bullet(1)$ where $k_\bullet(1)$ is computed by solving numerically the stochastic differential equation eq. (2) with $\boldsymbol{x}_\bullet = (v_i, o_i, \lambda_i)$, $k_{\mathrm{p}\bullet} = k_{\mathrm{p,i}}$ and $k_{\mathrm{a}\bullet} = k_{\mathrm{a,i}}$ using the *sdeint.itoint* function from the Python package *sdeint*. To reduce computation time when $\sigma_{\mathrm{v}} = \sigma_{\mathrm{o}} = 0$, we instead compute $k_\bullet(1)$ using eq. (A.6) with $\boldsymbol{x}_\bullet = (v_i, o_i, \lambda_i)$, $k_{\mathrm{p}\bullet} = k_{\mathrm{p,i}}$ and $k_{\mathrm{a}\bullet} = k_{\mathrm{a,i}}$, which provides an explicit expression for the final knowledge after learning, thereby avoiding numerical integration of the stochastic differential equation eq. (2).

(iv) *Survival.* All parents die and each offspring i in $\{1, ..., n_{\mathrm{o},t}\}$ then survives till adulthood with probability $s(k_i, n_{\mathrm{o},t})$ (and otherwise dies). The survival probability of each offspring i is

calculated using the expression of $s(k_i, n_{o,t})$ given by substituting the expression of $\tilde{s}(k_i)$ from eq. (4) with $k_{o\bullet} = k_i$ into eq. (3) with $k_{o\bullet} = k_i$ and $n_o = n_{o,t}$. This results in the number n_{t+1} of adults of the next generation (with $n_{t+1} \leq n_{o,t}$).

We repeat steps (i)-(iv) for a fixed number of generations (see figure legends for parameter values).

References

R. F. Bass. *Stochastic Processes.* Cambridge Series in Statistical and Probabilistic Mathematics. Cambridge University Press, 2011.

C. Gardiner. *Handbook of Stochastic Methods for Physics, Chemistry, and the Natural Sciences.* Proceedings in Life Sciences. Springer-Verlag, 1985.

S. A. H. Geritz, J. A. J. Metz, and C. Rueffler. Mutual invadability near evolutionarily singular strategies for multivariate traits, with special reference to the strongly convergence stable case. *Journal of Mathematical Biology*, 72(4):1081–1099, 2016.

T. E. Harris. *The Theory of Branching Processes.* Springer, Berlin, 1963.

Y. Iwasa, A. Pomiankowski, and S. Nee. The evolution of costly mate preferences II. The 'handicap' principle. *Evolution*, 45:1431–1442, 1991.

Y. Kobayashi, H. Ohtsuki, and J. Y. Wakano. Population size vs. social connectedness — a gene-culture coevolutionary approach to cumulative cultural evolution. *Theoretical Population Biology*, 111:87–95, 2016.

L. Lehmann, C. Mullon, E. Akçay, and J. Van Cleve. Invasion fitness, inclusive fitness, and reproductive numbers in heterogeneous populations. *Evolution*, 70(8):1689–1702, 2016.

L. Maisonneuve, L. Lehmann, and C. Mullon. The coevolution of learning schedules and teaching enhances cumulative knowledge and drives a teacher–innovator syndrome. *Proceedings of the Royal Society B: Biological Sciences*, 292(2040):20242470, 2025.

C. Mullon and L. Lehmann. An evolutionary quantitative genetics model for phenotypic (co)variances under limited dispersal, with an application to socially synergistic traits. *Evolution*, 73(9):1695–1728, 2019.

E Supplementary figures

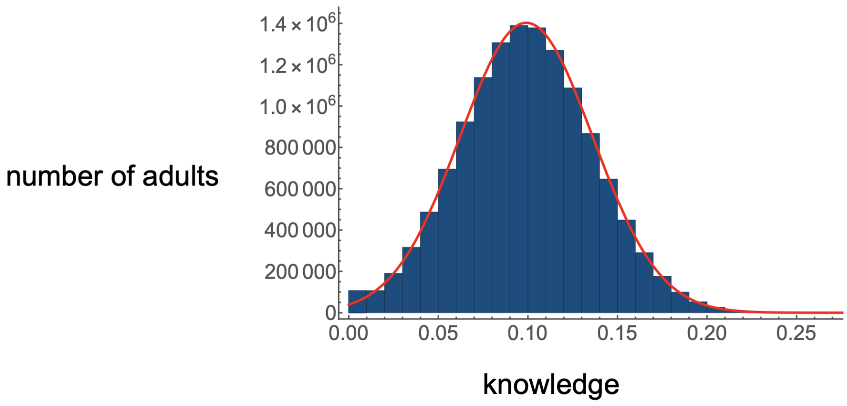

Figure S.1: **Stationary distribution of adults knowledge from individual-based simulation.** The histogram shows the distribution of adult knowledge over 100,000 generations at equilibrium, based on individual-based simulations. The population was first allowed to evolve for 100,000 generations to ensure that both cultural and evolutionary equilibria were reached. The red curve represents the distribution that would be expected if adult knowledge were normally distributed, with the mean and variance of the Gaussian distribution matching those observed in the simulation, illustrating that a Gaussian approximation provides a good fit for the distribution of knowledge in the population. Parameters are: $f_0 = 5$, $s_0 = 1$, $\beta_{\rm v} = 1.4$, $\beta_{\rm o} = 1.3$, $\alpha = 0.1$, $\epsilon = 0.05$, $\rho = 0.05$, $\sigma_{\rm v} = \sigma_{\rm o} = 0$, $\sigma_{\rm i} = 0.05$, $\eta_{\rm f} = 25$, $\eta_{\rm s} = 5$, $\gamma = 0.01$, $\theta = 0.1$.

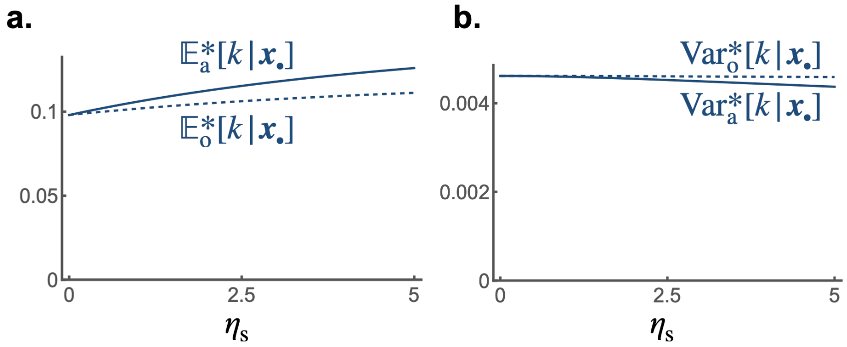

Figure S.2: **The impact of the conversion factors that translate knowledge into survival benefits $\eta_{\rm s}$ on the expected knowledge and variance in knowledge at cultural equilibrium for a random adult and offspring of the $\boldsymbol{x}_\bullet$-lineage. a** $\mathbb{E}^*_{\rm a}[k \mid \boldsymbol{x}_\bullet]$ (blue solid line) and $\mathbb{E}^*_{\rm o}[k \mid \boldsymbol{x}_\bullet]$ (blue dashed line) and **b** $\text{Var}^*_{\rm a}[k \mid \boldsymbol{x}_\bullet]$ (blue solid line) and $\text{Var}^*_{\rm o}[k \mid \boldsymbol{x}_\bullet]$ (blue dashed line) according to $\eta_{\rm s}$. This shows that the expected knowledge in adults is slightly higher than in offspring, while the variance in adult knowledge is slightly lower than in offspring. Default parameters are the same as in fig. 2 with $\boldsymbol{x}_\bullet = \bar{\boldsymbol{x}}$.

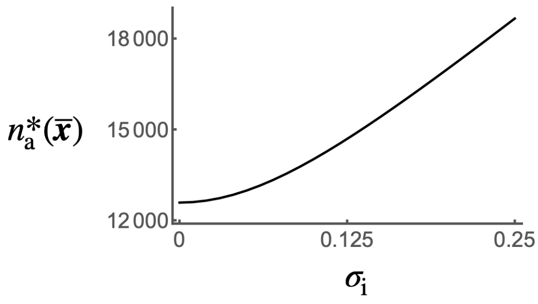

Figure S.3: **The impact of stochasticity in individual learning on population size.** Equilibrium adult population size $n_a^*(\bar{\boldsymbol{x}})$ according to σ_i. This shows that the population size increases with stochasticity in individual learning. Default parameters are the same as in fig. 2 with $\gamma = 10^{-4}$.

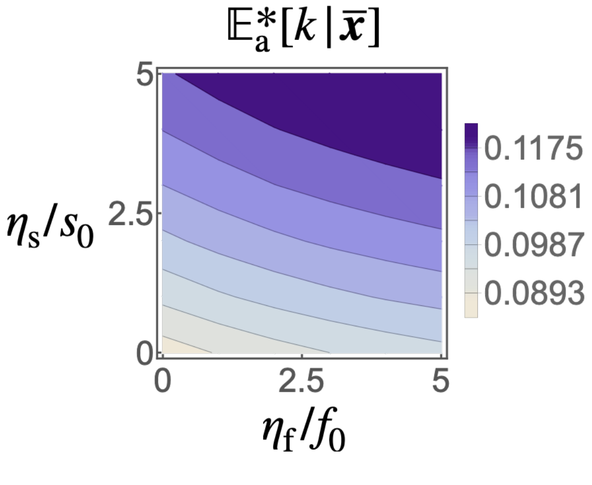

Figure S.4: **Impact of selective pressure on the population mean knowledge.** Population mean knowledge at cultural equilibrium $\mathbb{E}_a^*[k \mid \bar{\boldsymbol{x}}]$ according to η_f and η_s. This shows that the effect of selection in enhancing the mean knowledge $\mathbb{E}_a^*[k \mid \bar{\boldsymbol{x}}]$ is stronger when η_f and η_s are higher. Default parameters are the same as in fig. 2.

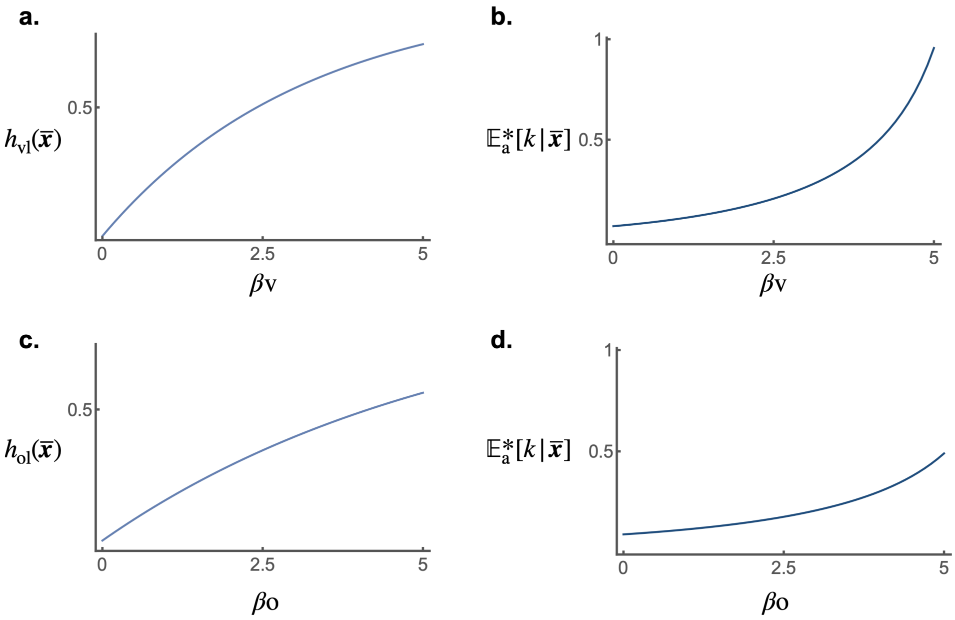

Figure S.5: **Impact of transmission on the population mean knowledge.** Population mean **a** vertical cultural heritability $h_{\mathrm{vl}}(\bar{\boldsymbol{x}})$ and **b** knowledge at cultural equilibrium $\mathbb{E}_{\mathrm{a}}^{*}[k \mid \bar{\boldsymbol{x}}]$ according to β_{v}. Population mean **c** oblique cultural heritability $h_{\mathrm{ol}}(\bar{\boldsymbol{x}})$ and **d** knowledge at cultural equilibrium $\mathbb{E}_{\mathrm{a}}^{*}[k \mid \bar{\boldsymbol{x}}]$ according to β_{o}. This shows that the effect of selection in enhancing the mean knowledge $\mathbb{E}_{\mathrm{a}}^{*}[k \mid \bar{\boldsymbol{x}}]$ is stronger when intergenerational transmission is higher. Default parameters are the same as in fig. 2.

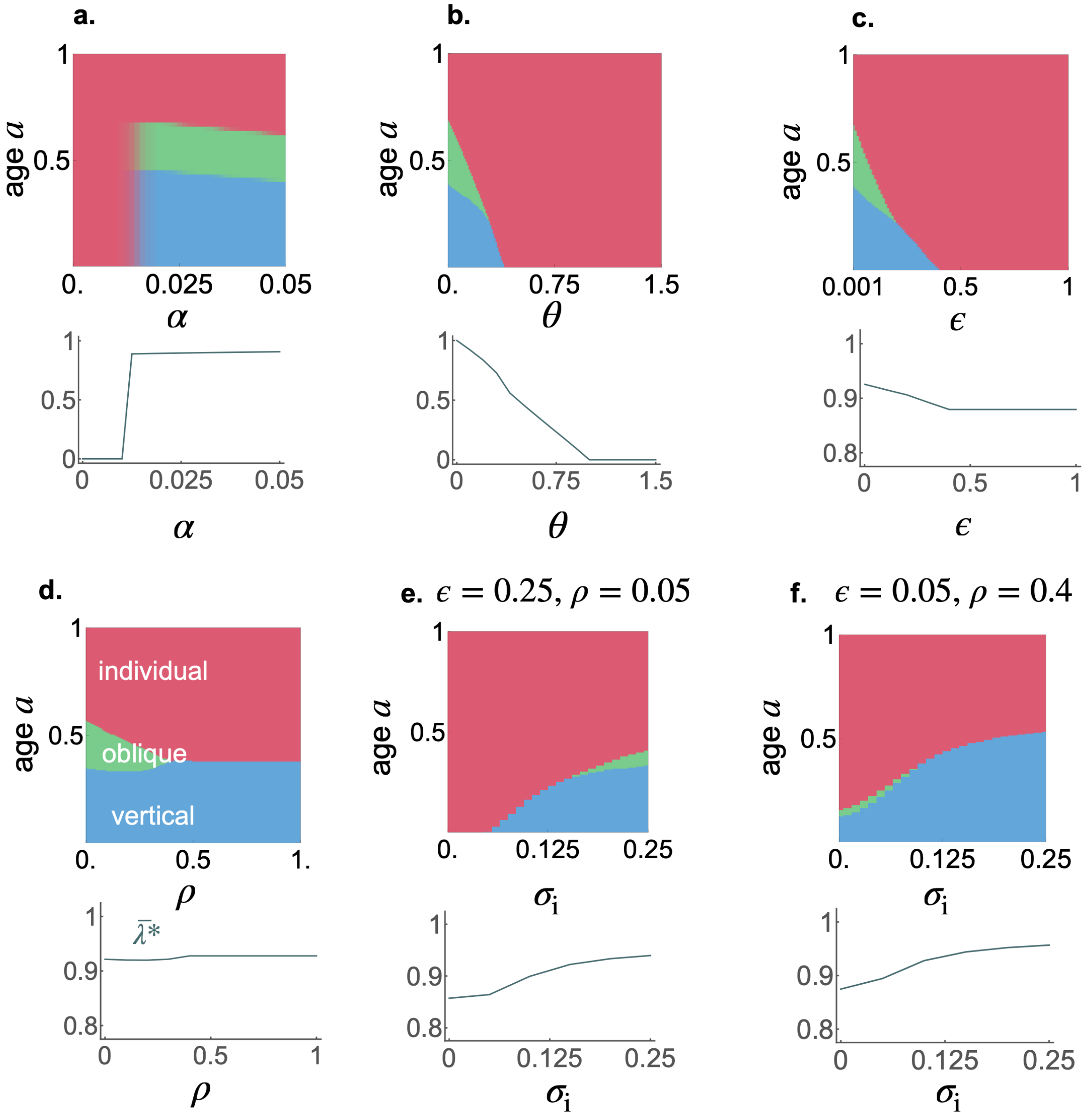

Figure S.6: **Factors impacting the evolution of learning traits.** Learning schedule and investment in learning $\bar{\lambda}^*$ at $\bar{\boldsymbol{x}}^*$ against **a** α, **b** θ, **c** ϵ, **d** ρ, and **e-f** $\sigma_{\rm i}$. Blue, green, and pink areas represent time spent performing vertical, oblique, and individual learning, respectively. Default parameters are the same as in fig. **??**, except in panel e, where $\epsilon = 0.25$ and in panel f, where $\rho = 0.4$.

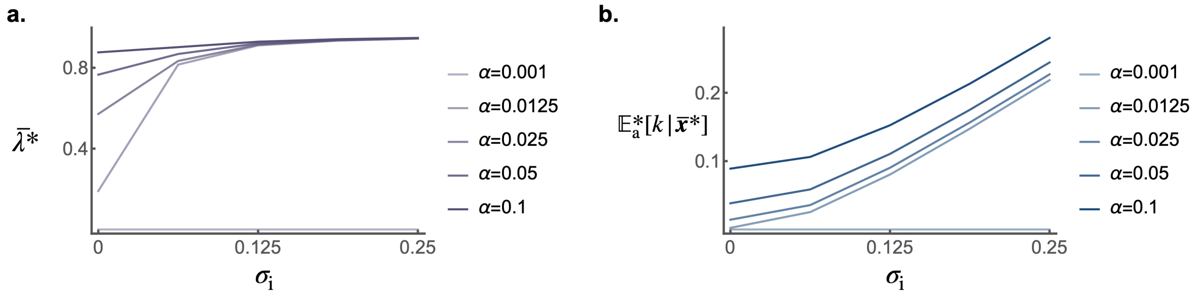

Figure S.7: **Impact of stochasticity in individual learning $\sigma_{\rm i}$ on investment in learning $\bar{\lambda}^*$ and the population mean knowledge $\mathbb{E}_{\rm a}^*[k \mid \bar{\boldsymbol{x}}^*]$ at $\bar{\boldsymbol{x}}^*$ for different values of α.** This shows that stochasticity in learning can promote both higher average knowledge and greater investment in learning, except in populations with very low values of α. Default parameters are the same as in fig. **??**.

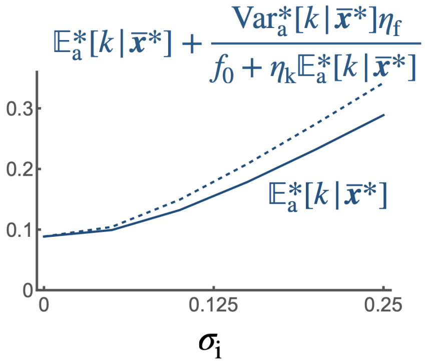

Figure S.8: **Impact of stochasticity in individual learning $\sigma_{\rm i}$ on the average knowledge of parents $\mathbb{E}_{\rm a}^*[k \mid \bar{\boldsymbol{x}}^*] + \mathrm{Var}_{\rm a}^*[k \mid \bar{\boldsymbol{x}}^*]\, \eta_{\rm f} / \big(f_0 + \eta_{\rm f}\, \mathbb{E}_{\rm a}^*[k \mid \bar{\boldsymbol{x}}^*]\big)$ and of oblique exemplars $\mathbb{E}_{\rm a}^*[k \mid \bar{\boldsymbol{x}}^*]$ at $\bar{\boldsymbol{x}}^*$.** This shows that under higher stochasticity in individual learning, the average knowledge of parents increases more than that of oblique exemplars. Default parameters are the same as in fig. **??**.

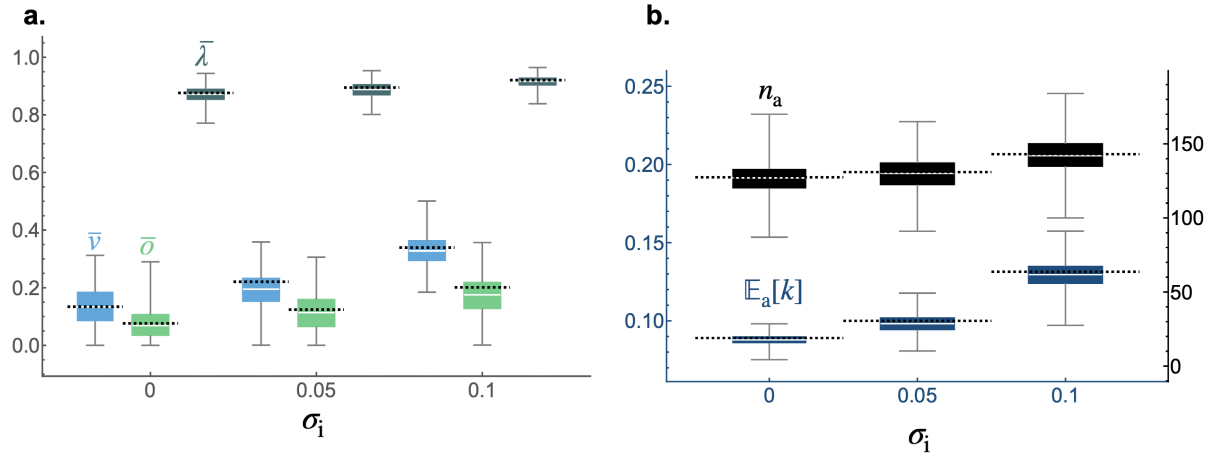

Figure S.9: **Impact of stochasticity in individual learning on the stationary distribution of mean traits, average adults knowledge, and adult population size in individual-based simulations.** Box plots show distributions of **a** mean traits $\bar{\boldsymbol{x}} = (\bar{v}, \bar{o}, \bar{\lambda})$ and **b** average adults knowledge $\mathbb{E}_a(k)$ and adult population size n_a over 400,000 generations at equilibrium from individual-based simulations for different values of the strength σ_i of stochasticity in individual learning. For each value of σ_i, the population was first allowed to evolve for 100,000 generations to ensure equilibrium was reached. The box shows the interquartile range (IQR) with the median marked inside; whiskers extend to the smallest and largest values within $1.5 \times$ IQR from Q1 and Q3, respectively. Dashed lines show the predicted values derived from evolutionary analyses. The close match between simulation outcomes and analytical predictions suggests a good level of agreement between the two approaches. Default parameters are the same as in fig. 3 with $\sigma_v = \sigma_o = 0$ and $\gamma = 0.01$.

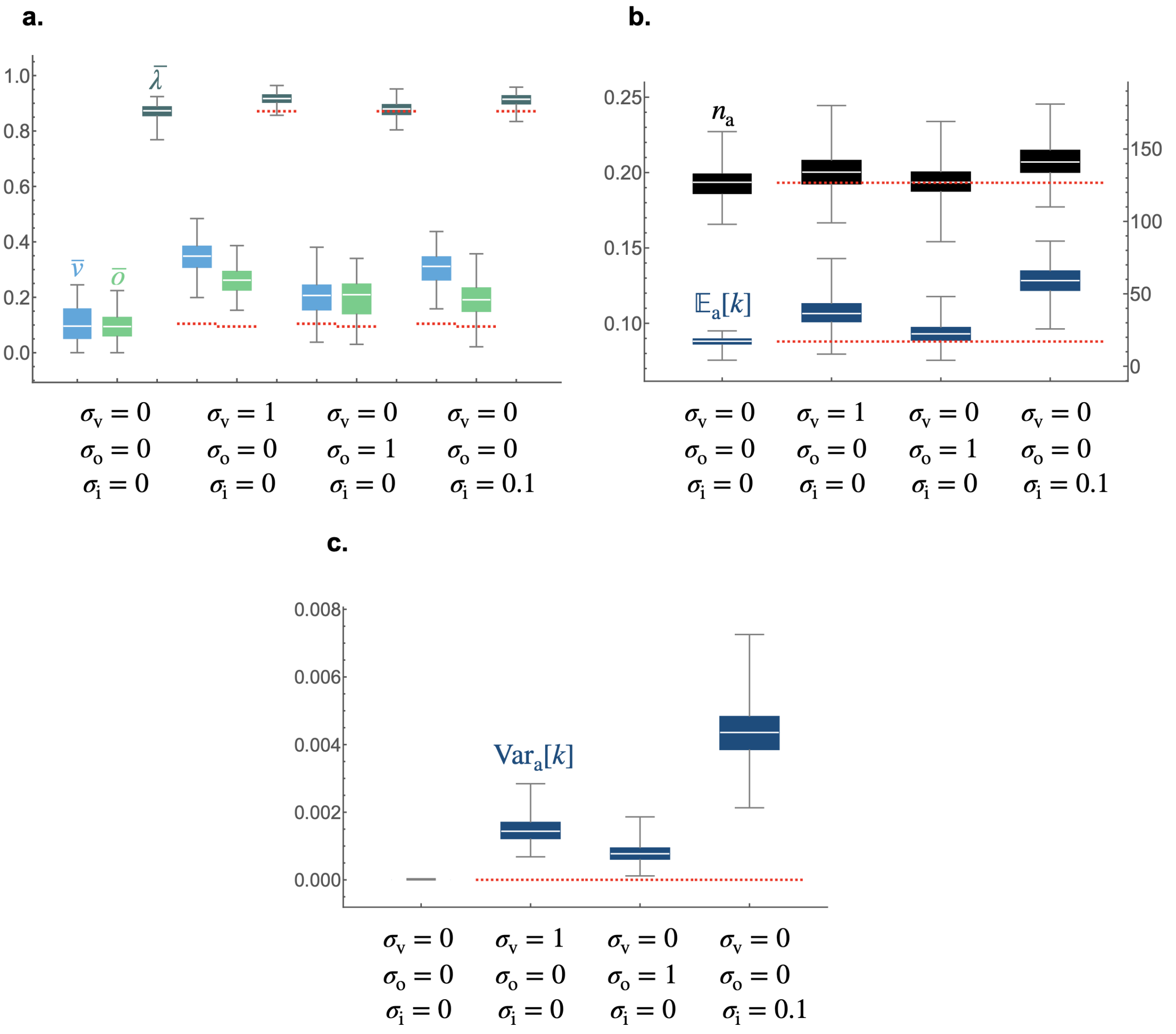

Figure S.10: **Impact of stochasticity in the different types of learning on the stationary distribution of mean traits, average adults knowledge, and adult population size in individual-based simulations.** Box plots show distributions of **a** mean traits $\bar{\boldsymbol{x}} = (\bar{v}, \bar{o}, \bar{\lambda})$, **b** average adults knowledge $\mathbb{E}_a(k)$ and adult population size n_a and **c** adults knowledge variance $\mathrm{Var}_a(k)$ over 100,000 generations at equilibrium from individual-based simulations for different values of strength of stochasticity in vertical σ_v, oblique σ_o, and individual σ_i learning. For each combination of values for σ_v, σ_o, and σ_i, the population was first allowed to evolve for 100,000 generations to ensure equilibrium was reached. Because individual-based simulations run significantly slower when $\sigma_v > 0$ or $\sigma_o > 0$ (due to the need to numerically solve the stochastic differential equation eq. (2)), we initialize trait values for each individual i in the first generation as $(v_i, o_i, \lambda_i) = (0.13404, 0.0762715, 0.876229)$. These correspond to the mean trait values predicted at $\bar{\boldsymbol{x}}^*$ in the absence of stochasticity in learning (i.e., when $\sigma_v = \sigma_o = \sigma_i = 0$). The box shows the interquartile range (IQR) with the median marked inside; whiskers extend to the smallest and largest values within 1.5 $\times$ IQR from Q1 and Q3, respectively. Red dashed lines show the mean values obtained for individual-based simulation in the absence of stochasticity in learning. This shows that, similarly to individual learning, greater stochasticity in vertical and oblique learning promotes the accumulation of knowledge, an increase in population size, and a greater allocation of time to social learning. Note that these effects are less pronounced for stochasticity in oblique learning, because it generates less variance in knowledge and therefore weaker cultural selection. This likely occurs because the amount of knowledge acquired through oblique learning is lower, so stochasticity produces less variation overall. Default parameters are the same as in fig. 3 with $\gamma = 0.01$.
